# A General Signal Pathway to Regulate Multiple Detoxification Genes Drives the Evolution of *Helicoverpa armigera* Adaptation to Xenobiotics

**DOI:** 10.3390/ijms232416126

**Published:** 2022-12-17

**Authors:** Lei Zhang, Shenglan Lv, Mingjian Li, Meng Gu, Xiwu Gao

**Affiliations:** College of Plant Protection, China Agricultural University, Beijing 100193, China

**Keywords:** xenobiotics, detoxification genes, transcription factor, regulation

## Abstract

The study of insect adaptation to the defensive metabolites of host plants and various kinds of insecticides in order to acquire resistance is a hot topic in the pest-control field, but the mechanism is still unclear. In our study, we found that a general signal pathway exists in *H. armigera* which can regulate multiple P450s, GSTs and UGTs genes to help insects decrease their susceptibility to xenobiotics. Knockdown of *HaNrf2* and *HaAhR* expression could significantly increase the toxicity of xenobiotics to *H. armigera*, and simultaneously decrease the gene expression of P450s, GSTs and UGTs which are related to the xenobiotic metabolism and synthesis of insect hormone pathways. Then, we used EMSA and dual luciferase assay to verify that a crosstalk exists between AhR and Nrf2 to regulate multiple P450s, GSTs and UGTs genes to mediate *H. armigera* susceptibility to plant allelochemicals and insecticides. The detoxification genes’ expression network which can be regulated by Nrf2 and AhR is still unknown, and there were also no reports about the crosstalk between AhR and Nrf2 that exist in insects and can regulate multiple detoxification genes’ expression. Our results provide a new general signaling pathway to reveal the adaptive mechanism of insects to xenobiotics and provides further insight into designing effective pest-management strategies to avoid the overuse of insecticides.

## 1. Introduction

The study of insect adaptation to defensive metabolites in host plants and varying kinds of insecticides in order to acquire resistance is a popular focus in the pest-control field, however, the mechanism of insect adaptation to these xenobiotics is still unclear [1,2]. Cotton bollworm (*Helicoverpa armigera*), as a herbivorous insect, has a wide range of host plant species, and can be harmful to cotton, tomato, maize, tobacco, as well as other crops. The evolutionary pressure of varied plants’ allelochemicals and insecticides have caused herbivorous insects to evolve corresponding countermeasures [1,2].

Cotton, being one of the primary host plants of *H. armigera*, has evolved efficient defense strategies against insects involving the synthesis of the toxic gossypol. Some insects cannot feed on cotton due to the toxic nature of gossypol, but *H. armigera* has apparently been successful in adapting to this toxin during a long evolutionary process. Solanaceae plants such as tomato use the allelochemical 2-tridecanone to defend against insects, but the cotton bollworm has successfully evolved and adapted to toxic 2-tridecanone. As there has been a widespread usage of various insecticides to control cotton bollworm, the insect has also developed a resistance to insecticides. The above problems lead to the failure of pest control. Increased detoxification enzyme activity is one of the most common strategies used by insects to resist the deleterious effects of xenobiotics [1,2]. Insect detoxification of insecticides and plant allelochemicals can be divided into three phases: phase I (oxidation, hydrolysis, reduction), phase II (conjugation), and phase III (excretion) [1,2]. In phase I, insecticides and/or plant allelochemicals are transformed into more hydrophilic metabolites by enzymes such as cytochrome P450 monooxygenases (P450s). P450s act to detoxify xenobiotics by introducing reactive or polar groups into the chemical structure [3]. In phase II, enzymes such as glutathione S transferases (GSTs) and UDP-glucuronosyltransferases (UGTs) conjugate the resulting metabolites of phase I to increase their hydrophilicity [4]. In phase III, after further conjugation, cleavage, and/or oxidation, the phase II-generated products are transported out of the cells by ATP-binding cassette (ABC) and other transmembrane transporters, where further degradation occurs [5,6]. Interestingly, many studies have found that multi over-expression of P450, GST, and UGT genes work together to mediate the plants allelochemicals tolerance and insecticide resistance in *H. armigera* [7].

Transcription factors play important roles in regulating detoxification genes involved in the xenobiotic metabolism [8]. However, the transcriptional regulation mechanism of these detoxification genes is still unclear, due to the vast number of detoxification enzyme genes present. In Drosophila, the nuclear receptor hormone DHR96 (receptor-like in 96) was found to regulate the expression of P450 genes associated with DDT resistance [9]. In *Bemisia tabaci*, a recent study reported that a P450 gene mediated resistance to imidacloprid by a mitogen-activated protein kinases (MAPKs) pathway [10]. Notably, recent studies in multiple insect species reported that insects have co-opted a xenobiotic stress response pathway that involves reactive oxygen species and the transcription factors Nrf2 (nuclear factor erythroid-2 related factor-2) or AhR (aryl hydrocarbon receptor) to up-regulate the expression of some detoxification gene members involved in insecticides resistance [11,12,13].

Nrf2 transcription factors are conserved in invertebrates, vertebrates, and metazoans [14]. Known as Cnc isoform C (CncC) in Drosophila, and skinhead family member 1 (SKN-1) in metazoans, Nrf2s are homologous proteins that play important roles in regulating cellular defenses against oxidative stressors or xenobiotics [6]. Normally, Nrf2 is retained in the cytoplasm by the actin-binding protein Keap1 (kelch-like ECH-associated protein 1) [15]. However, Keap1 acts as a stress sensor that can release Nrf2 into the nucleus under oxidative stress, after which Nrf2 binds to its heterodimer bZIP protein partner Maf (muscle aponeurosis fibromatosis). The heterodimer binds to xenobiotic response elements of target detoxification genes to regulate their expression [15]. AhR is a ligand-activated transcription factor that plays important roles in metabolic detoxification adaptation and normal physiology in mammals [16]. Metabolic detoxification adaptation is based on the coordinated regulation of some xenobiotic-metabolizing enzymes [3].

Despite some excellent studies on the single detoxification gene member induced by host plant allelochemicals or insecticides being regulated by Nrf2 or AhR, the network regarding the detoxification genes’ expression which can be regulated by Nrf2 and AhR is still unknown, and there were also no reports about the crosstalk between AhR and Nrf2 that exist in insects and can regulate the detoxification genes’ expression. The study of insect adaptation to defensive metabolites of host plants and various kinds of insecticides in order to acquire resistance is a hot topic in the pest-control field, but the mechanism is still unclear.

In this study, we found numerous P450s, GSTs, and UGTs gene promoter sequences which possess at least one Nrf2 binding site or AhR binding site in *H. armigera*. Some P450, GST, and UGT gene promoters simultaneously contain a very closely or overlapping Nrf2 and AhR binding sites. We then used RNA interference (RNAi) to investigate the function of *HaNrf2* and *HaAhR* in *H. armigera* susceptibility to the plant’s allelochemical (gossypol and 2-tridecanone) and insecticides. Employing RNA sequencing, quantitative polymerase chain reaction (qPCR), and Nrf2 and AhR binding site prediction, EMSA and dual luciferase assay, we determined that the expression of most of the detoxification genes’ expression is regulated by a general crosstalk between HaNrf2 and HaAhR. There were also no reports on the existence of the crosstalk between AhR and Nrf2 in insects or the proof that they both can regulate P450s, GSTs, and UGTs genes which were related to the xenobiotic metabolism or synthesis of insect hormone pathways. As is well known, a lot of plants allelochemicals or insecticides will not only have the phenotype to kill insects directly, but also show an ability to decrease the growth rate of the insects. However, the molecular mechanism of this phenotype is still unknown [1,17,18]. Our results suggest that a crosstalk exists between AhR and Nrf2 pathways in *H. armigera* to mediate the susceptibility to plant allelochemicals and insecticides. Our results provide a general signal pathway for revealing the adaptive mechanism of insects to xenobiotics, and explain why many xenobiotics have both the phenotype of toxic action and affect the decreasing growth rates in insects. The results of this study improve our understanding of detoxification genes regulation in *H. armigera* and provide more insight into designing effective pest-management strategies to avoid the overuse of insecticides. The transcription factors HaNrf2 and HaAhR could serve as potential targets of dsRNA spraying for pest management.

## 2. Results

### 2.1. Effects of RNAi HaNrf2 and HaAhR Genes on Xenobiotics Susceptibility of H. armigera 

In this study, we found that 1999 bp fragments upstream of the translation start sites (ATG) of all P450, GST and UGT genes, most of them were related by xenobiotic metabolism or synthesis of insect hormone pathways in *H. armigera*, and had at least one Nrf2 binding site or AhR binding site (Table S2). 

*HaNrf2* and *HaAhR* are essential genes for insect survival, so we investigated the effects of *HaNrf2* or *HaAhR* genes on the susceptibility of *H. armigera* to gossypol, 2-tridecanone or insecticides by RNAi knockdown method. In Lepidoptera, knockdown of the gene expression by RNAi has sometimes proven difficult to achieve, but gene silencing is easily achieved by feeding high concentrations dsRNA [19]. 

**Figure 1:**
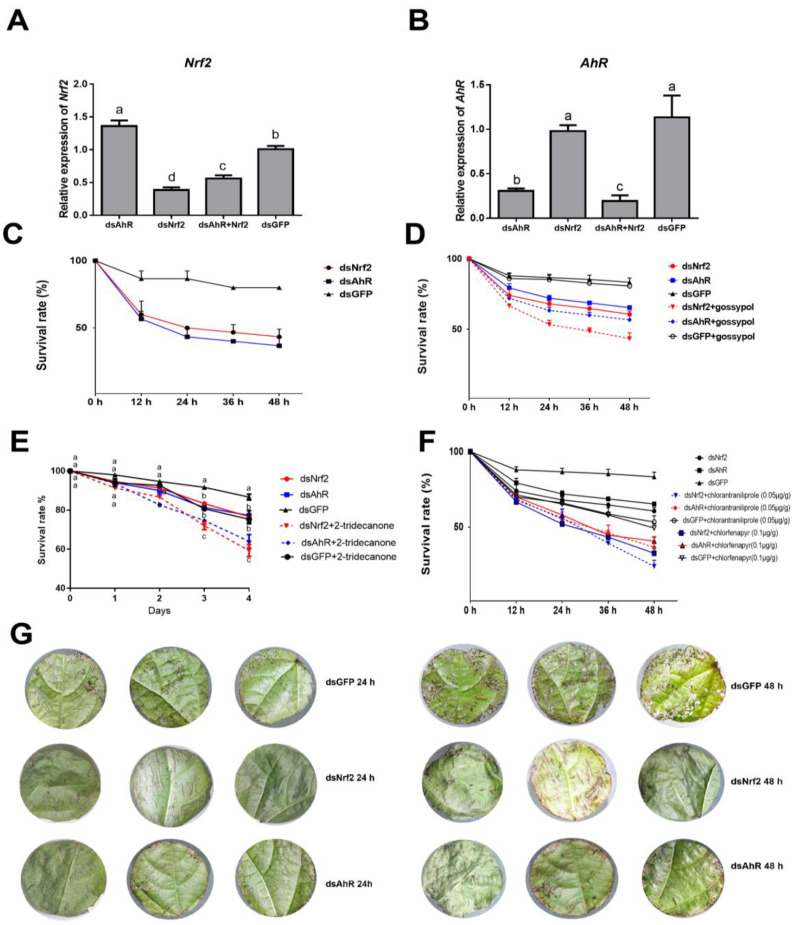
The effect on the susceptibility of xenobiotics by RNAi *HaNrf2* and *HaAhR* in *H. armigera.* (**A**) RT-qPCR analysis of *HaNrf2* gene expression after RNAi knockdown; (**B**) RT-qPCR analysis of *HaAhR* gene expression after RNAi knockdown; (**C**) mortality of *H. armigera* fed on cotton leaves coated with dsNrf2 or dsAhR; (**D**) mortality after *HaNrf2* and *HaAhR* knockdown in *H. armigera* fed an artificial diet containing 0.02 mg/g gossypol; (**E**) mortality after *HaNrf2* and *HaAhR* knockdown in *H. armigera* fed an artificial diet containing 0.05 mg/g 2-tridecanone; (**F**) mortality after *HaNrf2* and *HaAhR* knockdown in *H. armigera* fed an artificial diet containing 0.05 μg/g chlorantraniliprole or 0.1 μg/g chlorfenapyr; (**G**) resistance of dsNrf2 and dsAhR coated cotton leaves against *H. armigera*. Means and SEs from three biological replicates are shown. Different letters on error bars show significant differences (*p* < 0.05).

Full-length sequences of the *H. armigera HaNrf2* (KU355787.1) and *HaAhR* (XM_021331544.1) genes were cloned and sequenced. From these, dsRNAs were synthesized and fed to *H. armigera* larvae along with the plant allelochemical gossypol, 2-tridecanone or insecticides for 24 h. Afterwards, mortality was monitored and recorded. *HaNrf2* or *HaAhR* dsRNA-treated larvae showed significant reductions in *HaNrf2* or *HaAhR* gene expression compared to larvae treated with *GFP* dsRNA (Figure 1A,B). Compared to the control, *HaNrf2* gene expression was suppressed by 62% 24 h after feeding larvae an artificial diet containing 15 μg/g (*w:w*) of *HaNrf2* dsRNA, but the expression of *HaAhR* in dsNrf2-treated larvae was higher than in larvae treated with dsGFP control (Figure 1A). The transcription levels of *HaAhR* were suppressed (inhibition ratio over 70%) at 24 h after feeding with *HaAhR* dsRNA (15 μg/g) (Figure 1B). The larvae treated both with *HaNrf2* and *HaAhR* dsRNA generated the lowest *HaAhR* gene expression, but not the lowest *HaNrf2* gene expression in all treatments (Figure 1A,B). Reduced expression of *HaNrf2* or *HaAhR* genes was predicted to significantly reduce the tolerance of *H. armigera* fed on cotton leaves (Figure 1C), or artificial diet mixed with gossypol, 2-tridecanone, chlorantraniliprole and chlorfenapyr (Figure 1D–F). Moreover, the *HaNrf2* and *HaAhR* dsRNA coated cotton leaves enhanced resistance against *H. armigera* (Figure 1G). 

### 2.2. RNA Sequencing and Analysis

To determine transcriptome changes of *HaNrf2-* and *HaAhR*-regulated genes in *H. armigera*, RNA sequencing of nine libraries prepared from dsGFP-, dsNrf2-, and dsAhR-treated larvae was performed. In order to select the xenobiotics-induced genes, RNA sequencing libraries prepared from gossypol, chlorantraniliprole-treated larvae and acetone-treated larvae was performed (Table S3). The transcriptome data of 2-tridecanone-induced larvae were referred to in a previous study (Zhang et al., 2015). A total of 2484 genes were up-regulated and 1991 genes were down-regulated in *HaNrf2* Knockdown *H. armigera* larvae, while 1187 genes were up-regulated and 870 genes were down-regulated in *HaAhR* knockdown *H. armigera* larvae (Figure S1). 

To explore the potential function of down-regulated genes, GO annotation enrichment and KEGG pathway analysis were conducted. GO analysis results showed that most of the down-regulated genes associated with RNAi knockdown of *HaNrf2* or *HaAhR* were classified into ‘cellular component’, ‘biological process’, and ‘molecular function’ processes (Figure S2). Furthermore, the results of KEGG pathway analysis of *HaNrf2* knockdown larvae showed that 437 out of 1991 down-regulated genes were mapped to KEGG orthologous terms in the KEGG pathway database, which were classified into ‘Metabolism of xenobiotics by cytochrome P450’ (16 genes), ‘drug metabolism-cytochrome P450’ (16 genes), and ‘drug metabolism-other enzymes pathways’ (23 genes) categories (Table S4). The results of KEGG pathway analysis of *HaAhR* knockdown larvae showed that 143 out of 870 down-regulated genes mapped to KEGG orthologous terms in the KEGG pathway database, which were classified into ‘Metabolism of xenobiotics by cytochrome P450’ (14 genes), ‘drug metabolism-cytochrome P450’ (13 genes) and ‘drug metabolism-other enzymes pathways’ (16 genes) categories (Table S5).

### 2.3. Analysis of P450s, UGTs and GSTs Genes Were Down-Regulated by RNAi HaNrf2 and HaAhR

**Figure 2:**
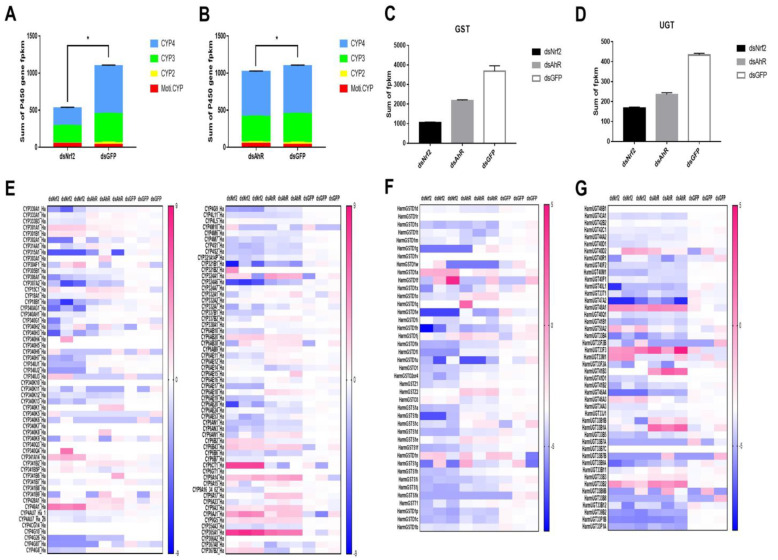
Down-regulation of P450 (**A**,**B**,**E**), GST (**C**,**F**) and UGT (**D**,**G**) detoxification genes putatively regulated by *HaNrf2* and *HaAhR*. Means and SEs from three biological replicates are shown. Asterisks on error bars show significant differences (*p* < 0.05).

The DEGs were selected based on DEGSeq2 analysis using |log2 (fold change)| ≥ 1 and *p* < 0.05 as cut-offs between the dsGFP and dsNrf2 or dsAhR groups, and this criteria was used to determine whether *HaNrf2* and *HaAhR* regulate the expression of detoxification genes in *H. armigera*. Gene expression analysis of all three-phase enzymes (P450s, GSTs, UGTs) is shown in Figure 2. Transcriptome sequencing was used to detect gene expression after RNAi knockdown of *HaNrf2* or *HaAhR*. RNAi knockdown of *HaNrf2* decreased the sum FPKM of P450 genes up to 48% (Figure 2A) and RNAi knockdown of *HaAhR* decreased the sum FPKM of P450 genes up to 90% (Figure 2A,B); most of the CYP3 and CYP4 P450 genes were significantly down-regulated by RNAi knockdown of *HaNrf2* or *HaAhR*. In all, 23 of 113 P450 genes were down-regulated in the *HaAhR* knockdown treatment, and 39 of 113 P450 genes were down-regulated in the *HaNrf2* knockdown treatment (Figure 2E). The same phenomenon was observed for the GST and UGT gene families; RNAi knockdown of *HaNrf2* genes decreased the sum FPKM of GST and UGT genes up to 19% and 38%, respectively; RNAi knockdown of *HaAhR* decreased the expression of GSTs and UGTs by up to 59% and 54%, respectively (Figure 2C,D). Most GST- and UGT-associated genes were down-regulated by RNAi knockdown of *HaNrf2* or *HaAhR*; 25 of 41 GST genes were significantly down-regulated by knockdown of *HaNrf2* and 12 of 41 GST genes were significantly down-regulated by of knockdown *HaAhR* (Figure 2F). A total of 24 of 46 UGT genes were down-regulated by knockdown of *HaNrf2* and 22 of 46 UGT genes were down-regulated by knockdown of *HaAhR* (Figure 2G). 

All the FPKM of detoxification enzyme genes P450, GST, and UGT in each of the libraries is shown in Table S3. According to the genes with *p* < 0.05 and |log2 (fold change)| ≥ 1 were considered to be significantly differently expressed. Most of P450, GST, and UGT genes related to the insecticide metabolism, which were down-regulated by knockdown of *HaNrf2* or *HaAhR* (Figure 2). Interestingly, they also possess a lot of genes related to the synthesis of insect hormone pathways, just as *CYP18A1*, *CYP307A2*, *CYP306A1*, *CYP302A1*, *CYP315A1* and *CYP314A1* are also down-regulated by both knockdown of *HaNrf2* or *HaAhR* (Figure 2).

### 2.4. Transcription Factor Binding Sites Prediction and RT-qPCR Validation of Down-Regulated Genes

**Figure 3:**
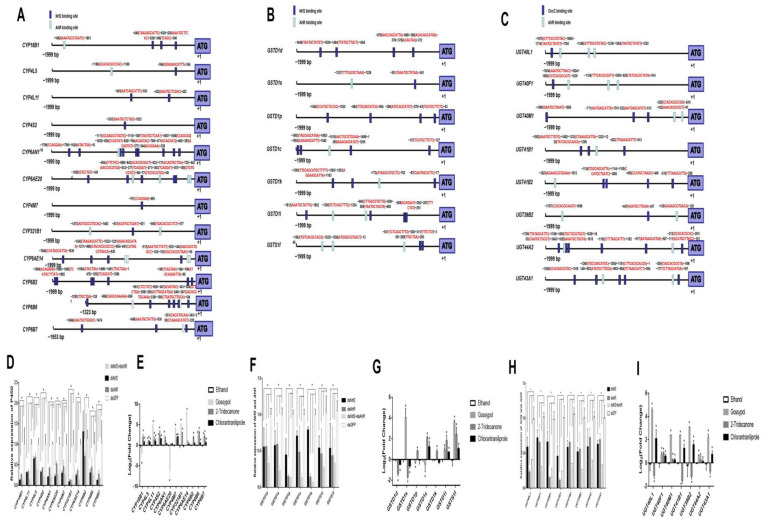
Transcription factor binding sites prediction (**A**–**C**) and RT-qPCR validation (**D**–**I**) of down-regulated P450, GST and UGT genes associated with *HaNrf2* and *HaAhR* knockdown and xenobiotics (gossypol, 2-tridecanone and chlorantraniliprole) treatment. Analysis of the P450, GST and UGT genes’ promoter regions were performed to identify possible regulatory elements. Antioxidant response element (ARE) for Nrf2 (ARE/Nrf2: TMANNRTGAYNNNGCRWWW; DMATGCTKWGT; WCAGHAW), and xenobiotic response element (XRE) from the aryl hydrocarbon receptor (XRE/AhR: TNGCGTG; NCACGCNNN; GAKKTGCGTGASAAGAG; MRGSYTCTTCTCACGCAACTCC; KYKKCTCACGCWRYW; DCACGCAASKCHSAAW; CACGTGCAA). Element position “+1” is defined relative to the start codon ATG. Asterisks on error bars show significant differences (*p* < 0.05).

We used RT-qPCR to validate the down-regulation of P450s, GSTs and UGTs genes associated with RNAi knockdown of *HaNrf2* or *HaAhR* genes, twelve P450 genes (*CYP18B1*, *CYP4L5*, *CYP4L11*, *CYP4S2*, *CYP6AN1*, *CYP6AE14*, *CYP6AE20*, *CYP6B2*, *CYP6B6*, *CYP6B7*, *CYP4M7*, and *CYP321B1*) were selected, including 9 P450 genes which are significantly induced by gossypol, 9 P450 genes which are up-regulated by 2-tridecanone and 7 P450 genes which are up-regulated by chlorantraniliprole, simultaneously down-regulated by RNAi *HaNrf2* and *HaAhR* (Figure 3). The RT-qPCR expression results for these P450 genes were consistent with the transcriptome data; all were down-regulated by knockdown of both *HaNrf2* or *HaAhR* (Figure 3), *CYP18B1*, *CYP4L5*, *CYP4L11*, *CYP4S2*, *CYP6AN1*, *CYP6AE14*, *CYP6AE20*, *CYP6B6*, *CYP6B7*, *CYP4M7*, and *CYP321B1* were decreased up to 12%, 30%, 64%, 32%, 20%, 23%, 21%, 30%, 12%, 29% and 8%, respectively, in *HaNrf2* knockdown *H. armigera*; and *CYP18B1*, *CYP4L5*, *CYP4L11*, *CYP4S2*, *CYP6AN1*, *CYP6AE14*, *CYP6AE20*, *CYP6B2*, *CYP6B6*, *CYP6B7*, *CYP4M7*, and *CYP321B1* were decreased up to 27%, 33%, 68%, 42%, 42%, 35%, 70%, 43%, 27%, 52%, 46% and 12%, respectively, in *HaAhR* knockdown *H. armigera* (Figure 3D). Interestingly, simultaneously knocking down the expression of *HaNrf2* and *HaAhR* will cause the lowest expression of each selected-P450 genes, except *CYP321B1* and *CYP6B2* (Figure 3D). To further clarify the regulatory relationship between *HaNrf2*, *HaAhR*, and P450 genes, 1999 bp fragments upstream of the translation start sites (ATG) of *CYP18B1*, *CYP4L5*, *CYP4L11*, *CYP4S2*, *CYP6AN1*, *CYP6AE14*, *CYP6AE20*, *CYP6B2*, *CYP6B6*, *CYP6B7*, *CYP4M7* and *CYP321B1* genes were selected as potential promoter regions for transcription factor binding site prediction; sequences are shown in Table S6. The prediction results showed that the Nrf2 binding site was found in all selected P450 gene promoters, with each promoter sequence having at least one Nrf2 binding site, and some containing up to nine Nrf2 binding sites in one promoter sequence as was predicted for *CYP6AN1* (Figure 3A). The AhR binding site was found in *CYP18B1*, *CYP4L5*, *CYP6AN1*, *CYP6AE14*, *CYP6AE20*, *CYP6B6*, *CYP6B7* and *CYP321B1* promoters; only *CYP4L11*, *CYP4S2*, *CYP6B2* and *CYP4M7* promoters lacked the AhR binding site (Figure 3A). 

For GST and UGT genes, we validated the effects of *HaNrf2* and *HaAhR* knockdown on the expression of seven GST genes (*GSTD1d*, *GSTD1s*, *GSTD1p*, *GSTD1c*, *GSTD1k*, *GSTD1l*, and *GSTS1f*) and six UGT genes (*UGT40L1*, *UGT40M1*, *UGT41B1*, *UGT39B2*, *UGT44A2* and *UGT43A1*) (Figure 3F,H). RT-qPCR analysis determined that expression for all of these genes was consistent with the transcriptome data (Figure 3F,H). The expression of *GSTD1s*, *GSTD1l*, *GSTS1f*, *UGT40L1*, *UGT40M1*, *UGT41B1*, *UGT39B2*, *UGT44A2* and *UGT43A1* genes was increased by gossypol treatment (Figure 3G,I); *GSTD1c*, *GSTD1k*, *GSTD1l*, *GSTS1f*, *UGT40F1* and *UGT40M1* are up-regulated by 2-tridecanone treatment (Figure 3G,I); *GSTD1c*, *GSTD1l*, *GSTS1f*, *UGT40L1*, *UGT40M1*, *UGT41B1*, *UGT39B2*, *UGT44A2* and *UGT43A1* are induced by chlorantraniliprole treatment (Figure 3G,I). Interestingly, simultaneously knocking down the expression of *HaNrf2* and *HaAhR* will cause the lowest expression of each selected GST genes or UGT genes (Figure 3F,H). As was the case for the P450 genes, 1999 bp fragments upstream of the translation start sites (ATG) of the seven GST genes (*GSTD1d*, *GSTD1s*, *GSTD1p*, *GSTD1c*, *GSTD1k*, *GSTD1l* and *GSTS1f*) and six UGT genes (*UGT40L1*, *UGT40M1*, *UGT41B1*, *UGT39B2*, *UGT44A2* and *UGT43A1*) were selected as potential promoter regions for transcription factor binding site prediction; sequences are shown in Table S6. For GST genes, the promoter sequences were predicted to have at least one Nrf2 binding site, and some contained up to five Nrf2 binding sites in a single promoter sequence as was predicted for *GSTD1d* (Figure 3B,C). The AhR binding site was found in *GSTD1s*, *GSTD1p*, *GSTD1c*, *GSTD1k*, *GSTD1l* and *GSTS1f* gene promoters, only *GSTD1d* was lacking the AhR binding site (Figure 3B). All the six UGT genes promoter sequences were predicted to both have Nrf2 and AhR binding sites (Figure 3C). 

### 2.5. RNAi HaNrf2 and HaAhR Effects on the Synthesis of Insect Hormone

**Figure 4:**
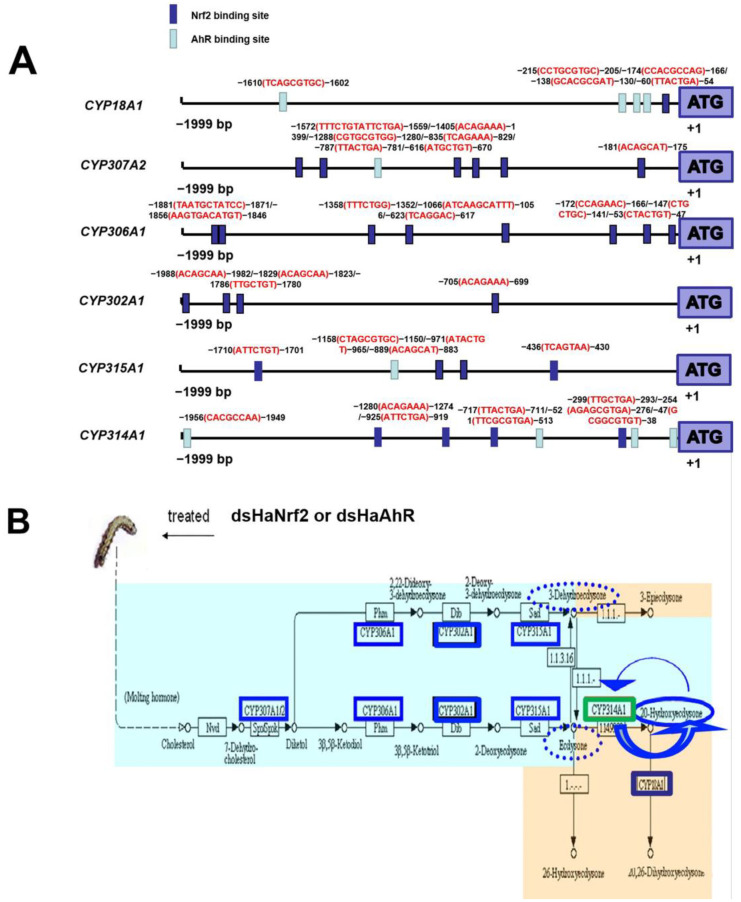
RNAi *HaNrf2* and *HaAhR* affects the biosynthesis and metabolism of insect hormones. (**A**) Analysis of genes’ promoter regions was performed to identify possible regulatory elements. Antioxidant response element (ARE) for Nrf2 (ARE/Nrf2: TMANNRTGAYNNNGCRWWW; DMATGCTKWGT; WCAGHAW), and xenobiotic response element (XRE) from the aryl hydrocarbon receptor (XRE/AhR: TNGCGTG; NCACGCNNN; GAKKTGCGTGASAAGAG; MRGSYTCTTCTCACGCAACTCC; KYKKCTCACGCWRYW; DCACGCAASKCHSAAW; CACGTGCAA). Element position “+1” is defined relative to the start codon ATG. (**B**) RNAi *HaNrf2* and *HaAhR* effects on the genes’ expression in synthesis pathway of insect hormone. Genes both down-regulated following treatment with dsHaNrf2 or dsHaAhR are shown with solid blue lines. Genes only down-regulated following treatment with dsHaNrf2 are shown with solid green lines.

Studies have revealed that *CYP18A1*, *CYP307A2*, *CYP306A1*, *CYP302A1*, *CYP315A1* and *CYP314A1* genes are essential for each of the steps in 20E biosynthesis [20,21,22]. *CYP18A1* belongs to the CYP2 clan and takes part in 20E inactivation, converting 20E to 20, 26-dihydroxyecdysone [23]. The expression of *CYP18A1*, *CYP307A2*, *CYP306A1 CYP302A1*, *CYP315A1* and *CYP314A1* in *H. armigera* are all down-regulated by knockdown *HaNrf2* (Figure 4B), while these genes’ expressions are also down-regulated by knockdown *HaAhR*, except *CYP315A1* (Figure 4B). These genes promoter sequences contain at least one *HaNrf2* or *HaAhR* binding sites (Figure 4A). 

### 2.6. Detoxification Genes Regulated by HaNrf2 and HaAhR in H. armigera

**Figure 5:**
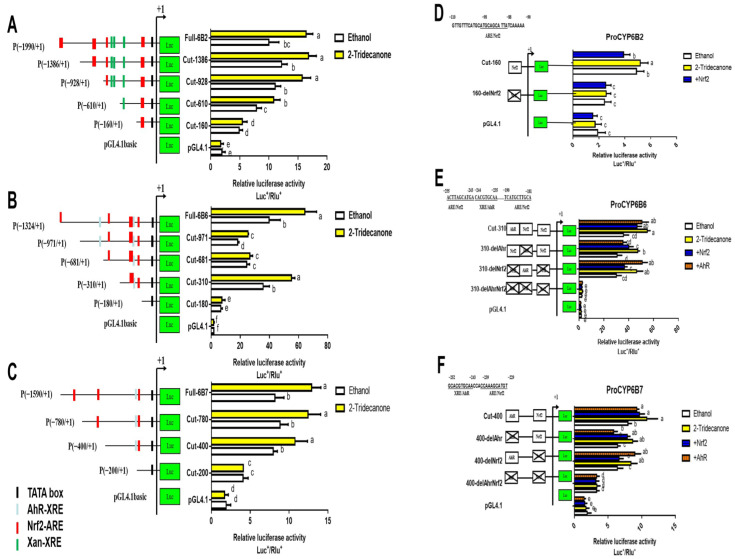
The promoter activity of *CYP6B2* (**A**), *CYP6B6* (**B**) and *CYP6B7* (**C**) induced by 2-tridecanone and the deletion of XRE/AhR or ARE/Nrf2 elements affects 2-tridecanone-induced *CYP6B2* (**D**), *CYP6B6* (**E**) and *CYP6B7* (**F**) promoter activity. Element position “+1” is defined relative to the start codon ATG. Different letters on error bars show significant differences (*p* < 0.05).

2-tridecanone-induced P450s genes (*CYP6B2*, *CYP6B6* and *CYP6B7*) are chosen to confirm the regulation relationship between *HaNrf2* and *HaAhR*. The promoter activity of *CYP6B2*, *CYP6B6* and *CYP6B7* promoter induced by 2-tridecanone and the deletion of XRE/AhR or ARE/Nrf2 elements effects on 2-tridecanone-induced promoter activity are shown in Figure 5. In order to roughly define the regions of *CYP6B2*, *CYP6B6* and *CYP6B7* harboring putative cis elements, a set of *CYP6B2*, *CYP6B6* and *CYP6B7* promoter 5′ progressive deletion constructs were, respectively, co-transfected with the control plasmid of phRL-TK into *H. armigera* fat body cells. Compared to the construct Full-6B2, the progressive 5′ deletions to cut-1386, cut-928, cut-610 and cut-160 were constructed. The 5′ deletion to 610 and 160 significantly decreased the promoter activity (Figure 5A). Compared to the Full-6B2 construct, all the progressive 5′ deletion constructs can be induced by 2-tridecanone, except cut-160 are completely blocked (Figure 5A). Compared to the construct Full-6B6, the progressive 5′ deletions to cut-971, cut-681, cut-310 and cut-180 were constructed. The 5′ deletion to 180 significantly reduced promoter activity, whereas the 5′ deletion to 310 did not significantly decrease the basal promoter activity (Figure 5B). A similar trend was observed for 2-tridecanone-induced promoter activity. Compared to the Full-6B6 construct, all the progressive 5′ deletion constructs decreased the 2-tridecanone-induced activity, except the constructs to 681 and 180 (Figure 5B). For the construct Full-6B7, the progressive 5′ deletions to cut-780, cut-400 and cut-200 were constructed. The 5′ deletion to 200 significantly decreased the promoter activity (Figure 5C). Compared to the Full-6B7 construct, all the progressive 5′ deletion constructs can be induced by 2-tridecanone, except cut-200 are completely blocked (Figure 5C). 

In order to eliminate the interaction effects between ARE/Nrf and XRE/AhR elements completely, we generated a set of internal deletion constructs that deleted, separately, one of the two transcription factor binding sites or deleted both of the sites from the short construct of *CYP6B2*, *CYP6B6* and *CYP6B7* promoters (Figure 5D–F). Both the deleted ARE/Nrf2 element and XRE/AhR element significantly reduced the 2-tridecanone-induced activity and the basal activity of the short construct (Figure 5D–F). Taken together, these findings reveal that the XRE/AhR and ARE/Nrf2 elements are necessary for both the basal and the 2-tridecanone-induced expression of *CYP6B2*, *CYP6B6* and *CYP6B7*. 

**Figure 6:**
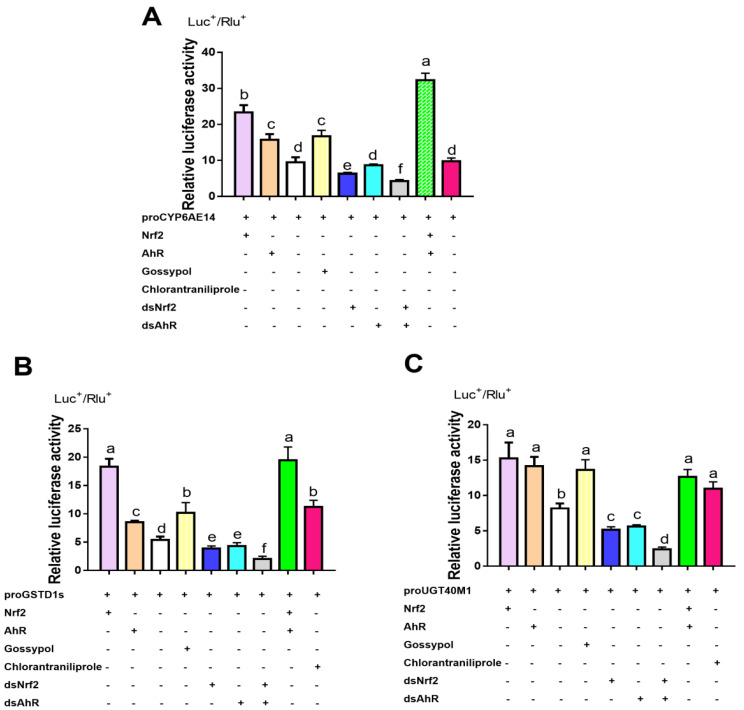
Different xenobiotics (gossypol and chlorantraniliprole) induced the promoter activity of *CYP6AE14* (**A**), *GSTD1s* (**B**) and *UGT40M1* (**C**) genes, which also transiently transfected with dsNrf2, dsAhR, HaAhR or HaNrf2. Different letters on error bars show significant differences (*p* < 0.05).

A dual luciferase assay was used to detect the promoter activity of gossypol and chlorantraniliprole-induced *CYP6AE14*, *GSTD1s*, and *UGT40M1* genes which also transiently transfected with dsNrf2, dsAhR, HaAhR or HaNrf2 (Figure 6). Compared to the control construct, the activity of the *CYP6AE14* promoter was enhanced 2.46- and 1.65-fold, respectively, by transfection with HaNrf2 or HaAhR (Figure 6A); but the activity of the *CYP6AE14* promoter was decreased by 34% and 10%, respectively, by transfection with dsNrf2 or dsAhR (Figure 6A). When co-transfection with dsNrf2 and dsAhR, the promoter activity of *CYP6AE14* was decreased by 55%; but *CYP6AE14* promoter activity was increased 3.40-fold after co-transfection with HaNrf2 and HaAhR (Figure 6A). The promoter activity of *GSTD1s* was significantly enhanced 3.39- and 1.7-fold, respectively, by transfection with HaNrf2 or HaAhR (Figure 6B); and the promoter activity of *UGT40M1s* were significantly enhanced 1.86-and 1.73-fold, respectively, by transfection with HaNrf2 or HaAhR (Figure 6C). The activity of the *GSTD1s* promoter was decreased 34% and 10%, respectively, by transfection with dsNrf2 or dsAhR (Figure 6B), and the promoter activity of the *UGT40M1* was decreased 34% and 10%, respectively, by transfection with dsNrf2 or dsAhR (Figure 6C). When co-transfection with dsNrf2 and dsAhR, the promoter activity of *GSTD1s* and *UGT40M1* was decreased by 63% and 71%, respectively; but *GSTD1s* and *UGT40M1* promoter activity was increased 3.60-fold and 1.54-fold after co-transfection with HaNrf2 and HaAhR (Figure 6B,C). The promoter activity of *CYP6AE14*, *GSTD1s*, and *UGT40M1s* was significantly induced by gossypol and chlorantraniliprole treatment (Figure 6). These results confirm that both the HaNrf2 and HaAhR signal pathways play a significant role in the activation of *CYP6AE14*, *GSTD1s*, and *UGT40M1* genes’ expression. 

**Figure 7:**
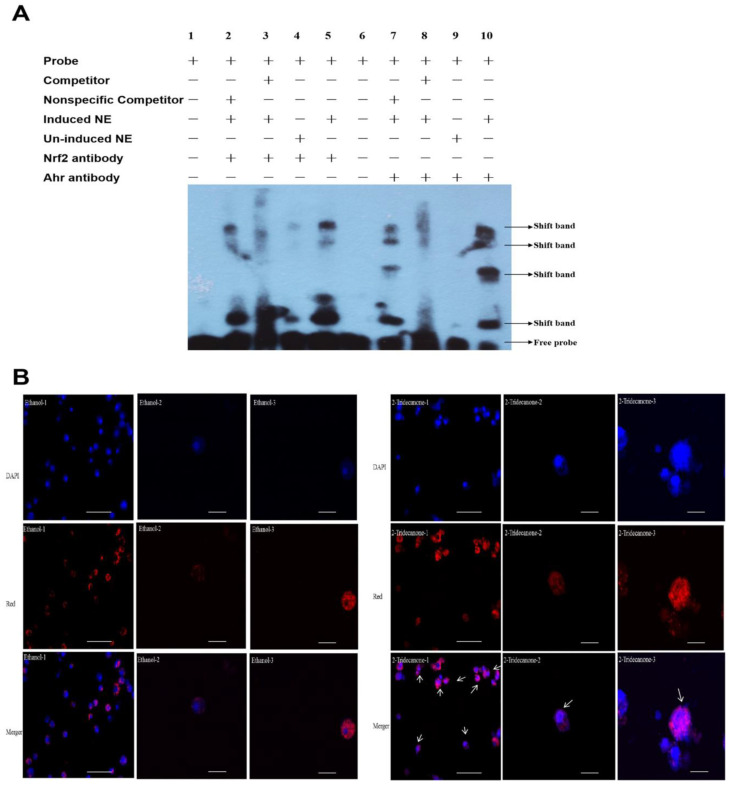
(**A**) sEMSA analysis of the expression of HaNrf2 and HaAhR in nuclear cells after being treated by 2-tridecanone. Nuclear extracts (NE) were prepared from un-induced (un-induced NE) or 2-tridecanone-induced (induced NE) (125 μM) homologous *H. armigera* fat body cells. Lane 1, only Nrf2 probe, no nuclear extracts; Lane 2, Nrf2 probe and Nrf2 antibody with nuclear extracts from induced cells, in competition with unrelated nonspecific sequence competitors (200-fold greater than that of the labeled probe); Lane 3, Nrf2 probe and Nrf2 antibody with nuclear extracts from induced cells, in competition with unlabeled probe (concentration 200-fold greater than that of the labeled probe); Lane 4, Nrf2 probe and Nrf2 antibody with nuclear extracts from un-induced cells; Lane 5, Nrf2 probe and Nrf2 antibody with nuclear extracts from 2-tridecanone-induced cells; Lane 6, only AhR probe, no nuclear extracts; Lane 7, AhR probe and AhR antibody with nuclear extracts from 2-tridecanone-induced cells, in competition with unrelated nonspecific sequence competitors (200-fold greater than that of the labeled probe); Lane 8, AhR probe and AhR antibody with nuclear extracts from induced cells, in competition with unlabeled probe (concentration 200-fold greater than that of the labeled probe); Lane 9, AhR probe and AhR antibody with nuclear extracts from un-induced cells; Lane 10, AhR probe and AhR antibody with nuclear extracts from induced cells. (**B**) 2-Tridecanone induced HaNrf2 transfer into the nucleus. DAPI: DAPI staining the nucleus region; Red: using recombinant pAC-v5-Red-Nrf2 plasmid to show the localization of Nrf2; Merger: subcellular localization of Nrf2; 2-tridecanone: the subcellular localization of Nrf2 when the fat body cells were treated with 125 μM 2-tridecanone, Ethanol: the subcellular localization of Nrf2 when the fat body cells were treated with ethanol. Scale bar in 2-tridecanone-1, Ethanol-1: 100 μm; Scale bar in Ethanol-2, Ethanol-3, 2-Tridecanone-2, 2-Tridecanone-3: 20 μm.

To determine whether the essential elements XRE/AhR and ARE/Nrf2 are recognized and specifically bound by proteins from nuclear extracts prepared from un-induced (ethanol-treated) and/or 2-tridecanone-induced *H. armigera* fat body cells, we conducted sEMSA (Figure 7A). We used biotin-labeled double-stranded oligos containing the XRE/AhR element or the ARE/Nrf2 element in *CYP6B6* promoters as the probe. As the competitors, we used unlabeled XRE/AhR or ARE/Nrf2 (i.e., cold probe) and an unlabeled unrelated nonspecific sequence. In the absence of competitors, both the un-induced (Lane 4, Lane 9 in Figure 7A) and the 2-tridecanone-induced (Lane 2–3, 5 and Lane 7–8, 10 in Figure 7A) nuclear extracts formed two sequence specific DNA-protein complexes with the ARE/Nrf2 (Lane 2–5 in Figure 7A) and XRE/AhR (Lane 7–10 in Figure 7A) biotin probes. These complexes were completely out-competed by an excess of the XRE/AhR and ARE/Nrf2 cold competitor probes (Lane 3 and 8 in Figure 7A). The DNA-protein complexes with 2-tridecanone-induced nuclear extracts and HaNrf2 specific antibody displayed a stronger signal than those with un-induced nuclear extracts and HaNrf2-specific antibody; the DNA-protein complexes with 2-tridecanone-induced nuclear extracts and HaAhR-specific antibody displayed a stronger signal than those with un-induced nuclear extracts and HaAhR-specific antibody (Figure 7A). 

After *H. armigera* fat body cells were transfected with the pAcV5His-Red-HaNrf2 construct for 24 h, the subcellular localization of HaNrf2 was observed. We found that HaNrf2 was mainly located in the cytoplasm with the red florescence (Figure 7B). These fat body cells were then treated with 2-tridecanone in a final concentration of 125 μM for 4 h. We observed that the subcellular localization of Nrf2 was mainly in the nucleus following 2-tridecanone treatment (Figure 7B), while the control cells treated with ethanol still had signals indicating that Nrf2 was localized to the cytoplasm (Figure 7B). 

**Figure 8:**
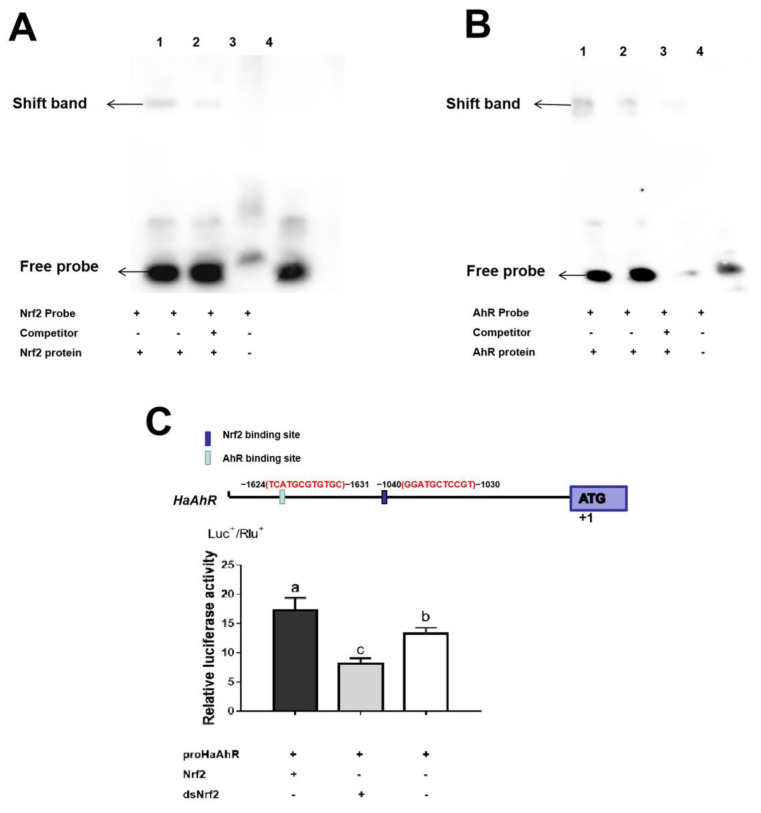
The crosstalk between HaNrf2 and HaAhR regulates the P450s, UGTs and GSTs genes in *H. armigera*. (**A**) EMSA method to verify the HaNrf2 binding sits in the promoter of *HaAhR*; (**B**) EMSA method to verify the HaAhR binding sits in the promoter of *HaAhR*; (**C**) overexpressed HaNrf2 and HaAhR effects on the promoter activity of *HaAhR*. Element position “+1” is defined relative to the start codon ATG. Different letters on error bars show significant differences (*p* < 0.05).

The 1999 bp fragments upstream of the translation start sites (ATG) of *HaAhR* gene were selected as potential promoter regions for transcription factor binding site prediction; the prediction results showed that the Nrf2 and AhR binding sites were found in *HaAhR* gene promoters (Figure 8C). When co-transfection with HaNrf2, the promoter activity of *HaAhR* was enhanced 1.4-fold; but the *HaAhR* promoter activity was decreased about 60% after co-transfection with dsNrf2 (Figure 8C). The EMSA method was used to verify the transcription factor HaNrf2 and HaAhR binding sites, a single-target shift band can be clearly detected in each treatment (Figure 8A,B).

**Figure 9:**
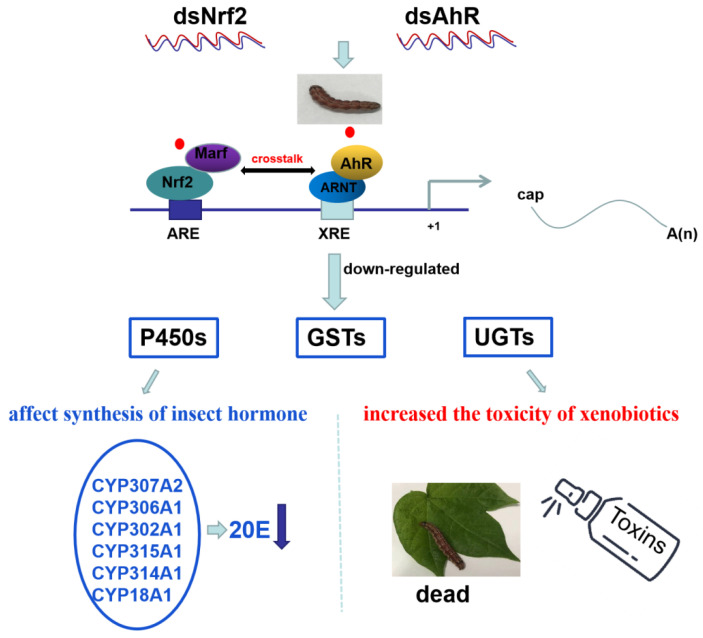
A schematic illustration of the proposed model of how the crosstalk between HaNrf2 and HaAhR regulate the P450s, GSTs and UGTs genes and mediate the susceptibility of xenobiotics in *H. armigera*. Xenobiotics are represented by a red dot.

Based on these results, a schematic illustration of the proposed model of how the crosstalk between HaNrf2 and HaAhR regulate the P450s, GSTs and UGTs and mediate the susceptibility of xenobiotics (gossypol, 2-tridecanone and chlorantraniliprole) in *H. armigera* is presented in Figure 9. 

## 3. Discussion

Analysis of insect genome sequences has revealed that expansion of the P450, GST, and UGT gene families is related to plant allelochemicals and insecticides resistance [24,25]. Xenobiotic transcription factors play important roles in the regulation of detoxification genes involved in xenobiotic metabolism [8]. It was also reported that Nrf2 and AhR transcription factors are involved in the regulation of some three-phase enzyme gene members responsible for insecticide resistance [13,26,27]. 

Several studies in insects have shown that some three-phase enzymes genes, such as some members of P450s, GSTs, UGTs, and ABC transporter genes regulated by Nrf2, play important roles in insecticide resistance [11,26,28,29,30,31]. For example, in *Tribolium castaneum*, Nrf2 regulates genes involved in insecticide detoxification such as *CYP4G7*, *CYP4G14*, *GST1*, *ABCAUB*, *ABCAA1*, *ABCAA1L*, and *ABCA9B* [26]. Hu et al. (2020) showed that Nrf2 regulates the expression of *CYP321A16* and *CYP332A1*, which mediate chlorpyrifos resistance in *Spodoptera exigua* [11]. Moreover, in *Spodoptera litura*, Chen et al. (2018), Lu et al. (2020) also found that Nrf2 regulates *GSTe1* and *CYP6AB12* expression in response to insecticide [28,29]. Shi et al. (2021) reported that transcription factor Nrf2 regulates the expression of some detoxification genes that mediate indoxacarb resistance in *Spodoptera litura* [30]. In *Bombyx mori*, transcription levels of *CYP302A1* and *CYP306A1* were significantly decreased in cells after treatment with *BmNrf2* dsRNA, whereas their transcription levels were significantly increased (2.15- and 1.31-fold, respectively) after treatment with the Nrf2 agonist curcumin [31]. In a study in *Drosophila melanogaster*, *CYP6DA1* contained Nrf2 binding sites in the 5′-promoter core region, and changes in these binding sites were associated with altered promoter activity of *CYP6DA1* as well as resistance to DDT [32]. In *Helicoverpa zea*, induction of the phase I gene *CYP321A1* by flavone compounds has been shown to be mediated by Nrf2 [33]. 

In insects, an XRE element of AhR has been functionally characterized from the promoters of *CYP6B1* and *CYP6B4* genes; this element is associated with the xanthotoxin cascade [34]. *CYP6B4* and *CYP6B1* promoters contain putative XRE-AhR elements identical to the aryl hydrocarbon response elements present in mammalian phase I detoxification genes. Transfection of *CYP6B4* and *CYP6B1* promoters containing XRE-AhR elements were induced significantly by benzo(α)pyrene, an aryl hydrocarbon, as well as by xanthotoxin, an allelochemical encountered in host plants [35]. Mutational analysis of the *CYP6B1* promoter indicated that the XRE-AhR element is necessary for both constitutive and xanthotoxin-inducible induction of the *CYP6B1* promoter [36]. The aryl hydrocarbon receptor (AhR) regulates *CYP6DA2* in *Aphis gossypii*, conferring gossypol and spirotetramat tolerance [37]. AhR also controls the over expression of P450 genes in *D. melanogaster* that are resistant to insecticides [6]. 

Despite some excellent studies on the single detoxification gene member induced by host plant allelochemicals or insecticides regulated by Nrf2 or AhR, the network of the detoxifications genes which can be regulated by Nrf2 and AhR is still unknown in insects. There had also been no reports about the crosstalk between AhR and Nrf2 which can regulate the expression of detoxification genes in insects. The study of insect adaptation to the defensive metabolites of host plants and various kinds of insecticides in order to acquire resistance is a hot topic in the field of controlling pests, however, the mechanism of insect adaptation to these xenobiotics is still unclear. Our results found that Nrf2 and AhR signaling pathways in *H. armigara* are not only regulated in the form of one detoxification gene member to one kind of xenobiotic, but are also regulated by multiple detoxification genes (including genes related to the synthesis of insect hormone and xenobiotics resistance.) It should be a general signaling pathway for revealing the adaptive mechanism of insects to xenobiotics. 

This study comprehensively elucidated the effect of *HaNrf2* and *HaAhR* knockdown on the detoxification expression in *H. armigera*. We found that knocking down the expression of *HaNrf2* or *HaAhR* by RNAi can significantly increase the sensitivity of *H. armigera* to gossypol, 2-tridecanone, chlorantraniliprole and chlorfenapyr (Figure 1). To investigate the function of HaNrf2 and HaAhR in regulating the sensitivity of plant allelochemicals and insecticides in *H. armigara*, full-length sequences of the *H. armigera Nrf2* and *AhR* genes were cloned and used for designing dsRNAs specific to each gene. dsHaNrf2 and dsHaAhR treatment enhanced cotton leaf defenses against *H. armigera* feeding, and the reduced expression of *HaNrf2* and *HaAhR* both significantly increase the gossypol, 2-tridecanone, chlorantraniliprole and chlorfenapyr susceptibility of *H. armigera* larvae fed on dsRNA-coated cotton leaves or an artificial diet mixed with xenobiotics (Figure 1). Some studies also reported that silencing of detoxification enzyme genes associated with plant allelochemical tolerance or insecticide resistance in insects can result in their increased susceptibility to plant allelochemicals or insecticides [5,19,30]. In this study, our results revealed that HaNrf2 and HaAhR are both key regulatory factors involved in mediating *H. armigera* susceptibility to xenobiotics. 

To comprehensively elucidate the genes regulated by HaNrf2 and HaAhR in *H. armigera*, we performed RNA sequencing on *H. armigera* larvae that were treated with *HaNrf2*, *HaAhR*, or *GFP* dsRNA. Differential gene expression analysis showed that 2484 genes were up-regulated and 1991 genes were down-regulated by RNAi knockdown of *HaNrf2*, and 1187 genes were up-regulated and 870 genes were down-regulated by RNAi knockdown of *HaAhR* (Figure S1). Based on the expected pattern of positive regulation between HaNrf2 or HaAhR and their target genes, we mainly focused on the down-regulated genes which included a lot of three-phase detoxification enzyme genes. Interestingly, we found *HaNrf2* knockdown decreased the sum FPKM of P450 genes by up to 48% (Figure 2A), and *HaAhR* knockdown decreased the sum FPKM of P450 genes by up to 90% (Figure 2B). In all, 23 of 113 P450 genes in *H. armigera* were down-regulated by *HaAhR* knockdown, and 39 of 113 P450 genes were down-regulated by *HaNrf2* knockdown (Figure 2E). The same phenomenon was observed for the GST and UGT gene families; knockdown of *HaNrf2* or *HaAhR* decreased the sum FPKM of GST and UGT genes (Figure 2F,G). 

Previous studies have found that the expression of P450 genes, UGT genes, and GST genes can be significantly induced by gossypol, 2-tridecanone, chlorantraniliprole or chlorfenapyr treatments [17,18]. P450s are important components of Phase I detoxification enzymes. Our previous study showed that multiple over-expressed detoxification genes play important roles in *H. armigera* 2-tridecanone tolerance [17], with 2-tridecanone inducing the highest cluster genes (*CYP6B2*, *CYP6B6* and *CYP6B7*) also being regulated by *HaNrf2* and *HaAhR* (Figure 5). The promoter activity of *CYP6B2*, *CYP6B6* and *CYP6B7* promoter induced by 2-tridecanone and the deletion of XRE/AhR or ARE/Nrf2 elements’ effects on 2-tridecanone-induced promoter activity are shown in Figure 5. In order to roughly define the regions of *CYP6B2*, *CYP6B6* and *CYP6B7* harboring putative cis elements, a set of *CYP6B2*, *CYP6B6* and *CYP6B7* promoter 5′ progressive and ARE/Nrf and XRE/AhR elements deletion constructs were, respectively, co-transfected with the control plasmid of phRL-TK into *H. armigera* fat body cells. We found that the essential cis elements required for both basal and 2-tridecanone-induced expression of *CYP6B2*, *CYP6B6* and *CYP6B7* are located in the region containing proximal AhR and Nrf2 transcription factor binding sites (Figure 5). Then, we used confucus to observe that the subcellular localization of HaNrf2 was mainly localized in the nucleus following 2-tridecanone treatment (Figure 7B), while the control cells still had no signals indicating that HaNrf2 was transferred from cytoplasm to nucleus, which implies that 2-tridecanone requires more HaNrf2 to actively enter in nucleus (Figure 7B). The above results mean that 2-tridecanone-induced *CYP6B2*, *CYP6B6* and *CYP6B7* genes’ overexpression is regulated by both HaNrf and HaAhR pathways.

*CYP6AE14* was an especially promising candidate as one P450 gene involved in cotton gossypol detoxification by *H. armigera* [38]. When *CYP6AE14* was silenced in *H. armigera* via feeding of dsRNA or transgenic cotton transformed with a construct expressing dsRNA, larval growth was greatly reduced in the presence of gossypol [38]. *CYP6AE14* genes are induced by gossypol treatment in *H. armigera*; it also can by down-regulated by RNAi *HaNrf2* or *HaAhR* (Figure 2). Accordingly, we suggest that treatment with dsHaNrf2- and dsHaAhR-enhanced cotton leaf defenses against *H. armigera* and knocking down *HaNrf2* and *HaAhR* can affect the expression of multiple detoxification enzyme genes associated with gossypol tolerance. To this end, we used a dual luciferase assay to detect the promoter activity of *CYP6AE14*, *GSTD1s*, and *UGT40M1* genes in *H. armigera* larvae transfected with HaAhR or HaNrf2. Compared to the control construct, the activities of the *CYP6AE14*, *GSTD1s*, and *UGT40M1* promoters were significantly enhanced by co-transfection with HaNrf2 or HaAhR (Figure 6). These results confirm that both Nrf2 and AhR transcription factors play a significant role in the activation of downstream expression in genes belonging to the P450, GST, and UGT gene families. The promoter activity of *CYP6AE14*, *GSTD1s*, and *UGT40M1s* was significantly induced by gossypol and chlorantraniliprole treatment (Figure 6). All above results confirm that both HaNrf2 and HaAhR signaling pathways play a significant role in the activation of *CYP6AE14*, *GSTD1s*, and *UGT40M1* genes’ expression treated by gossypol or chlorantraniliprole. *H. armigera* P450s in the CYP6 subfamily were down-regulated by RNAi *HaNrf2* or *HaAhR* (Figure 2), which were also shown to be involved in the tolerance of xanthotoxin, gossypol acetate, 2-tridecanone, nicotine, and coumarin [6,39], and P450-associated increases in detoxification were also shown to be the primary cause of high insecticide resistance in *H. armigera* [40].

*CYP18A1*, *CYP307A2*, *CYP306A1*, *CYP302A1*, *CYP315A1* and *CYP314A1* genes are related to the synthesis of insect hormones 20E. 20E is a polyhydroxylated steroid hormone that controls molting and thereby affects the growth of arthropods. Studies using insects have revealed that the Halloween P450 genes (*CYP18A1*, *CYP307A2*, *CYP306A1*, *CYP302A1*, *CYP315A1* and *CYP314A1*) are essential for each of the steps in 20E biosynthesis [20,21,22]. *CYP18A1* belongs to the CYP2 clan and takes part in 20E inactivation, converting 20E to 20, 26-dihydroxyecdysone [23]. The expression of *CYP18A1*, *CYP307A2*, *CYP306A1*, *CYP302A1*, *CYP315A1* and *CYP314A1* in *H. armigera* are all down-regulated by knockdown *HaNrf2* (Figure 4B), while these genes’ expressions are also down-regulated by knockdown *HaAhR*, except *CYP315A1* (Figure 4B). These genes’ promoter sequences at least contain one HaNrf2 or HaAhR binding site (Figure 4A). Previous studies found that a lot of plant allelochemicals or insecticides, similar to gossypol, 2-tridecanone, chlorantraniliprole will not only decrease the growth rate of insects, but can also decrease the susceptibility to these xenobiotics [1,17,18]. Our results provide a general signaling pathway for revealing the adaptive mechanism of insects to xenobiotic, and explain why many xenobiotic will not only decrease the growth rate of insects, but also can decrease their capability to metabolize xenobiotics. Our results prove that a pathway may exist in HaNrf2 and HaAhR to regulate the P450 genes which are related to insect hormones synthesis.

The EMSA method was used to verify transcription factor HaNrf2 and HaAhR binding sites in *HaAhR* promoter sequence, showing a single-target shift band can be clearly detected in each treatment (Figure 8A,B). These results confirm that there is crosstalk between HaNrf2 and HaAhR transcription factors that plays a significant role in the activation of downstream detoxification genes’ expression. In mammals, AhR has been reported to affect the Nrf2 expression [41,42]. There may be direct crosstalk between the Nrf2 and the AhR pathways for the induction of phase II genes (*NQO1*, *GSTA2* and *UGT1A6*) in mammals [41]. Some researchers also demonstrated that Nrf2 gene transcription is directly modulated by AhR activation in mammals [42]. However, there have been no reports about the crosstalk between Nrf2 and AhR signaling pathways in insects. Our research reported that expression of AhR and Nrf2 are partially dependent on each other, implying that Nrf2 modulates both transcription of AhR and its downstream targets in insects. Our results confirm that there is crosstalk between the HaNrf2 and HaAhR signaling pathways to regulate the expression of detoxification genes in *H. armigera*.

The RNAi method has been widely recognized as a novel and safe strategy in pest management due to its high sequence-dependent specificity and simplicity to degrade. Therefore, HaNrf2 and HaAhR could serve as potential targets of dsRNA spraying for pest management.

## 4. Materials and Methods

### 4.1. Insects

The cotton bollworm population used in this study was collected from Handan in Hebei Province, China in 1998, and reared on an artificial diet in an air-conditioned room maintained at 26 ± 1 °C, 70–80% relative humidity, and under a photoperiod of 16:8 h (L:D). The artificial diet was supplied as described in our previous study [19]. Adults were held under the same conditions and supplied with a 10% sugar solution.

### 4.2. RNA Isolation, Reverse Transcription, and Gene Cloning

Total RNA was isolated from *H. armigera* larvae using TRIzol^®^ reagent (Invitrogen, Carlsbad, CA, USA). One µg of RNA sample was used as the template for synthesis of first-strand cDNA, using the cDNA synthesis kit (Takara, Japan). Specific primers of *HaNrf2* (KU355787.1) and *HaAhR* (XM_021331544.1) were designed and synthesized to amplify the complete open reading frame region (Table S1).

### 4.3. HaNrf2 and HaAhR RNAi

Based on *HaNrf2* and *HaAhR* sequences, we used online prediction software E-RNAi (http://www.dkfz.de/signaling/e-rnai3/) (accessed on 1 January 2021) [43] to predict putative RNAi sites and designed specific primers using DNAMAN 6.0 software. RNAi method is according to our previous study [19], HaNrf2, HaAhR, and green fluorescence protein (GFP) genes fragments for in vitro transcription reactions were amplified by PCR using primers that contain the T7 promoter sequence at the 5′ end (Table S1). The products were used as templates to synthesize dsRNAs using the MEGAscript T7 transcription kit (Invitrogen, USA). The dsRNA product sizes (412 bp for dsHaNrf2; 438 bp for dsHaAhR; 288 bp for dsHaGFP) were analyzed by 1% agarose gel electrophoresis and quantified using a spectrophotometer. The 2nd instar larvae, after being starved for 12 h, were offered an artificial diet containing *HaAhR* or *HaNrf2* dsRNA (15 μg/g, *w*/*w*) for 24 h; *GFP* dsRNA (15 μg/g, *w*/*w*) was used as a negative control. Thirty larvae were used for each treatment, and three replications were performed. The dsRNA-mediated depletion of *HaNrf2* or *HaAhR* transcripts was analyzed by RT-qPCR using the primers listed in Table S1. Each sample was analyzed in triplicate and normalized to the internal control, *EF-1α* [19]. Statistical analyses were performed using GraphPad Prism 5.0 software (GraphPad Prism, San Diego, USA), and a *p* value < 0.05 was considered statistically significant.

### 4.4. Xenobiotics Treatment following HaNrf2 or HaAhR Knockdown

To determine the effects of *HaNrf2* and *HaAhR* knockdown on the efficacy of the plant allelochemicals and insecticides against *H. armigera*, gossypol was dissolved in acetone to concentration of 0.02 mg/g, (*w:w*) then mixed with 1 g of artificial diet and 15 μg/g (*w:w*) of dsNrf2, dsAhR, or dsGFP; 2-tridecanone was dissolved in ethanol to concentration of 0.05 mg/g (*w:w*) then mixed with 1 g of artificial diet and 15 μg/g (*w:w*) of dsNrf2, dsAhR, or dsGFP; chlorantraniliprole was dissolved in N, N-dimethylformamide to concentration of 0.05 μg/g (*w:w*) then mixed with 1 g of artificial diet and 15 μg/g (*w:w*) of dsNrf2, dsAhR, or dsGFP; chlorfenapyr was dissolved in acetone to concentration of 0.1 μg/g (*w:w*) then mixed with 1 g of artificial diet and 15 μg/g (*w:w*) of dsNrf2, dsAhR, or dsGFP; The solvents were used as the negative control; dsGFP combined with different pesticides are used as the negative control. Fifty 1nd instar larvae were used for each treatment, and the experiments were repeated in triplicate. Cotton leaf discs (20 mm diameter) were cut using a stainless-steel cork-borer, placed in cell culture plates filled with 1 mL of 2% agar to maintain leaf humidity, and coated with 1 μg of dsRNA per leaf. Ten 1nd instar larvae were used for each treatment, and the experiments were repeated in triplicate. Mortality of larvae reared at 25 ± 2 °C, 65% ± 5% RH, and under a 14:10 h (L:D) photoperiod was recorded after each collection time point. Larvae were considered dead if they failed to make a coordinated movement when prodded with a brush. Mean and standard errors were obtained from at least three independent replications. Statistical analyses were performed using GraphPad Prism 5.0 software, and a *p* value < 0.05 was considered statistically significant.

### 4.5. RNA Sequencing

Total RNA isolated from dsNrf2-, dsAhR- and dsGFP-treated 2nd instar *H. armigera* larvae for 24 were used to prepare RNA sequencing libraries using the NEBNext^®^ UltraTM RNA Library Prep Kit for Illumina^®^ (NEB, Beverly, MA, USA) following the manufacturer’s recommendations. Thirty larvae were used for each treatment, and the experiments were repeated in triplicate. The library preparations were sequenced on an Illumina Hiseq 2500 platform at the Novogene Bioinformatics Institute (Beijing, China). Then, 2 mg/g gossypol or 2μg/g chlorantraniliprole-treated six instar *H. armigera* larvae for 24 h and acetone-alone-treated larvae were used to prepare RNA sequencing. The transcriptome data of 2-tridecanone-induced larvae were referred to in our previous study [17].

Gene expression levels were estimated by the number of Fragments Per Kilobase of transcript sequence per Million base pairs sequenced (FPKM) [44]. Differentially expressed gene analysis was performed using the DESeq2 R package 1.16.1 [45]. Genes with *p* < 0.05 and |log2 (fold change)| ≥ 1 were considered differentially expressed. Gene Ontology (GO) enrichment analysis (*p* < 0.05) was used to identify functional modules of down-regulated genes [46]. To further characterize the down-regulated genes, we performed pathway analysis using the Kyoto Encyclopedia of Genes and Genomes (KEGG) pathway database (http://www.genome.jp/kegg/) (accessed on 1 January 2021).

### 4.6. Quantitative PCR Analysis of Down-Regulated Genes and Prediction of Transcription Factor Binding Sites

Reverse transcription-quantitative PCR (RT-qPCR) was performed to verify the results of the RNA-seq data. Nine detoxification genes from the down-regulated group were selected for expression analysis by RT-qPCR, and EF-1a was used as the reference gene [19]. The primer sequences used in the RT-qPCR analyses are given in Table S1. The relative gene expression levels were calculated using the 2^−∆∆CT^ method [47]. The RT-qPCR data was expressed as the mean ± SD of three independent biological replicates. Promoter sequence information of the down-regulated detoxification genes was acquired from the *H. armigera* genome [48]. Transcription factor binding sites in the promoters were predicted using JASPAR [49].

### 4.7. Transient Transfection and Dual Luciferase Assay

The homologous *H. armigera* fat body cell line was generously provided by Dr. Huan Zhang (Zoology, CAS, China). The cell line was routinely maintained with sf900-III insect serum-free medium (Thermo Fisher Scientific, USA) supplemented with 10% heat-inactivated fetal bovine serum (Gibco, USA) at 37 °C. The full-length coding regions of *HaNrf2* and *HaAhR* were constructed into pAcV5His vector [13]. The *H. armigera* fat body cells were seeded into 96-well plates (1 × 10^4^ cells/ well) then transiently co-transfected with different gene promoters fused to pGL4 luciferase reporter constructs (1 mg/well) combined with pAcV5His-Red-HaNrf2 (0.5 mg/well), pAcV5His-GFP-HaAhR (0.5 mg/well), dsNrf2 (4 μg/well) or dsAhR (4 μg/well). The internal renilla luciferase plasmid phRL-TK (Promega; 0.2 mg/well) was used as the control reporter plasmid. Cellfectin-II reagent (Invitrogen; 1 μL per well) was used to transfect all of the plasmids into fat body cells. After 24 h, the cells were harvested and lysed, and lysates were used to measure renilla and firefly luciferase activities with a Dual-Luciferase Reporter Assay System (Promega, Wisconsin, USA) on a GloMax^®^ 96 Microplate Luminometer (Promega, Madison, WI, USA). The relative firefly luciferase activity, normalized against the renilla luciferase activity, reported for each construct was used to calculate the mean and standard error of four independent transfections of one representative sample. Statistical analyses were performed using GraphPad Prism version 5.0 software (GraphPad Prism, San Diego, CA, USA), and *p* values < 0.05 were considered statistically significant.

### 4.8. Super Electrophoretic Mobility Shift Assays (sEMSA)

*H. armigera* fat body cells seeded into wells of 6-well plates (1 × 10^6^ cells/well) were treated with 2-tridecanone at a final concentration of 125 μM or an equal volume of ethanol (control). After 24 h, the cells were harvested and used to prepare control and 2-tridecanone-induced *H. armigera* fat body cell nuclear extracts according to Lahiri and Ge (2000) [50]. Biotin end-labeled double-stranded oligonucleotides (Biotin Nrf2: 5′biotin-taacaacTCATGCTTGCAGcttttcta-3′biotin; the fragment targeted the region between −152 to −162 of the *CYP6B6* promoter; biotin AhR: 5′biotin-tcacCATGACACGTGCAAacgc-3′biotin the fragment targeted the region in the *CYP6B6* promote) containing the essential Nrf2 or AhR binding site (the capital letters in the sequence) were used as the probe for sEMSA. DNA-nuclear extract binding reactions were carried out in a 20 µL volume containing 0.17 µg of *H. armigera* fat body cell nuclear extracts, 2 µL of 10× binding buffer (10 mM Tris, pH 7.5, 50 mM KCl, 5 mM MgCl_2_, 1 mM dithiothreitol, 0.05% Nonidet P-40, and 2.5% glycerol), 1 µL of poly (dI-dC) (1 µg/µL), 80 fmol of the biotin-labeled probe, and 0.5 µL of the Nrf2 or AhR polyclonal antibody. We used pET28a vector to express the fusion proteins of Nrf2 and AhR, and then used these fusion proteins to prepare polyclonal antibodies against Nrf2 and AhR in male New Zealand rabbits. Prepared and purified polyclonal anti-Nrf2 and anti-AhR antibodies were stored at −70 °C. The reaction mixture was preincubated for 10 min at 25 °C, after which the probe was added. Incubation continued for another 20 min at 25 °C. Competition reactions were performed in the same conditions, except for the addition of a 200-fold molar excess of unlabeled competitor oligonucleotides (Nrf2: 5′-taacaacTCATGCTTGCAGcttttcta-3′, the fragment was between −152 to −162 of *CYP6B6* promoter; AhR: 5′-tcacCATGACACGTGCAAacgc-3′, the fragment was located in *CYP6B6* promoter, each fragment contained 4 or 5 flanking nucleotides at both ends.

Biotin end-labeled double-stranded oligonucleotides (Biotin Nrf2: 5′biotin-aaATGCTtatt-3′; Biotin AhR: 5′biotin-gaacGCGTGgcc-3′) containing the essential Nrf2 or AhR binding site in *HaAhR* promoter sequences (the capital letters in the sequence) were also used as the probe for sEMSA. The reaction mixture including the fusion protein HaNrf2 or HaAhR was preincubated for 10 min at 25 °C, after which the probe was added. Incubation continued for another 20 min at 25 °C. Competition reactions were performed in the same conditions, except for the addition of a 200-fold molar excess of unlabeled competitor oligonucleotides (Biotin Nrf2: 5′biotin-ggATGCTccgt-3′; Biotin AhR: 5′biotin-tcatGCGTGtgc-3′, each fragment contained 4 or 5 flanking nucleotides at both ends. All the reaction mixtures were electrophoresed through a non-denaturing polyacrylamide gradient gel (Bio-Rad, Hercules, CA, USA) for 2.5 h in 0.5 TBE buffer at 80 volts at 4 °C, and then electro-transferred onto a Hybond-N nylon membrane (Amersham Biosciences, Uppsala, Sweden) for 30 min at 220 mA at 4 °C. The retarded DNA-nuclear protein complexes on the membrane were fixed by UV cross-linking and visualized with a LightShift Chemiluminescent Electrophoretic Mobility Shift Assay Kit (Thermo Fisher Scientific, Waltham, USA). The intensities of the bands formed were assessed via X-ray.

### 4.9. Laser Confocal Light Microscopy

*H. armigera* fat body cells were transfected with the construct of pAcV5His-Red-Nrf2 (0.5 mg/well) for 24 h. The supplemented medium was discarded and the new supplemented medium (which contained 10% of fetal bovine serum) were mixed with 2-tridecanone (final concentration 125 μM) or control, added into the wells, and incubated for 6 h. After incubation, *H. armigera* fat body cells were fixed in 4% neutral buffered paraformaldehyde (NBP) for 15 min and then treated with 0.5% Triton X-100 at room temperature for 10 min. The cells were stain by DAPI for 10 min. After washing 5 times with PBS, visualization and localization of Nrf2 in the fat body cells were analyzed by laser scanning confocal microscopy Fv1000 (Olympus, Japan), and processed with Photoshop CS, version 8 (Adobe Systems, San Jose, CA, USA).

### 4.10. Statistical Analyses

The test data were expressed as “mean ± standard error (SE)”. One-way ANOVA was performed on the test data using GraphPad Prism version 5.0 software (GraphPad Prism, San Diego, USA), and the expression data before and after treatment were analyzed for significance using Student’s t-test.

## 5. Conclusions

Taken together, we found most P450s, GSTs, and UGsT gene promoters have at least one Nrf2 binding site or AhR binding site, and some P450, GST, and UGT genes’ promoters even simultaneously contain a very closely or overlapping Nrf2 binding site and AhR binding site. Using RNAi, RNA sequencing, RT-qPCR analysis, transcription factor binding site prediction, EMSA, and dual luciferase reporter assays, we demonstrated that there is crosstalk between HaNrf2 and HaAhR to regulate the expression of multiple detoxification genes in *H. armigera*. Although much is known about each role of the Nrf2 or AhR signal pathway in the regulation of some insects’ detoxification gene members, the network regarding the detoxification genes which can be regulated by Nrf2 and AhR is still unknown, and there were also no reports about the crosstalk between AhR and Nrf2 that exist in insects and can regulate the detoxification genes’ expression. In this study, we found many P450s, GSTs, and UGTs gene members can down-regulate by *HaNrf2* and *HaAhR* knockdown, which may imply that the effects of *HaNrf2* and *HaAhR* knockdown are not limited in response to one kind of xenobiotic, and may also affect the susceptibility to other plant allelochemicals or insecticides in *H. armigera*. Our results provide a new general signaling pathway for revealing the adaptive mechanism of insects to xenobiotics, and explain why many xenobiotics have the phenotype of toxic action and affect the growth rate in insects. Elucidating the mechanisms underlying detoxification enzyme genes’ regulation mediated by AhR and Nrf2 transcription factors will illuminate how detoxification genes’ expressions are coordinately up-regulated in insects that have acquired the tolerance ability to defend against plant allelochemicals or insecticides. The results of this study improve our understanding of detoxification genes’ regulation in *H. armigera* and provide more insight into designing effective pest-management strategies to reduce the overuse of insecticides. The transcription factors HaNrf2 and HaAhR could serve as potential targets for pest management.

## Data Availability

Not applicable.

## Supplementary Materials

The following supporting information can be downloaded at: https://www.mdpi.com/article/10.3390/ijms232416126/s1.
