# Supplementary material for: A General Signal Pathway to Regulate Multiple Detoxification Genes Drives the Evolution of Helicoverpa armigera Adaptation to Xenobiotics
Source: Int J Mol Sci. 2022 Dec 17;23(24):16126. doi: 10.3390/ijms232416126 (PMC9788003; doi:10.3390/ijms232416126)
Supplement: Supplementary file 1 [file ijms-23-16126-s001.zip › Table S1.pdf]

Table S1. Primers used in this study

Gene	Primer name	Sequence（5’-3’）	Application
AhR	AhR-Y1	5’-GAGATTGTCCATTCAGAAGATCGA-3’	Real-Time PCR
	AhR-Y2	5’-TGTCAAGCCTCAAGAACCCAGA-3’	Real-Time PCR
Nrf2	Nrf2-Y1	5’-CTTCTCTATCTGACGGTGAATGGT-3’	Real-Time PCR
	Nrf2-Y2	5’-CATACTTCACAACGCTCGGTCTAC-3’	Real-Time PCR
EF-α	EF-Y1	5'-AGGAGTTGCGTCGTGGTTA-3'	Real-Time PCR
	EF-Y2	5'-GACTTGATGGACTTAGGGTTGT-3'	Real-Time PCR
Nrf2	AscI-Nrf2	5’-AGGCGCGCCATGCCTGCAGGTCGACGATTTGGA-3’	Full-lenghth *Nrf2*
	XhoI-Nrf2	5’-ACTCGAGCCTGTCCATGTCGTCGTCTGAAGTGC-3’	Full-lenghth *Nrf2*
Red	XhoI-red	5’-ACTCGAGATGGATAGCACTGAGAACGTCATCA-3’	Full-lenghth *Red*
	Pmel-red	5’-AGTTTAAACTTAACAGGTGGTGGCGGGCCT-3’	Full-lenghth *Red*
AhR	KpnI-AhR	5’-CGGGGTACCATGCCTTCGTCTCCTTCTGCA-3’	Full-lenghth AhR
	EcoRV-AhR	5’-AGATATCTTGAGTGGCTTCAGCGGTATGATAA-3’	Full-lenghth AhR
GFP	EcoRV-GFP	5’-AGATATCATGGTGAGCAAGGGCGAGGA-3’	Full-lenghth GFP
	Pmel-GFP	5’-GTTTAAACTTATCTAGATCCGGTGG-3’	Full-lenghth GFP
AhR	dsAhR-F	5’-T7-GAGGAATTGCAGCGTCAGAT-3’	*AhR* dsRNA synthesis
	dsAhR-R	5’-T7-AGCGACGTAAGCCAGATCAT-3’	*AhR* dsRNA synthesis
EGFP	dsGFP-F	5’-T7-CAGTGCTTCAGCCGCTAC-3’	*EGFP* dsRNA synthesis
	dsGFP-R	5’-T7-GTTCACCTTGATGCCGTTC-3’	*EGFP* dsRNA synthesis
Nrf2	dsNrf2-F	5’-T7-CAACAGCACGATCCACTCTACT-3’	*Nrf2* dsRNA synthesis
	dsNrf2-R	5’-T7-GTTTCCCGAACATATGGTGTTT-3’	*Nrf2* dsRNA synthesis
CYP4L11	CYP4L11-Y1	5’-CAAGACGAGCGGAACTAAGACGT-3’	Real-Time PCR
	CYP4L11-Y2	5’-TGTTCCCTCACACCTTCATCG-3’	Real-Time PCR
CYP4L5	CYP4L5-Y1	5’-ACAAGGCGGGAAGAATTGAGAAA-3’	Real-Time PCR
	CYP4L5-Y2	5’-TCTTCCCTCACACTCTCGTCA-3’	Real-Time PCR
CYP4S2	CYP4S2-Y1	5’-TACAAGAATCTGGCTCCACAA-3’	Real-Time PCR
	CYP4S2-Y2	5’-CGACGCTAACCTTATTCTGTTCTC-3’	Real-Time PCR

CYP6AN1	CYP6AN1	5’-CAACAGATCGACGAAAATTCATT-3’	Real-Time PCR
	CYP6AN1	5’-CGACTCCCCTGTGATTTTACCTTTTT-3’	Real-Time PCR
CYP6AE20	CYP6AE20-Y1	5’-GAATTATTATTGGCTCAGTGTATAGTA-3’	Real-Time PCR
	CYP6AE20-Y2	5’-TTTGTCGAGCTGTAACCCTGTG-3’	Real-Time PCR
CYP4M7	CYP4M7-Y1	5’-GAAGGACTGATTGACAAGACTGG-3’	Real-Time PCR
	CYP4M7-Y2	5’-TGATGAGCGAGCAACATGAAC-3’	Real-Time PCR
CYP18B1	CYP18B1-Y1	5’-CTGATGGCTGAATTAGATGGATC-3’	Real-Time PCR
	CYP18B1-Y2	5’-GTTCTCCAATAAGTACCAGGCCAA-3’	Real-Time PCR
CYP6B2	CYP6B2-YI	5'GGGCGTAGTGATGGAAGAATTG3'	Real-Time PCR
	CYP6B2-Y2	5'CCAGATGTGAAAATTGGTGTAAATCG3'	Real-Time PCR
CYP6B6	CYP6B6-Y1	5'TTCAAACTTATACCATGTCCACAATT3'	Real-Time PCR
	CYP6B6-Y2	5'CCAATTGACGGAGCTCTAGAATCA3'	Real-Time PCR
CYP6B7	CYP6B7-Y1	5'GAATGACCACACCCTGCCTACTG3'	Real-Time PCR
	CYP6B7-Y2	5'TTAATGCTCT CCACGTTTCTCCG3'	Real-Time PCR
CYP321B1	CYP321B1-Y1	5’-CCTTCGAGACTATGAGCCACTT-3’	Real-Time PCR
	CYP321B1-Y2	5’-GATCGCAAACCTCAAATTCG-3’	Real-Time PCR
GSTD1m	GSTD1m-Y1	5’-GCATATGACGCCTGATTTCTTAAAG-3’	Real-Time PCR
	GSTD1m-Y2	5’-CGTGTATAAAGAGTGCCAAGATCAAA-3’	Real-Time PCR
GSTD1d	GSTD1d-Y1	5’-AAACTTTACCATTTTCCTATCAGCGG-3’	Real-Time PCR
	GSTD1d-Y2	5’-TGATCATCCTTCGCATATTTATCAGC-3’	Real-Time PCR
GSTD1s	GSTD1s-Y1	5’-GATTCTGCAATTCTATACGCTAG-3’	Real-Time PCR
	GSTD1s-Y2	5’-GGGTATTTATTCTCGTCAAACTTGT-3’	Real-Time PCR
GSTD1p	GSTD1p-Y1	5’-CAGCCCTCGACGTTCATTTTAATTTA-3’	Real-Time PCR
	GSTD1p-Y2	5’-TTGTTCTCTCCACCATATTTGTTCAC-3’	Real-Time PCR
GSTD1c	GSTD1c-Y1	5’-CCTCGACGTACAATTAAATCCGCAT-3’	Real-Time PCR
	GSTD1c-Y2	5’-ATTGTTCTCTCCACCATATTTGTAT-3’	Real-Time PCR
GSTD1k	GSTD1k-Y1	5’-TATGACTGTTGAGGCCTTAAATATTCCT-3’	Real-Time PCR
	GSTD1k-Y2	5’-GGTCACCAAATATACAGCAATT-3’	Real-Time PCR
GSTD1L	GSTD1L-Y1	5’-ATTAAGTCAGGGTACGAGTTCATTGA-3’	Real-Time PCR

	GSTD1L-Y2	5’-ACGTAGAAATCTTGTTTTGAGCACTG-3’	Real-Time PCR
GSTO1	GSTO1-Y1	5’-TATGCTGAGAGAACTGTCCTTGTTAT-3’	Real-Time PCR
	GSTO1-Y2	5’-TACTTCTCATCCAGGTAAACGTTGAT-3’	Real-Time PCR
GSTS1f	GSTS1f-Y1	5’-CAACTCTATCATCACCAAGAACAACG-3’	Real-Time PCR
	GSTS1f-Y2	5’-TAGAATCAGTAAACGCCTTGACTTTG-3’	Real-Time PCR
UGT40L1	UGT40L1-Y1	5’-CCGAAGATATGGTTCCTGAGTTAGAT-3’	Real-Time PCR
	UGT40L1-Y2	5’-TACTCCCAGATCTAGATACAGCTTCT-3’	Real-Time PCR
UGT40M1	UGT40M1-Y1	5’-GAGTACCTGTGTTCTTTGATCAGTTC-3’	Real-Time PCR
	UGT40M1-Y2	5’-CGTGGTAAATTGCAGACATTTCTTTC-3’	Real-Time PCR
UGT41B1	UGT41B1-Y1	5’-TCATCACTACTTGGACTGTCAATGAC-3’	Real-Time PCR
	UGT41B1-Y2	5’-GGAATATCGAACCAACTATCAACAG-3’	Real-Time PCR
UGT39B2	UGT39B2-Y1	5’-CCTACAAATACAACGCTCCTTTAGTC-3’	Real-Time PCR
	UGT39B2-Y2	5’-ACAAATTCCTCATTCTACCCCAAAAC-3’	Real-Time PCR
UGT44A2	UGT44A2-Y1	5’-GACAGTTACAAAATACTCGGCATCT-3’	Real-Time PCR
	UGT44A2-Y2	5’-TTTATACACTTGATGTAAGCCGTCA-3’	Real-Time PCR
UGT43A1	UGT43A1-Y1	5’- ATTTTTCCCTATAATGGTCGAAGTC-3’	Real-Time PCR
	UGT43A1-Y2	5’-GTTTGAAGTAGAGAGCACTTTCAGG-3’	Real-Time PCR
CYP6AE14	proCYP6AE14-F49	5’-GTAGGTACTTTAATTTAGAATTTTA-3’	Promoter amplification
	proCYP6AE14-R51	5’-AAGCTTCGTTGTAACTGTAT-3’	Promoter amplification
GSTD1s	proGSTD1s-F50	5’-TATTTATTCAACAAATGATTTAAGTA-3’	Promoter amplification
	proGSTD1s-R51	5’-TGAAAAAAACATATGAAAATATACTG-3’	Promoter amplification
UGT40M1	proUGT40M1-F50	5’-TAAATTGTGAATGCTAAATAAATG-3’	Promoter amplification
	proUGT40M1-R51	5’-TTAAAACACGTCTTTATAAAACAA-3’	Promoter amplification
CYP6B6	942-KpnI	5’-GGTACCAATATACAAAGTATATCGTACCTAAC-3’	Promoter sequence
promoter	679-Kpn1	5’-GGTACCCGAAAAATCACTCCTGT-3'	Promoter sequence
	252-Xhol1	5'-CTCGAGTTTTGAGGAGCTGTTTACAC-3'	Promoter sequence
	151-KpnI	5'-GGTACCGCTTTTCTAA ATTTTAAAAG AAA-3’	Promoter sequence
	pro6b6-kpnI	5'-GGTACCTACTTGTAAATTACCTGCCA-3'	Promoter sequence
	pro6b6-BglII	5’-AGATCTCCTCGCTGTTCACAATG-3'	Promoter sequence

CYP6B2 promoter	129-KpnI	5’ACAAACCAATATTTTTCTGCACACCA-3’	Promoter sequence
	579-KpnI	5’-CATTCAAACACCTTCCACGATACAT-3’	Promoter sequence
	897-KpnI	5’-TATTTGGGTGTTATGGTGTAAAGTTCAA-3’	Promoter sequence
	1355-KpnI	5’-CTCATATACCATCGTTCATAGAAATT-3’	Promoter sequence
	1959-KpnI	5’-GTGTCAACGTTGTCCCTAGG-3’	Promoter sequence
	Pro6b2-BglII	5’-TTTGGAGGAACTGTGATCTTAGTAA-3’	Promoter sequence
CYP6B7 promoter	250-KpnI	5’-TAAGTCATGACAATACTATTTCGTAAAT-3’	Promoter sequence
	350-KpnI	5’-AAGAATTAATATGAATTGACATTTGAAAT-3’	Promoter sequence
	730-KpnI	5’-TTAAACGATCCAAGAACACATTGCAA-3’	Promoter sequence
	1932-KpnI	5’-TATGTAAAATCTAGACCTGCAAGCTACA-3’	Promoter sequence
	Pro6b2-BglII	5’-TTTGAGGAGCTGTTTACACAACC-3’	Promoter sequence
