# Supplementary material for: A General Signal Pathway to Regulate Multiple Detoxification Genes Drives the Evolution of Helicoverpa armigera Adaptation to Xenobiotics
Source: Int J Mol Sci. 2022 Dec 17;23(24):16126. doi: 10.3390/ijms232416126 (PMC9788003; doi:10.3390/ijms232416126)
Supplement: Supplementary file 1 [file ijms-23-16126-s001.zip › Table S6.pdf]

>HaOG200003 CYP18B1
AAAACTGCGGAAACGTAGATTTGAAGGCAATGTGTCAAATGACAAGGGCAAAATACGTAAATATGTTATGTA
AAGCGCTCAATTATTATCACGAAATGGACTTGAGAGAGATCAATGGACTTATGTACTTACTTATCAAATGCGT
GATCATTCGCAGCTGCATGTTTCGTATGTAAATGCAGTGATAACGTATGTACATTTTCCCAATTCTACAGATTCT
TGAGAAAGGGCAATGACCCTTGATTTAAATGTAAAAGCAAAGAAACTCTATGGGGAAGATGAGTTTATTTTT
CTTCAGGAATTCCCTGCTGGACGGATTAAGATTTAACCGGGAGAAGGACTAATAGTCCGTAGCAATCGCGCC
ATAACGTCAATTTTACCGGGGCTGCGGGATTGTTCGAAAGTTACCGCGGCCCTGGTACATAAAAGGCCTAAG
ACGGAACACGGTCTTTTTAGTCAGTAAGAGTCTGACACTCTCACCGCTGCTAACCCACAGTGGGAGGGGTC
ATTTGTCGATTTTGATCGTCGTTAAAAAAAAAGGCATTCGTGGTGAAAGTGCTCACGTGTGTCCTACACCTTGT
GTTGAACGAAAACTCCTGGCCACAAGACGTGGTGCTCCGTCGCTTCAACGGACAAGTGCCACAGGACCAC
ACAGTGACATAGTGCATAGTGAAGTGTCACTAACATAGATTTACGTGTTTATTTACTTACATATTTTGTATATAA
CATGTTGTATGTGTCTGTTTTTAAGGTATTTATCCATGGGCCATTGAACAATCTTAGTAATGAATATTCTTAGTG
TGTGAGTTAAATTATAGGAACAAGAAGAATATTTTAAATCGTTAAAAATGAATAAAGAAGGTCATACATGACC
GGGAAATCCCCAACGTCCAAATGATAATTTTCGGACATGATACCAGCTAAACACTACCTCAATAAGGTCTAGC
ATTGCCCAGTATGTGGCGGTTTCCCAGCACCTCAACTAGGTCATCTGCCTTATCTAGTTTTTAATGATATCTAAA
TAAAATTCTCAAACACCGGCTTGACCTACATTTATATAAAACCCAGTTTTATACATAAGGTGTAGTCTTTTGTGT
TGGGAAGTATGAACATGTTTAAAATTATTTATTTTATTTAACACTGTCCTACCTGATTGTCAATAAGCTGTAAGT
AATCCTAGGTAAATAATCCATGTATGGAATAATTAAAAAACCTCTTACTAGGTATAAAAAATATGAAAACTGTT
TTATTTCTAATTTACTGAACAATAAAAACATACTTTCAATTTTTTAAACGTCTTTGAATTTACTACACTAAATTTA
ACTCTATAAAAACATTTAAGAAAGTTAAATGCATTTTCTAAGAGCATTCACGTCCAAAAGTCGTTATTAATGAT
TCCCGCTACCTAAGTAGGCCACTTTAGCACAAAAGCCCTTTTTAGGTCACTAGGTCAACAGTAGAAAATGCT
TCACTAAACGCTTTAACAGAAAGAAAAATAATTTTCACAGTTAAACATTACATAAATAAAGTTTCAGTTTTGTT
ACAGATCAAAAAAGGTGAAGGATTAGGTCAACGAACTCATATTTGACAATCATAACAACGTATGATAATACGA
TATTTCCAGTATTCATTGATGTTCAGTTGAATTATTTATCAGCAAATCAATTCGTACACGTAATAGATAAAAATG
GCAGCGGCCGCTTAGTACTATATATAAAACGACTCAAAGCACCTTGCTTCATTATTCAAAACCAGAGAGTATT
CTGCACACTCGGTCAAGTTTTCGCACCGCTCTTGCATAACAGCCAAAATTTGTCTATTAAAAATTGTATTTTAA
AATCTACTGGCATTATTGCTTCATATTTAAACTTAAAAAATATTGTGGCAATTTTGCTCTTTTTATTTTAACTTAT
TTTTCGTTGAAACGTATTTTATTTTACGTTTCTTTAAAAAAACAACGGAACGGCATATTTTAAAAAGCTTTTTT
AAAATCATCCAAG
>HaOG200077 CYP4L5
GGTAGGTAAGATACTCTAAGGAATATACACACAATTTTACGACTAAGTAATATTTTTACTCCTACACCGCAGCT
ACCAGCAGCTACTTTATTTATTTAAGTCACAAAGTAACTGAACGTTTTATCTGCTCCAAAAATACGAAGTTTTA
CAAACCGCCAGTTTCCAAACAATTCACGTTCAAAATCCAGAACAAAAGCGTCAAAAATATCAGTTTAAAAAC
TGTGATATTGCAACATTCGGTCTAGTGTCCTCACGTCGCCGTCGTCTTCTCGTCTCGTGGGGCATACAAATCTC
TATGTTAGGATGTTGTTAGTGAATTTATCAATGTGTATGCAGCAAACTAGACGTTTGTACAAGAGGATTTTGG
AATCAGTTTAAATTAGATTAAACGGTAATGGTTACATTCCCAGTTTGAATAATAAAGGGATGAAAGCAGAGTT
CTAGAAAAAAAAATTGTTGCAATTTCTAATAAGATTAAAAATATGTGTCAGTTTCCACTGGCTGACCTGAAGTA
AGTTCAGACTTATTGCCGGCTTATAGTCTATGCAACATTTGAAACACTTAAAAGATGAATCTAATACCCGAAA
GAAATACATGACATCCGATAACAAAAAGATATTAATAAGTGGTGGTGGTACAGAAGAGCTTAAAAATGCCAA
TTGCATTTATAAATTTTGATACATACTCCATATGACTTAGATAATACTGCAAAGTACAGTAACAAAACTAGAATT
CACATCATGTAGTGCGCTCGCACATTGTATACTAAGCGCCTCATTGCACTTTGTTACGACGAATTTCTTGGCAC
ACGCCCACCTGGGCCTCTGTAATTAGGCTGTATGACACTATAATACCTTTGCCTGTTATTTATTAGGGGTTTTCT
AGAATAGGACGCGGGATTTGAAATTAGATTTCGATTACTTTTTTTTTATTTCATTAGTTTTCTAATGTCGTTCA
AAAAGAGCACTGTTTTTATATTTATGTTTAGAATGATTTGATTTGATAGTATGTTCCTTTTAATAATGACCCTTTC

TATTTAATTAAGTATATGCAGATTTTTCCATTACTTAACTAATTAGCAGTTTACAATGGGTACTTAGTTGATAATC
TATTTTCATGAGGTATTATTGTAATTTGTATAGTGCAGTATTCTAGACTTTCCGTCCTTTTAAATAATAGTTGGAT
GACCGCACTGGGTTAGGTCTTATGTAATCGGCTTATAGCAGTTGCTACTAAGGTAAATCGTGTATTAATTAGAT
ATTTTTTGTTGAAGGTTAATCAATCGTAGAGAAACGAGTTTGGTGTATGTACTAAAAATTCAGTTTTTAAATG
GTTAAAAAACTTGTTTGTGGTATAGAGATAAACAAGTTGGGAATCAACGTATATCTTTTAAAAATATCACCTG
GCAACAAACCTTTCTTATCTGTTAACGAATATAAAGGTTACCTAGAATATACGGAATAAAGATGGGTCATTGTC
ATCGGAAAAATAATTGAAATTGAATTATTTATACGAGTGTTTACAATAAGTTGACGCTATACCTATACACATTGT
AAACGTTAGTTATTCTAAAGGTTAATTAATTAACTATTACATATATAAAATATACTCATTTGCAGACATAACTGAT
AAGATAGGAGAGCATTTACGGCAGTAATGCAATTAGATCATTTTCTTACACAATAAAGAAACAATTCATTACT
TGTCTGTCATTACAATTTATTTCGAATTATTATATTATCATTAGGTACCTAATGATTGTAACTATTAACGTTAATAG
TAATAAAGTTAATTACCTATGTCTGAATAGTTGTGTGTCCATATTGTTAAACAGTAAACGTTCGATATTTGAATT
CATTTCGCACACTGACATTGACCGCTACAGTTTTTTTTTAATTCCTCACAGATAATCGTGCTAACCGGCTTGTT
GACTCGCATTTTCATTACAAACCTCATTGGATGTGGCTGAGTAGCACGTGTCAGTGTTGTGATTAAAAAAA
>HaOG200076 CYP4L11
AAAAATATTACTGTAAAGGTTAAGTAAATAGAACAGTGAAAAAAAATATGTTTATTCATTTTTATACATATAAAT
TACATTTTTAAGTATTAAAATTAAAAACGTTTAAAAATAAAAAATAGTGAATATTTTTATATGAATGTGGGGCC
CTGACTTTTTAATTAATATTTTATTATTTTATAGAAGGGCCCAAATCGGCCCAATATACAGGGTTATTGGTAAAT
CACTAACAACCTTGCAGGACTGTATTACTAACGTCATAAACTACAACTTTTGTTCTATGACTTTTTATAAAACAT
AATACATTTGCCAAAAACAAACCTACAAGATTTCATTGGTCTATCTAACACATTTCAACTCTATTTTGCTATAGT
GATAAGCACGAGCGATTAGTCGACCTTACGATCACTATCCCTGATGGCATGCCGGAATTGCCCGTAATCTCGT
AACCGCTGTGTGGCGGCTAGCATTGTTACTCAGCTAGGTGTAGGGATGTTTGGGAATCGTATGGCGTATTGG
CGACAGGCTTCCGAAACATTGTCATCACACCTAGCCAGCGTTCGCACCATTTCCGTGCACTCGACATTTAAAT
ATCGATACGCCATACTGTAAAGATAATTACGTTCGCACCACCGAATTTCCCCACAAGGACACGTATGATGCGA
CGCGGGCGCATAAAAACGACTGACGGCCGCTCGTAAATAGGTCAAACGCTCCCCCCCCCCCCGCCCGAGCC
CCGCTCCCTGCTCCCCTCCGCTGTCTTTTGTACTACTGTTTACTGTGCTTCGCATAGACTTCGTAACTTTTAGA
GCGTACACTTGAAATATTTTAATTTTTGTGTGAAAAATTTGATGAACTAAAATCTGAGAATTATTAAAAGTCAT
AGAACAAAAGTTTTAGTTTATAATATTAGTAATATGGTCCTGAGAGGTTGTTAGTGTTTTACCTATAACCCTGTA
TAGGGGGCTACCTTGAAAACGGACATAGACTATTGTTTATCATGATGCCCCGCGCCATCAAAGATAACAGTCT
AAATTTAATACTCTGGTCACAGTGAATCAGCATTCCGTAGGAATCCGTAGGTATGTTCATAGTCCAAGGTTAA
AAACCGCGTTCGGCTGAGTAAAGAGCAAAGTTCGTTTTTACAGCGGTCTGTGAAATAAGGCGACTTACACA
CGGTACGAACGCTGAAGCAGATGTCACGTTAATCCTCTATGAAATCCACTTAAATATTTTCCACGAAGACTAT
GCAAGCGGGTTCTTGATGTTTTTTTTGTACCACATATTGCTAAACATATAAACTTACCTATATAATTATCCATTTA
TTGCGCTATACTGACTTTCCTGTTACTTCCAAGACGTTACAGCAATAGATAAGATAACATTATGGACAGTAACG
TTGCATCAAAACAATCCGATAAGTTAGATAGCAAAAAGAGTAGTCAAGTGTGAGTCGAATTAGATCGGTGAA
GGTTCCAATGTATATAGGAATTTGCTCCAGCGAATTGCTTTTCATAGTTTCGATTACGTAAATTATTTTTTTGGC
AGCGTCTGTTTACACAAATAGAAATGCTCGACTGAGCAGAGCGGCGGAGACTTTATGGCTACTGTGAATGC
GCAGTATTTTAGTAAACAAACTGCAAAAAAATAGTTCATAATCGCGTTTTCTTAAAATCTACCAGACTACTACT
TTACTAAATACATGATTTTTCTACGATAGGGAAACGAAGTATTCCCGTAAGAGTATTTTTTTTCTTTTTTTCAG
AAGTTCAGAACCCTAAAAACATTATCAAAATATTGTCTGTCTCTATCGAACCGATTTATATTAATCTGCAATGAT
TGTGACAATTTCACGATTAAGTTGTAGGTATAGCAGTATCTTCGTTTGTGTATAAATAGCCTACGTTCGAATTTT
GGCGAATTCATCTCGAACACTCATTGTCAGTCGGATAACCGTGCGAACCGGCTCGTTCACTAGAATTTTTATT
TTTTCGAAAAA
>HaOG200082 CYP4S2
AGTAATTTGAAGTGGTTTATATAAATATATCTAGTTATTTATACGTTGATGCTATTATGATATAAATGGATAAATAC

AATTATGTTTAGACACTGATAAATAAGCCAACAACATAGGTAGTAAAGAACAATGTATTTTCATTGATGATTCC
ATTGCAAGGTCAAGCTTAATAAAAACTTAGTTCAGGTAAAATATGGACGGTTTTTTTGGATTGGTTGTTGTGT
AACTTGTTTAATGTTTAAATTATAAGCTATTTATGATTTATTGGTTTTACGAAATTATTATTCAAGTGTTATGTAA
TTATGTTAATAAGATCTGAAGAGGTTGTTAAACTCTATCTACTGATCTTTATACCAAACTTCTAATAACATCCAG
ACAGTATCCCTCTCTCATTTAGATTATTAATTACATTTTAATAGTCAGGTCAATTATTATTAAATCGAACATTTCAT
AAATTTCGAGATTTTCTACGCCCAAACAGTTCAACTAAATCTGAGTGCGATGTAACGTAATAATTATCTAATTT
TAGTCCATTTGCTTAATAGCCGTTAAATCACATACTGAACACAGTCTAAAATAGCTCATCACTCTAACCAATTAA
GGCTCATTATCGCTTAAACCTGGATTAAATACGGCACACATTAGTTCCACATTAGCCAATTAGCTTTGACCACA
GTGTAAACGGCTTATAGACAGGAACAAGTTTACCATTTCGCCAACAGTTGGTTACGTAATGGGTTCGTTAAAT
AAAAACTTTTTAACTATGAACTAGAAGCTTTTGCGTTAACTTCTTCTAATTCGATTTGGAGTGCTTTAAGATTA
AACAATCTAATAATATGGTCAGTATCAAAAGTAGCTGAATCAATCAATATTTTCAAAAGAGTCTAACAACGTAA
AGGGTCATAATTTCTAAACAACACAAAAGCCAAAATTTACTTGACATTCTAAACTTCAATAAAAGTAGATAAA
TGCTCTGGTGAGTTATCAACTTCACGATGTTTAGCAGATTTGAAAATTTAAATCCATCCAGGTACTTTTTGAA
GCTGACTGTACCTGTACATTCGAATGTTATTGAACGAAAAACAAAACATCGGATCGAATTTATGTAAAAGCTT
CTTCTCGTATAACATTAAACTTTTGATTAATTTATTGTTTAAAATGTTTAAATTGTCGTATCGCTAAAATATAACG
CTGCGATGGTAAAACTCTTGAGAAAAATCATCGTGTCTTTGGGGAATTAGAGTAGATTAAGATATAATCGGA
GTTTTATTTTGTTGGTGAACTCGGGCGTAACATTGCTTTCGCCAGAAAGGTAGGCCCGCGCTATAAATTCTAA
ATTAGTTTTACTTAGAAGTCGAACCGGGAATTAGTTATAAGTTACAAAGGGATTTTAAACCCAAAAAATGGAT
TTAAACTACATGAATGTATCGTTAAATGAATGGAAAATAACCATCCTATATTTAAATATTAATTGTTTGCTCTGAT
TACATTAATTTACTCAGAATTTTGACCCGTTAATATAAAAATACAAAAGATATGCTACTTCAATCTGAAAATTGC
TTGTTGCTACAAAATATTGTTCTTGGAATTAAATTAATTCCCTGATGCTCAGAGCATATTTTTGGTTCGTTATTG
TTTTAAAATATGGATTCCGACAAATGATTAAATGTGTCGATTCATGGATTAAATTCGATACAGTTTTTGAGAAA
ATTGGCTAATTGTTTATGAGTCGGATTATTTTTATTTATACAATACTTGGTAATTTTTGTTTGACAGATATTACAA
AAGAAAAATTCTGTCAAATCGTATTTATTAGACAGATATAAAATAACTTCCAAATTAGGTACATAATTAGTGGG
TAATATTTTTTGTATGTTCAGAAGATGAAAAACATGCACATTATGACTGTTTTTGTGTTGGAAACTATGTACGG
TGAAAAGGTTTAGTTTTTAGTGTAACATTTTATCATATTTGTTCCCAGGTCTAATCCCACAGAAGG
>HaOG200098 CYP6AN1
TCTGAGAAAGGTACCTATACAATGCAAGACGAACTAGGAAGTTATATGAGAAAATTATTCAATTCAATTTTCA
TTGATGTTCTTAAAAAAACACTTCGGCATCAACACATTAATTTAAATTAGGTAATTATTAATTCTAACCAAGGTT
ACTCATTCAACTTAATTACCTACCCACTTATTTAGTTACAATGTTCTATTATCTACCAGGAAAAATATACATACCT
TCATAAGTACTCCAATCTTTAAAATCTAACCAATCAATTAAAAACATAAATACACATTCATCAATAGTCTGACTT
TTGTCATAATACTCTAAAATTTTTGTCTACGAGCGACCGAACAAAGTCCAAAGTCTATATTGTACCGAGATATAT
AATACTGAGTTATGAGGCTCTCAACTCAAACTGGTTTGAACCATCCAGCTAGAACGACCTATGCTAGAATTTC
GCGAGTTTTTGCCAAATTGTGATCAAAATAATTATTTTTTTAACACGGAAGAACTGAGGTATGTATTGTTAGAT
AAAGTGTTTTCTTAACCTTTAATAGTGTAAATAAGTTATTATCAAGTGTAAGGAGATTGTGTTAAAAATAGACA
ACTTGAGTCCAAAGGTCGATTTTTAGCTTTATTTGGTGTCTGAAGAAAGTAACTTTCTTTAGTGAACGGGTA
AAGTGACATTTATGATCAAAGTCTAGATCAATCATGCATGATAGTATCATGATTTATTTGTGTTTATTAACTTATC
GGTGTTTTATTGGGTGTTAAGCCTTTGTTTATCGTATCAAAACATGTTTAAAATCTAGTCTATTTTTGAAGTAG
GTAATTTAATTGCTACATCATTTGCATTTAATGTTTTTTTTTGCAAATTAAGCTAAACATTGCCGCATTGAGCAA
GCGTGGTGATTAATGCTCAATCCTTCTCCGGGTAAGAAGAGGCCTGTGCCCAGCAGTGGGACGATAAAAAA
AGGCTGTAACAACAACAAGCTAAACATTATTTTTATTGTATCGTGGGTACCTACATTGTAATATCTTATTCTTGT
CTCACCTTTGATAACTATTTACTATTTTATTACTAAGGAAGGTGATAAGAACATGTCCTTATCACCTTGTCTAGG
CATACAGCTGGTAGAATATTGAGATAATAGTTGAAAGATAACATCGTATAAAAATATATTCCAGTATCAAGCAG
TTACACAATTGTAAGTAAACAGCACTGTTGAAATTCAACATAATTATCGTATCTTTTTGTAGTTTATCGATAGTT

TCTTCAATGCCCTAAAATAATTTTCGACAAACAAAAAATCCAATTCAGTTTTTCGAAAAGATTATTGTTGCTCA
ATAGAAGTAAGTCCTTTTGTAGAGGTAGTAATACCTTTTTTCCTTTTGATGTTTGAGTAGCAAAATTGATAAAA
AAAATCACATCTTATCACTGATATTATTTATTTATAGAGTACCTAATTATTTGTAGTTAGTTAAATACAAAGATAG
ATACTTCACCACATGCCACAGACTTAATTGGTGATGCACGTAGCAGGATATGTCTATAATATAAAATGTTGGTG
CCAATTATGTCAAAGACTCGTATGTCATTACATCCATTTGAAACATACAATTTAGCACAGTATGTATGGGACAG
ACAATGTGCATATGATGTAATTATGATGCAATCTATAAACTTAAAACATTATTTTGTTTCATTACAGGTCGAACA
AACTACCAGCTATTTAGTAAAAATCTGAAATAGAGGAAAGGTACCAACAGAAAAGTGTTGATAAACCGCAAA
AATAAGCTAAACAGAGTCGTTTGTTTCCACCAACTATCGCGCCGCATTATAATCATCTAATATAGAACAATAAA
ACGTGTGTCCGTAATCGCTCACTATACGGCTGTGGCCCGACGGAACCCCAAGTAGGTACCGTTCACACAAAC
AACACAAGATAACGGGTCCCACCGCACACTCCGCGGTTAACTCAGTCGCACGTCTTTAACCACGAACACAAC
A
>HaOG200095 CYP6AE20
GGACCATTGTAGGTAAATAACCTCCGATGTTTTATAATCCAAAAAAATAATTAAGATGAGTTAAGTCGATAAA
AAAATAATTGTTTTAAACTCTTTGTAATAAGGTTTTTACGCTTACGTGCCTAGTTTTTCTTCGGGATAGTTTGA
CAGATCTATTACAAAAAACTTCATAAAAAATTGAGGTAAATCGTATTTTTTTCCGACCTACTTTTTACGTTTAC
GTTTTGATTCACAACTTCACGAGTGAGAAATAAAAAAAATGTTTGAACCGGAGTTTAACGCCGACACTAAAC
AAACTATTGATTGATTACATAATTTGTTAAGCTATTAACATAAGTTAATAGTCAAGTGTTAATAGCGAACGAAAT
ATTTGATGTCAAAGGACTATGACAACTAAGCCTATCTTAGCTGAAATGGCACGGGATTGTTCGAAAGAGTTA
CCGCGGCCCTGGTAAATAAAAGGCCTACAACGGAGCACGACGGCGCTGCGGTTGCCCGAGTCCTGTGGGA
AGAACGCATCCGGATAAACAATAAGCTTTTAAGGGGGTTGCGGACTTGGTCAAAGTTAACCAGTCTAACCA
AGGGGTATCGGGTTGCCCGGGTAACTGGGCTGAGGAGGTCAGATAGGCAGTCCCTTCTTGTAAAGCACTG
GTACTCAGCTGAATCCGGTTAGACTGGAAGCCGACCCCAACATGATGGAGGATGGATGGACTTGGATCAAA
GTTAGATTAGTTTAGCTTAGCTTACGCAGTTTTTCATACTTATGTACCTAAGACCGCAAATTATGCAGTCTATGC
AAAATTAAGTACAATTATATTAAAAAACGTTTATGCTATGAACAAAGCAAAACGACGACCAACCTGTATTTTTT
TTCAAGTAAACAAATGAGAACGAACTATGGGAGTGTCACTAAGGTATTGCCAATTTTAATCTATACTTACGCG
AATATTAGACATTGAAATAAAACTACTTTTACGGATTTTCTCGAAAGTAAGTAAAGGATCCCGAACGTAGGGA
TTAAATAATATTAACATAACCGCAATAAAATCCGTAAAAGTAGTTTTATTTAAATGTTTAATTTACACTTTTAACC
TAGTTCTGCAGTTTCCATGGTAAATAATCGCTATTTCTCTAATTTGTGAAGAAAATAAGCTCATACACGCGGCT
TGCGATATCCTACCAAATAAGTATGATTACAAACGTTCATAGGCATTAATACCAACATCTATTAGACTTATTTTAT
AAACGTGAAAGGTTGGATGCAAAATACAGTACTGATTTTGATAACTGGCTAATTTTAATTTACCTAGGTTCGG
GAAGTAGCTCAATAGCTTGTCTATACTAATATTGTATTTTTTTTTTATCCTAAAGATTCCGAAATTACGGAATTG
ATATAAAAATCATTGACTTTTCAGCTTAGCCGGGACCGGAAAGAAATTCTCCCGAAACGCGGGTGAAACCG
CGTGAAACAACTACTATCTAATTTGTTTACCCAAATGTAAAAAATATTTTCGAACTATTTTCGTAGTTTTATTTG
ACGGTGAGATGAAACGGAATGGGGTGGGTATGTTGGAAATAAAATAGGTATACAATTAAAAGTTCTTAGTTA
TCTTTTCTCCACATCAGGATAAAAGGATCAGGATTTTTCTAGAATTGGAAATACTTAATCGTATTTAATTATCCA
AAATGACCGTCAAAAAAAACTTATTTTATATAATGATGACTTGTGTTAACAAAGTACGCTTCCAAACTAGACTC
ATGTCTGTAACACATAAGAACCATATTTCCGTCTATCAGCCGCGCAGATAAAGGGGGTAGACTTTCCGCACAT
CTAAACCAAAAGAATTATCTACTATTGATAATAACACCTTCTTAAGTGATCAGTGTCTATGTGTGCGTGTGTGCT
ACAATGTTGCAGCTACGTGTGCGTGGTACCAGTGATCTACAAAAGACACTGCTTCGGCCCACAAACGCCAGT
TGTATCCAACGCGGTTACCAA
>HaOG200080 CYP4M7
AGTACCTATGTAATGAAGGGTGAATCTACACGGTGCAAGTAGCTGGAGTAAGTTAACATGAGCAAGTGACA
AGAGCACACGGGACACTTGGTATATGCGCGAGTCTCTGGACCCGCACGTGTTTAAAACGCATGTAACCCGC
ACATTTGCCTCAACATACGCAAGCTACTTGCACCGTGTATAACGTAAGAATAATAACGTAAGAACGTAAAGTA

TAATTTTCCTTGTCATTTATATCGTCACCTGCCAAAACTTACTTTATATAAGAAACCTTTTTTATGAACACTGTAT
TAATTTACATTGTAAGTATGCATAGTCAAGCATAATCAAGTTATTCAATAAATATTATTTTTTAATTCGTTTTTTAT
TAATCATTGTTACACTGCAAATATTGTTGATTGATATGCAATGATTCAATACGCAAGTGTGCAACCATATCAATC
ACAACCCTTGGCGATAAATGGACGACAAAAGTTCACACTGGCTGGAATTTAACTACTTATGTATATTTCATAG
GAGAAGCTTTGAGAATTTTATATTTACCATAGACATAACTTCAAACAGATGAAACTCATTGGTAATTTGCACCT
CCATCAAACGCATTAAGCCGGGGATGGCGGCTTATAAATATAAAAATTCGAATAAAAAAAAAAATGCATACC
AAATGTGTCTTCAAGTTTGTTGTAAGAAAAATCGTAAGTAAAGGAAGAGAATGAGTAGGTAGATAATTTAAT
TGGCGAGACCTAAGACGTATGTGCCTAAATACATTAAAAAAAATAAGATATGAAATTGTCTCGGTTACACAAT
CGTCACAGGTCAATAAGTCAAGTTAGGAGTACCTAAGAACAAAAAAACAGATATTTGATAAAACGTCTAACA
TGAAGTTATGTACAATACTTTTTTTACTAGTCCGTACAGACACTAAGTTTGATAATGCAATTAAACATGATTTAG
TCTTGTGAAGTATTTATTTATTAAGTAGATTATGGCCATTTTCGAGAACTATGAATCTCTAAATAGGAATTAGTG
ATCATAAAAAAATATCTGAAATACCTTGTAGATAGAAAAAATACACAAAAACGATCAATGCGCTGGACACATTG
TGACATAACTTTTTTCTTTTCTTTAATGTTAATCTTAATAATTACCAAGTATTGCTAAATCTCAGGCTTTACCGGA
AAATCGCTCACAATAGCACATACACTTGTAGTAGCTACATTATCCACAAGGATGCATTACATCTTGGCCACTTA
GTTATGTGTCTGTGCAAGTAGGTACTCGCCCTTAACCGTAAAAGGACCAGGAAATAATATGACACTTTTCATG
TCTAATAGTGGTATGGTGGACCATGATGTGCATCGTCCCGTCTTTCATAATTCATAACATACCTATTTACGGTAA
GTAAAACTCATATAAAAACTATTTGGAATAATTTCCACAAGATAACTTTATTGTGACTAAGTTTCGTATTTTTTT
CATTATGTTGATATGATTAATATTATACACGTGCCTTTATTATGCATAAGTATCTTCGAAAGTTATCGTGTGTTTT
GCCAGTTTGTTGATATTCACTGGTTCGCTAGGTCGCTATCAGAT
>HaOG200015 CYP321B1
GATTGTGTCAATCGAACGACCTCGAAATAAAGATAAAATGTATTGAAAAATTAAATTCAAATTATAAAGTAGT
TTAAATTGTTTGTTTTATTTTATTCTTAAATATGTAATTACCTTTATTTGTAAAGAAGTTTTTCCCTGTAGCTTTA
ACAAGATGCGATTAATGGTGTACCTATATTTAACGTAATTATTATAAAGGTTCTATAAAAGTAAGGATCTTATAA
GATAGATTTATACACACATTTGTAATTATAATATGCTTGACCTTCTGGATATACAATGTATCAAAGTGCCAAAGC
CTCAAAGCCTGTATCAAAGTCGGTTGTCCCTTAAGAACAAAATAGTAAGAGGCGATAGCAACTATTTCATCTA
ATCGTTTTTGTCGCCTGTAAAACTAAGTTGTAACGAAAGCATCGCAAAAACATTGAACATGTCAGACAAATAT
TACTGATCATAGCAAAACTGAGGCAAGCTTAGGAGAGCTATGTCTCAAAATATGTACAACATACAAGTTACAT
ATGATCTCAGTGGCGTGCACTTGGAGAGGCCTATGTCCAGCAGTGGACTGCGATAGGCTGATGATGATGATG
ATGATGATAACAAGTTACATAGTTAAAAACATCGGACCTGTAACTGCATCTTGTACAGTAAACTGCACATCAG
TGGTAACTGTCATGCACCTTAACTCAATAGTAATAAGTATGATTTTCCTATAAAACTCCACTGACCAAAGGGTG
ACTGTACGATTGGGTAGACACACAAGAAAGTATTGATGTTATTTTAGAATATTCTTGTAGTGAAAATAATGTG
AAGATTACCTATGCCTAGAAGTCAATGTATTTATACTACTTAAGAGTAAAACTGATTCACCAAATATATACACCA
TAGTAGGAAATGCACCATTAGGTACAAGAAAATGAATGACTACCTATGTCTTAAGGACACATACGCATAGTTC
AATGTCCAAAAATAACATAAGTAACAGAGAGTTTTTGCGACTGCCAAAGCAAGTTTAGATACTTCCAGTATTT
CAGTATTTGGCGCTTATTATATCGTTTTTGCGTAATTATAAGTTAGTCATAATATCGTAAAAAGTCTAGATCCAA
AAATTGTATGTTAACAATTTTTTGTTCACGTACGTTAGCACTGCATGCAAATAAGTCTGATGACATGCTGAATA
GAATTCCGCAATCGCTAATGTACTTTTAATTCCTCAAAATATCCTTATTTGAAGTAGGCTTTTGTAATAAACGAT
AATATCATTAGATTATTTTGTACAATAAAAGATAATAAGGCTTTGTGTCATACTATTTTATGGAAGAACCCTAAA
CTTGAATGAGAAAGCAAAACTCACCACGGTCAAAACGTTATCTTAACTGCTCATTACTAATAATATGCCAGAT
AGGATTATTGACACGCCTCTATAAATATAGATTACAGTTAATTTGTGATCAGTTGTGTTAAACTTGAACTAGAA
GTAACATTCAAA
>HaOG200089 CYP6AE14
TTAAGTAGGTACTTTAATTTAGAATTTTAGTCCGTGATTATATTTCGTCGTGTTTTTTTTTTGGGACGCTCAATA
GGTAGTCCGCTCTGGGTGCCACCCGATGCTACGCCGCCACTATTGTGGTGACCGCTGTAATTACAACTGGATT

ACTAATATGGCCATAGCATTAAATATAATTAATTAAAATATATTTAATTTAATAAATTAGACGACAGCGCATTAAT
GTTAATGTAAAAATGCCTTTGGGTTTAACAATAAAGCTTGTTGCGCATGATGTTTTGGAGTAGCTACACGAAC
AGTAAAAATAAACATATTGATTGTTAAAGTGTCAAAAACTGAATATATTCCTTTTTCTTCACCTTTGAAATGTA
GTTTACAAATAAACGTAGATTTTTTTTTATTAAATTTGAGGTGTAAGAGCCTGTGTAAGACCATTTACAGCACT
TATTAGTCGAGTAAGAGCATTCTATCTGCTGTAATAGAACTAAAATGGCGAATAAAAGCAATCCGGCTGCTATT
AAAGCACTATTTATCTGTATAATAGCAAATCGATTGCGTTTATTGAACTAAATATTATTATCTTATATTACGCATTT
TGGTTGCTCTTAAAACACTCCTAGTGTTTTCTACCACTTTTACACAAATCTTACAGCACGTTGTAGTTGCTTAC
TCAGCGCTTATTCCACTTTAGACTTTAGTCTGAATAATTTGCTCCAGTTTTAAGAATACCGGCCGTTTATGTTG
TTGGTGAAGACCTTCGTTGTTTACAAACTACAGAGACAGCATAAAAAAAGGTAAAGTAAAGTACCTACTTCC
AAACAAAAGGGGTTACAGCTTTGTCTTTACATAGCTGAAAAATCTGTAACTATGGATACCCTACTGATAGTAA
ATTTATTATTCATTTCATTATTCATTATTTATGTTGATTGTATCTAAAAAAACAATAATAATACCTACTTGTTTTACT
TAAATGATAATCATTTGTAATATACCTACATAATCTTATCATAGTCTGCGTTTATTTTACATCGAGTACACGCGCC
CAAAAAACGCCAATCATAGCGTGACAACAATTCATGTAAAACTGTGGACATCCCAACCAAAAACAACTAGTT
TTACACAAGTTTTTTTCTGTATCATATTCTACTAATTATATCGAAATCGCGGTGGCCTAGTGGGTAAAGAACCA
ACCTCTCAAGTTTGAGGGCGCGGGTTCAATTCCAGGTCGGGCTAGTACCAATGCCACTTTTCTAAGTTTGTA
TGTACTTTCTAAGTATATCTTAGACACCAATGACTGTGTTTCGGATGGCACGTTAAACTGTAGGTCCCGGCTG
TCATTTAACATCCTTGACAGTCGTTACGGGTAGTCAGAAGCCAGTAAGTCTGACACCAGTCTAACCAAGGGG
TATTGGGTTGCTGCGGGTTGTTCGAAAGAGATACCGCGGCCCTGGTACTAAAAGGCCTATGTATGACGGAAC
ACGACGGTTTTTAGTCAGTAAGAGTCTGACACTCCCTTACCGCTGCTAACCCACAGCGGGAGGGGTCATTT
GATGATTTTTGACGTCGGAAAAAAAAGGGGTATTGGGTTGCCATTACAATTATTGATTTCTGATACTTACGCG
AATATTTTATTATTTTACATTTAAATAAAACTACTGTTACGGATTTTATCGCGGTTATATTAATATAATTAAATCCC
GACGATAGTTAAAATCCGTAAATGTAGTTTTATTTAAACGATATCAATATTATTGTTTTCTTAACATAAGTTTTAC
TAAATAAAATAAAATGCTTATTAAAATAATAATCACATTCACATATATATTTACTAAACAATATAAAAATAAACCA
CATTGCAGTAACAAGATAAGTCCTCATGAATTAGAACGCGTGGCCACGCTAACGGACATACCACCGAAATCA
TTATGATTAAATCTTCGACAAAACTGTATAAAGCCAAACACGGCACCTGCGACATACAGTTACAACGAAGCTT
CAC
>HaOG200213 GSTD1d
TGTTTGTTATTATAATATTTCGCTAAAGATACCTGCTCCAGCCGGGCTCCAACTTGGGCCGCGTAAACACGGT
TTTGTCAAGAAAACCCTGTACAAGAGTCGACAGAGTTTCACTAGATACCCGCACACCATATTCCGTGCAATAT
TGCATGACAATATTGGCGGCATTGTCGCCACAGCGAAGAAAATCCTTCACCATTTCATTATAAGCTACATCAC
GTTGTTGATTCATAATGAGATAGTTTAAATAAATTAATAAACTTCGTTTCTCTGCCACAAGATTCAGGAATTTT
CAATTCAAAACGACAGTCGACAGACCAACCAAAATGTCAAATGAAAATTATTTTTTTAATGCTATGTTGCCAT
TCCAAGATATTATTTATTACCTTCCTACATCATATCATGTAGTTTGCTTTGGTAGAAATAACTTGGGGGAGAAA
AACTACAATTAAGTATTTTTTTAGTGTTGTATGTATTAAATACGTTATATATGTTATGTATTTTGTATATTTTAAATT
ATTTTGTGTTTTATATTTATGCTTGCTCGCTGATGAGATTTGCCCACACTCGCAGCTCTTTAATTTTGAGTAAAT
AGAGGTTCCCCACTACTTTTGTTTTTACACTTATAGCTATGCTTCAAGGTAATGTACTTTGAATAAAGATAAAT
GAAATAATATAATAATGTAGGCCCATTAAATAATGTAAATTTTTTTATGTTCTTATTACTTGCCTTGATGACATAA
CTTTTCCACAAGTAAAACATTGATGTTTCCGATTCTTCTTAGTTTTTGGTTTTGTACTATTCTCTATTTTAATATC
TAGTTCTATTTTATTATCTAGATCATTTGCTTGTTCCTCCTGTATACTATGATCTATACTTTCATCAATAAATAACAC
TTTATCTTCACTTTTGGGCGATAATGTCTCATTATCAGAGAAATTTTCATTTTCTATGATCATGTCCTTACTGTCT
AGCAAAAAACTTTTTTCCCTACAACTGTTTTCTAGTAAGGCTCTCAGCTTAAGATCAGAAGTTTCACAGAGTT
CAATAAATTCTATACAATCATCCAGTTTTTGAACACATTTTGAACATATGGTATCCGGAAGGCCATCGTCTGGT
GCTATCTACAATATAAAATAACTGATTATTTCACAAGAACAATATTTTTTTATTGATAAAGACCTTGACTGGACAT
CATTGAAGATTTGTCCTGCATGTGGGTTAGCGATGAAGCAAGTTCTCCATAGATGTTAATGAGCTTATATTTTA

TATTGGAGATTTGAAGTAGTACAGAATAGAATAGAATCTACAAAAGCCATAACCAGCATTATATAGATTATCTG
AACGGCTTCTGCGACCCAAGTTTAGAGAAAATTATTCTGATAAACCAAAATAATGTTTTACTCACTTTTATTTT
TGCTATAGACATTAACTTGGTGGATAAAGTAAGGAAACTTGTCTTTTTGAATAAATTTACTAAGAAGTCACGA
TTGAAACAAACTCTACAAATTGCGGTCATTTTCAATGCGTTGAACCAATAACTTTAGGTTATTAAACAAGCTT
GCATTCATAAATAAAATGTAAACACAAAAATGTAAACACGAAGTTGCACACAGCATGACAGTGACAGATTGG
ACATGAGTAATGAGCAATGGTAATGAGTAGTTTCCAAGACATATTTTTCTATGGCAACCAATGGCAATAACCA
AGAACTCTTATCTTACGGAAGAGGACAAAACAAAATGAAAAAGAAAATGAGAAAAGTTAAAGAGTATTTTT
AAATACATGCGCTAACTTTTCCATCCGCACTCCAAAATTTTTGACTTTTCCTATGCAACAACAAAACATAATCC
AGCTGTTTCCTATTTCATAAAGCACAGCACTGTCAATGTTTTGAATCTTTTGAAGTCACTGAATGTCAATTCAC
AACAATGTCGTCAGCGAACTTTCGAATTTGAACTAGTTTTGCAGTGTCATTCATTAGCTTAAAACTTGTGCC
>HaOG200215 GSTD1s
TATTTATTCAACAAATGATTTAAGTAGCTGTATATCTGTTATGATGAATCATCTTCTAGAATTGATAATTAATTTTT
GTCTGTCACTATTGGTGTCACATGATTCGATTAGGATTTTATTTATCTACTTAATCCATAGTCCTAATAATATCCTC
CTATCCTACTTATAATTATAAATATGAAATGTGACAATGCAGTCGACTCTTGTTAATTCAAACCTGCGATATTTC
GAACCTCTCGTTAATTCAAAGTTATCACGAGTTCCCTACAAAATCTCTTTATATTTCGAACTAAATCAATACATT
TTTATGCACTTGATAATTCGAACGAAAAATGCATTGCCATATAACGAAACGACGCGTTAATTCGAATTCTTAGT
CGTCCAACACTCGAGAATTCAAAGTTAAAGTAAAAGCAGAGAGAAAGAGGCGGAAAGATTGTAAGTCAGA
GTCAGACAGCAGTCTGAGTAGCAGCAATCTGTTTTGAAGCAGTATGACGGAGCAGTTTCGAAATATTAATTA
ATTCACATTATGAATACATGTATGCACAAAAATGTGTCTTTAAACTTTTCACATTGTTATAAAATAGCAGTTAAC
AGATAATAATTGACCAACACTTAATAATTCGAAGTTTTATTTATTTTCTCTCACAAATTTCGAACCATTTAATATT
TCAAAAACTCGATAATTCGTAATATTTTAAGAGTCCCTCGAGTTTCGAATTAACGAGAGTCGACTGTATATGGA
TGTATGTTTGTTATTCTTTCACGCTAAAACGGCTGGACCGATTTCGTTGAAATTTCGTATGTAGATAGGATACC
CTGGACTCACTCATAGGTTACTTTTTATCAATACACCATGCAGGTAAAGCCGTGATCAGAAGCTAGTATAAAAT
ATAGGTAATTAAATAGTAGGTACCAGAATATTCAATATTTATTTTATTAGATAATATTTATTTAAACATATAAACAA
AATATAATCACGTAGGCAGCTGCATATGAAGCCGACCCCAACATAGTTGGGAAATAATGGGAAAAGACTAGC
CAGATAATGATGGTGTAAGTCTCAGATCAAAGAAATAGGTGCAAGTTATTAAATAATATAACTGAACCAAAAG
ATGTAAATTGCTACGGCAAAAACTTCTTATGAAGATTTCTATTATGTACATAGATTAAAATAAAACCTAAGATG
AATCATTGACGAAAAAAAAAAACGAAAATGACAATAATTTCGCAATACTAAAATTGTACCTATGCAAAGTAGGT
ACATTATTTTGTCTAGTCATGGTGTATCAAATGTACATTTGATACACCATGACTAGACAAAATAATGTACAGTCA
TGCACACAAGTTTTGTACCCATTATCGCGTTGGTACAGGATCTTGATAACAAAAAATCTCGCTAGGAGACAA
GATTGAACATGTTCTACCTACACAAATAGTACGATGATAACAACCGTTTATAAATAAATAAGTCGGCCATGGCC
TTGGTTATACAGTGATTGACGAGACGTCTTGTGATACAGTCGCATACAGTCGTTTTGTATTTCAATTTAAAATA
ATAAACAATCAAACGTGAGTTTTTTTTATTTTTATTATGATTACTTACATAAAGTTTGATTTTATTCATTGGCACG
TCGACATGTCATTATAATGCTATAATATTTTATATTCCCCAACATATTTTGTTATTAATGTAATCTTCATTAAAATAT
GTTCCAAATCAAATTATAATAAAATACGTCTACTTAAATGGATTACCAAATAAGAATAAGAATTTCATAATATTA
AACACAAAGAAAAAAAAACTTATAAAAAATACATCCATAAAAATAAATAAACATTGATAAGGGGTATCATTAT
ACTGTTTATCCAATTTAAATTCATATTTGTAATTCTTTAGTATTTGGCCAAGTTCTGACTTAATAATAATATGGCA
ATAAAACAGGTTTTTTTCAGATTTTTTTATTTTTATTTCAGTATATTTTCATATGTTTTTTTCA
>HaOG200252 GSTD1p
TGCCTACCTGTAATTACTTTATTAAAGACAAAGATAATAAAATATAATGTAAATTTCTTGGTGTTTCTTTTTTATC
TTACTTTCTATGTATTATATTATCGTATTCCTTTCAAACCTCTTTTTACCTAAAAAATCATCAGTAGAAAGACCAC
GAAAAAAGGGGTACCTACTGTGGTACCCTAAGATGGAAACTGGCTTGGATTATACCGCAGGTTTTCATTCTA
TTTCTTCAAAAAATATAATATCACCCAAAGTTCAAATCGTGTTCCTATGTATAGGTCTGTAACTACTTGTCTACT
GCTTCCACAGGCCATTATTCACAACTTTAGTCTACTTCGGCTGCGGCTGTTGTCGAAAAAGAGCAACTAGCT

GCTCAGGAATTAATTTTCGCACACAAGGGTATGCGTAGACACAGGTACACTATTGTAATTTGTCCAATGGGAC
TTCATAGGCTCCGGCCATGCTCCGGCTTTTGTAAAAGCTTCCTTAAACTTATAAAGCTATTTCACCTGACACG
GGATTCAAACCTGAGACCACGTGCATAGAATTCGCGACTTGTAACACTTTTTGGAGGCGGTTATCCTCATGG
ACTTCCTCGTCCTGGGGAGAGGCGGAGGGGTGTGCCGGACTGGTTTTATAGTCTTTGGCCGGACTGTTACC
GACTAAAACCCCTGTGTCCTCTTTGCACGTGGGCGCGGGGCCGCGGGAATCAGATTCTTCCGCAGCCCCAC
ACTAGTGCTGGCCCTCGCACGGGCCTCGTCTCTTGCACGGGGTAGTGAAGTGTTCGCCTACCCCGTGACGA
CTTGTAACACTAGACCAACTTAACTCATTTCGTCATTTCGAGGAAGAAGTCCTGCGGAAAATTGTGGAACTAT
ATATTTTTGTACGTAGGTATTTACTGAGCTATCGCATACACTAGCGCTGCGTCTTCCGTACTTCTTTCGTGAAAT
GGATTTTATCACAATGTCTCATTAGCCCCGGTACACGGAAATGGAATTCGAATAGATACATACCATACCATACC
ATACAATTACTCTCGTACTTATCCAAAAAGTTTGCGTCTGGTGGGTGCGGATATTAGGGTGGGTTTTTGGAAC
ACAAAACCCATAGATTAGTTGAAAAGGTTTAGTGCAATTGCAGCATCATTACATCATAATATTTTTACACCTAC
GTGTACCTAGATAGACGTAATAAACAAAAAAGTCAACACTGTTATGATAAACCGACAAAATTATCAGGTTTTA
TTAGCAGGGTACACGACGCGACACATGATTCGTGTATTCAGGCTTGAAGGCGTTATCGAGGGTCTTTTTAAA
TCGGTTGGGTTATAAGGGTAATAACATCGAGTCCAAGGCTAGCCGCTTTAGTATTATTTTCTCTGTTAACAGC
GAATCACGGTGTGAGTTAGGTAAGTGATAATAACAAAATATAATGTTTCAATAAGTCTTAAAAAAATATTGAAT
AATTTAGGTTTAAATGTAGATCCATATTGTTAAGCGATTAGATTTTCATTTATTAAAATAAAAATATCTATGTAGG
TACCTACCTACCTCCAGCTCCATAAAAAATGTAGAGAGATATTGAAAGCCGGTTTCAGACTCCAACTGGGAG
TGAGTTTCTGGAAAATCCACCTATCAGTGTTTAAATCTATTTAGCGTTATGTTTAAATTATTTATTTATTTAAAAA
GGTTTTAAAGTGTTTTCTCCAAAACATGCAGCATGTAAGACCTACATGATAGATATAGCAGGAGCTTTGGCCA
TTTCAAAATAAAAATATTTATAACAATGGGTAAAAAAAAATCACCAACATACTCTGTAATAAATAATTAGAATT
GAATTGAAATGACTTGCTCTGAATGTATCACTTATCAGCCTGCACTCAGCTAAAAATAAAGTGATAATTTAACC
AACAGTAACCAAGCTAAAAGTGATATGTCATGCTTCTCTCTGCGGGTTTAATTAAAACTTCGTGGTTTTTCTTT
TCGCAGAACAATCCAGA
>HaOG200253 GSTDIc
AAGTGTAGCAGCATAATGCAATATTGTATTTAGAGGTACAGAAGTAAGAGAGCATTTGCTCGCAGTATAATAC
TCGCATTTTTGTAAGAAATTGTGGTAGAAATAAAATTATTTATATTTGAAGCAAGTACTTTTATTGGTGCCTTAT
AATCCTTATAATTTTTAACTTAATCATAAATTTAAAGCGGTGTAACGTGATAAATGAACATGTCATACTGTGAAT
CAGTAGACTAGTACTTTGCAGTAGGCTCCCTCATTCGTTTGTACAAAACTTCCAAACACACACTTCAAAATGA
TTATATAGTTTTCTTACGAAAATCACAATTGTTTGTTTGGACGCTATAGGTAATCCTAGGATGCGACGAATGAA
AGACAATTGCATGCGGCAGAGATGAGAATGTTAAGAGGTATGTGTGGAGTTACGCGAATGGATAGAGTAAG
GAATGAGTATATAAGAGGAAGTTTGAAAGTGGCGCCGGTATTAGAAAAACTGCGTGGAAACCGGCTGTCGT
GGTATGGGCATGTGATGCGGAGGAATGAGAGTCATGTTGTGAGGATGGTGATGAGTATGAACGTGGACGG
ATATAGTGGAAGAGGAAGACCAAAGAAACGATGGATGGATTGTGTGAAAATAGATATGGTAAGAAAGAATG
TTACTTGTGAGATGACGGCAGATAGACGAGTATGGATGATGATGCTTATGAATGTCCGATTTGAAAAAATATT
CAGTGTCAGACAGCATATATTTGTCATGGAAGGCTACTGTACTTACTTTACTTTAACTTAAAGACCAGTAGGTA
AGTACTTAACTAACGTCCTATAGTCCTATATACAGGTGATCGTACATTGTTCCACGTTATATTATAGGTAAGAAC
TTTAGCAAGTCGTGTTCAGGCGCCGAGCAGTCTCGTCACGGCATCTTGCGTTATGAGCACAACATGTGTGCC
TGGAAACTTGACTTAAAAGTCTGAAATTCACCCAACTAGCTCTAGGAACCGAGATAAATTTTTGTGGGCCCA
TCGCAGTCAAATACGTCGTAGAAAGGGGTATTGTGGTACCCAGATGGAGACTGGCTACTTTTTACCCGAATT
CTGGGAGTAGTTTTCCAGGATACTGATATTATACCGCAGGTTTTTATTCTATTTTATATCACCCAAAGTTCAATT
CGTGAGTTCTTAAGTGGCCATTCATCATAACATAAAGTGTTTTTAAATAAATAAATAACTTTCAGCTGCTGCGG
CCGTGATGATGATTTACGATTGCAATATGATTGTAGAGAAAACTACTGTATCTACCATATGACAGACCAATCGC
AAACCAATGAATACGATTGGTCATATATTAGTAGCAGATTGCCAGCTAAGGTTTAAACTTTAAACTTCTATTGC
TATAGGTACCATTTTTATTTAATTTTGACATTATAAGCAGAATCGGGCCCCTGATACAAATATTGAACCTGAGA

CCACGTGCATAGAATTCGCGACTTGTTAGAATAGGTAGACCAACTTAACTCGGGTAGGTAGTCACATTAATCC
TATCTAGGGCTGCCTCTCGAATTTCGCCAAACCAGGACAAACATTAAAAAAACCCGGACATTTGTTTTCACT
GGGAAAAAGTTGGGTAACTATAGGAAAAGCTTTGGCCATTTCAAAATATCTTTTTTTATAACAATGAGTTCCT
TGCGTAAGACACCTGAAATGGAAACATATATAAATGGAGGTAATTTGTTGACTAATATTTTTAAGGAAGTAAA
AAAAATCACCAACATGACTTGATAGAATTTGCTCATGACATCAACCAGAATGACTTGCTCCGAATATATCAGTT
GTCAGCCTGCACTAACAAAGATAAAAGTGCTATGTCATGCTTCTCTCTGCGGGTTTAAATAAAACGACGTTGT
TTTTCTTTTCGCAGAACAATTCAGA
>HaOG200231 GSTD1k
TTTTTATTTTTCCTGCCATGACGGGTTTCGCTTTTGTTTTACTTCACTTTTGGGAAAGTCAAATATTGGTATTG
CCCGCGGTTCGTCGCGCGATAAAAATCAATTAAAAACCCATGAAAACCTTGCTAGAAGTGCTGACTGTTAGT
GCCTCGATTACCCGGACTCGTGAAAAGACTATACTGAACAAGTGTGGAGAAAGTGTCCCGCTGGCCGGCCG
GCATCGAGTGCCCCGTCGTCGGCGGACTTCTTTCCCGCCCACTTTCCTGTTCCCAGGTAAGTCTGTCTTCTTC
ACTCATACTGAGGGTCACAGGGTTTCAACGGGGACTTCAACGGAGATTGCAGTAACGCCGGCAACTTCTTC
AGTGAGTTCTGTCCACATAGAAAAAGCACATAGGTAGGTATGCTCATAGATTATACGGAGTGTGCATCCCTAT
CTAGACCTTCCCACTACTTATTGCAGGAGTGCTTTGGTGCAGAACTTTCTAGCATCGTCTCACATTTCGAAGA
AAAAGTTAGGAGAAAAGGCAAAAGGAAAAGTCAGTGTCTTTGTCAGTATGTATGAAATATGCCAACAGCTT
CGTTGGAAATACTTAAATAAAAGATCAAGAACTAAAAAAATTGCAGCATGCTTTTTTCGACCAATTTTTGTTC
CCTGATCCTCAAACCGTTTCGATCGGCCACTTGGAATAAATTAAATACAAGAAAATCTTCAAGTACCTATACA
GCGTAAAACAATAGTAAACTACGTACTAATATACTTACTAATATTAATGACAGAACCCATGGTTTTTGGTCAGG
AAGCATTACATTGCCAGTAATCTCAGATTGTGGAGATTATTGTAATATCATCGATTAAAATAGACAAGGAAAA
AGTTATAAATAATTGTTTCGATGACAAGCAGTTTAAGTTCCGAATCATATGTACAAAGATTACGGTACTAAAAA
CAATCATGTGATGCGAATTATCGTCCACTAACAGCTGGTAACTCCATATTAGGTACTTATTATGCAATGGCCACT
AGCAAAGACGATACGATGACATGCCAAACTTTATCAGTTGTTTATTAGTTTGGTTGGTTTAGAGAAAATATAA
GAGATTATTATGCCGTATCTATTTATATACGCGGTTGGCTCGATAACGGTAACAACACGTCCCACACGAACCG
GCTGCAGCGGCCTGTGGCACTTCGGTCCACAACTTGAAGCGATTCGATCGTCTCTGCGAATAAGCGTGCTC
GGATTTTCATTTGAACGAAATCTAAAAGGTACGCTATTGTTATTTTTAATTTTGGTGTATTTTTATTAACCATGA
TAATATTAATTCTTTAAATTAGAAAAATGGTTTAGTTTGTGTGATTACCGGTTTGTTTTGACAACAAACGAGTT
CAAGGATGACTAACATTCGAGATGTAATTTTTAAATTATGGAGTCTAAAGTTTACATGTATACGACGATGTACT
TTTAGAACTTTGTACGGGTTTACCTTCGCTTTCCAATTTCCTATTCGGATAAACAAAATGTTCGTGTTTGAATC
AATAGAGTTAACTTCCTGTAGTACAAAGCTTGTTTGATTGAAAACGACGATGCATCCGTCCCCTCTTATCTACA
GTATTGAACTACTAACAATAGCTTGTTACATCCGCTGTCTGTTTATCAATACCTACACTTTTATGCATAATACTTT
TTCGTATGGGAATTACTACAGGATTTTTTTTACCAGATATTTTGACAAAAATTTTGTATGTTCCCCTGGTTTCC
GACCTCAGATCGTAAATTATTTTTCCAACTTTTGGCGTATTTTCTTTCCTTTGCCCTTTTGGGTTTCATTTTATT
TCAAATCACTTTTTTTAATAGATATTCTCTGGCCTACGTATGGCTATGAGGATGTATTTATTGGAATCGTAAAAA
AATCAATAGCATTCAACTTCATCATCATCATTTTATTTTTAAGGGTACGAAATTATATTTTAAAAATTTAAAACCT
TCTTTCAGAATAACA
>HaOG200232 GSTD1L
AAAAAGCTCAAGATAATCTCAGCAGTCTTATGTTGTAGGCTTGCATATTATTTTATAAGTTATTTAAGTTTTTTT
TATTATTTTGAGTGTTTCTTTTACAAATATTTTGCGTTGCATTTGTTAAAGTAATATATGAATGAATTGATTTAAA
ATATTTACAACATTATTAAATGATACTGATTAGATAAATGCTATTGTTTTATTTATTAAACATTCAAACAATACAA
CTAGAACATAAAAAGGTAAACGGAATGATATTGCAGCTACAAACTATTTCTTCAGTTTTTCTAAATTGAAAATA
TTATAATCAAAAATGATTACTCTCCACCCACACTTTCCGTCAAATAGATTGGCCCATGTATTTAACCTGATATATT
TTTGCGCTGTTTTAAAGCGCCTGTATAAAAATAAAGAGCAACAATTCTTCCAAAGTCAAGTAACCATTTGTCT
ATAAAGCTGACGGAATTGTTGATTTAAACGCGCTAATATCAGTCTTTGAAATGAGTTGAAAATTATATCAATGT

TAGATAGCCCATTTCAAAATTTAACCAAGCTGGGCAAGAACTTAGGGTTTGCACAGTAGATTATACGTAGTGC
GCATTCATATCTAGTGTCTTATACGATCATCTCTGGAGTGCTTTGGTTCAGAATTTCCTAGCATAGTCTCACGCT
TTGAGAGGATTGAAGGTGTCAAGGACGACTTAAGACGAAATGTGTAGTTCCATTGGCCATTAGAGACTAAG
AAAAAAATACGCTAAAAATAATGAATAAAATAATATCTATAATATAAGTATGCTTAAGTATGCCAATTCACTGTT
CCCCGATCGTCAAACCGTTTCAATTGGAATAATTTGAATACATTATAAACACTGGGACAAGATAAACTAAGTG
CTAATATTATCCCCTCAGACGTCATTAAAAGATAAGTGTAAGGTGTTCCAATTAAACAGTGTTTAAATACTCTTT
CGGTTGTAAAAGGTTTTGAGAACTGGCAACGCGGAAAATAAACGTCAGACGGGATGCCGGATTATTGAAC
AAAAAAAAAAGAAAAGGCGGGACTTGGCGTGTTGAGTTTGGGATTCCGTTAGCGATATTATTTGTTACAATT
TTGCAATTGATTTTAATATAATACGGGATGGTAATGCTAGTATTAACCGCTAAATATTATAACCTGTAAAAGTAA
ACTGCTGGTATTTCTTTTATGAGAGCCCCCTGGATATTTCTACCTCTGCAACCTGAAGGAGATTCTTACACTAG
TTATTTCCTGCAACCTCACCCTAGTATTGAGAAACATTATTGATTCCAAAACAAAACACAATGATATGGCAGAA
CCAGTTTATCTCATTATTGCGTAACAGTTGATAAAAGAACGGGTTAGTCTGGTTTACCCGTATAGATAAGTTTA
CCGTCAAAATATGTTTGCAAACTTGTTCTTGGAACATCTCAGTTGTCATCACCAGTCGAAGACGACGTGTCTT
GAAGATTATTTAATAAAAACACAAGTAAGTATATATTATGAAAATTTATCTAATGCATGTATCATGTTTTTTTCG
ATGGAAAATAGCAGTAACAAACAATATTTCGACCAGCCATAATTTTACGTGAATCGTAAAAAGTTGAGGGTA
AAAGGGTAGGTCGAAGCTAGGGTCTCTTTAATAGGCTTAAGACGTCGTTTATAAGCAGTTTTTATATTTTTCC
AATGAAGACCAGGAAGATAAGAACTGACACAACAGAATCAGTTTTCTGTTTTACAGGCCTAGGATCAAAAT
GTTTTAGTTGAAGTAGTGTTGGTGACACGAGTAAAAAATAATAGTTGGACGTTGAGTGACAGATGTTCAATC
AACGTTAAAAAAAGGTATATGTATAGGTACATGATTTTCCATATAAATTAACAAAAGAAACCCTATTTTCAGGT
TCAAA

>HaOG200244   GSTS1f

ATAAGCCATTCAGCTATTTTTGGCCAATTGACAACGAGGGAAGAACATGTTTTGTGGCTACTTTTACAGATTG
CTAGTTTGAGCTAGGTTCCCGTGTGAATACTTTTTGGTGCTTTTTGTCAAACTGTCCTTTAGACGCAATTCGA
TTCATAGGAGTTAGGAACCAAAGTCTTGAGCGAAAGACATGCCTTCCAATTTCTGGATACAAAAGGCTAGTG
ACTGTGCAAGACTATTAAAAACCTAAAACATGTCTAGTTAAAATCGCTTACACCAATATAACCACCTTACTTAC
TGATTATGGAACGATTTAGAGTTAGCTAAGTAGTTTTTTTTTGCAAAATATAAGTACGTTATCAATATTATCATC
ATCCAAAATAAGTTATTTATCTGATTGCATCGATTTATCAGTGTCACACTATGGGTTTACCATGCTTTTGTCACG
CCACGACCTTGCCTGTCAATGTCCCACACAGCTGTAAGCTGTAAGCAGTAAGTAGGTATGTAGTAACCTGTTA
TCATGTTCGTGTGACCGTTATCTTAAATTGAATGTTTCTACTGAAACTGTTATCAGCTGGTGACTACAATATTTT
CATAATAATATTATCGATTGATATGAAGATTTTAAAAATATGTGTGAGTATAATGGGCGTGACTAGCAAGGCAC
AATAGTATGCCAACGTTGTAGTAAGTCTGCAATAACTCTTAACTCTCGACCCCGAGTTACTTTGCGGGGAACT
CAACAAGACAGACAAAGTGAAATATTAAACTGAATATTATTTACTTAAAATAGCTATGATCTGATAATAATTTC
AATAGTTTTATGAAAATTTGTACAGTTGGAAATAAAGCCTGCTTGCCAGATAGCACTTTTCTAACCAGCTGAA
TGAATTTGAACCGTTGTAGTTAAAAAGGTTACTGTTGTTTTTAACATCGATTATTGATTGAGGATTTTCAAGA
CATTGAGGATGTTCAAGGATATATTTACCGTGAAAACCAGGTACCTACCTTCATTAATAAATCGACGACAGTG
AAATACATTTTACACCAATTTGGTTGCTATGACAATGCTATACCACCAACAAACATCTACTTTTCTATCTACAAA
TAATTACCTTATATCGTTGAACTTCTATGAAAAGTCTTGAATAACTCAAACAGTGCTTACTGCAAATATAGCTTT
TCATTATTGGAAGGAAAAAATATGTTTGTAACATAAAAATACTTTTGAATAGATCGAGCTTAAATTCTGCGCCA
ATCTTTTTGGGTATGCAAAAACTTATTTTACTCAAATAAAACGAGTTTAGAAATATCGATTATGAAGTGGGTAG
TCTTTTAAAACCTATAGCGAGTCAATAATTATTTTACTTTTTTATAACGTGCGGTTCGACTCATTAACTTAATGTT
CTATGAAGTTTATTCAAAAAGCTCGTTTGGAAACAAATCATGTAGCTAGTAATTTTTACATTGAGTCACGCTCT
GCTAGTCCCCACTAAGAATGACTGACTGACTGACTTTTTCACCATGACTTGCATTTTTTGAACATGACAGTCT
GTTTTGGGAAGTTCCGCTGGTATTTATCTGCGAGTGGGGTCTGCCTATCTTTATTACGTTCAAGACTGTTGCC
GATACAACTCGTGTGTGGGACGAACTAGATAATACAAAACGTATGTATTTTTTAAATTGATAATTTCTGTTGAA

GTTGCTGATTTGTTTCATTGTTGTTGTTGATAATTTATTATTCAAAAGGGTGTATTTTTCTATTGATTTAGGTTTT
TCACAATATATTTGATAAATAAATTAGGTAGGTAACAGTTTGAGTTTCTAGTATAAGTTAAGTCTAGTGTAATTT
AATTTAATTTGATTTTTAATTTTTAGTATTAAAACCTGGTGTTATAACAAAAAGTTAGTTTCATCGATATCGCAA
AAAGGAATAAAACAAGTAAATAAATGGGCCCAGCCCACCAGAAAATTTATAAATATTTGTTTGATCTGCAGA
ACC
>HaOG200266 UGT40L1
AATTTTCAAAGAGATAGCAAAGACAACAGACAGAACTTTATAGTCAACGTCACTCACAGAGAACAAACCATT
TGATTGAAAGGCCTTTTGTTGTTCTGCTATGCCTATTTGACTATTGGTGTCAAACAGCATTGCTGAGTCCAATT
TTGCATAGAAACCTGATGGAGATATCAGTCTATCACACTGTAAGAAAGACAAAAATTTTTGAGTGTGTTTGTG
CTAAAATAACAGACTTTGAGTGGAAGCCCATCGGGTTTCATACTACATATAAAAACCGCGAAAGTTTGCGTGT
ACGACTTCATGCAAAACCAACAGATTTTAATGAAACTTAACAGCGATAATTACTCGTAGCTGAAACATCAGGC
TACTTTTTGTCCCAGTGCATTTTTGCAAACATTTTTTTTACTGGCTTAAACATTACAACATTAGTTCAAAATCCA
ATGACTTCCAAAAACAATTAATCTTACGATTTTTTTTTTCTTTATCACTCAATAATTAGGTAACAAACTGTTATG
ACGTAAGTAGGTAACTCCAATTAGTTTTTTATATGTTTGTGTCATGTTTCATCTCGTAAATTATCAACAGTGGGC
TACTAACGATATTTCGTGTTGCTCTTGTATTCAGTTCAGTTAGAACCATCGACAGTGATCCATGTTGCGTGATG
ACTTGTAAAATAATAAGCAAAATAAACCTTACGTGATTAGTAATTGAGTTCAAAATTAAGTGATTTATTTGTGA
GCGTGTTAAATAGTATAAAGTAAGTTGAACTTAAAAAGTATTGTATAATTCAATTTTGTAATTTTTTTTTAACTT
AGATGATGCTAGTTTACCGGTAGGTATTATTTGTTATCGTCTTATCTTTCTGCAAACAAAGAAGTTAGAATTCA
CCATAATCTTCAGTAAAATTGTGGTAAAATGTAGACTTACGCTTTTTATGAATTAAATACCTAATTTAATAATTA
ACAAAGTTTACCTTTTATAGTATTAGCACAAATCTGTATTTTTTTAACAATTGTTTCCTGAAACAAAATAAATGA
ATCTATCAAATAAATATTGTATAGTTTATCTGAAGATGAAGGTCAAATGTGTTTACGATAGGAGCAGTGCCGCC
TGGCTGGCTAATTAGTCGAAATCATGTTGAAATAATATGAACCCATGATTTCATAAATCATTGAGCCTATTACAT
CAAAGCGTTCCTATAAAAATGTGTAGAAACAATGATAAGTAATACAACATAAATACTAATTTTGTAGATGACTT
TATCCTAATTTTGTGTTACAAAATAATACAATACGAGAGTTATTTAACAGCTTTCATATAGAATAGCTTTCGTCC
GGTCCCTAGGTTTTCCATTTTGTAACTCGCGCCCCGTATAAAGTACCTATAAACTCCATTCATTCAAAAATGAC
ATAAATAGATATTGTAACAACAATAATTGCGATAATTTATAATCATTACATTTGTTTGTTTGTATATCAATCAAGG
AAGGGTTCAGAACTGATTAAAATGTATGGCAGGTCCACATTAACGACACATTTAAAACATGGTTTCTTATACT
TCTACATCTACACCATGCTAATATTATGCAAATAAAAATAATTGCGTATTTATATCAACCATGAAGGTGATGTAA
GCACATCTTGAATGATTTGTAATGATGACTATGAAATTATAAAACCTTTATGGCCTTACTACAAAAACTTTAAA
CCCTGTTTTACACTTGTCTAATAAAATTTTAGCTGCAAAATGAACCATATGTCAACGTCATAATTTGTCATTTTT
TTAGACAAGGCATAAACTGACGTTTAAAAGTTTTTGTGGTAAGTAAGACGGTTAAGGTCATAAAAATAGCAA
ACTGTATGCAACAAAATATTCTTGCTTTATCACCATAGTTACCAAAATGAAGTATTCCATATCTGTGGTCTATCTA
TACTGATATTATGGTCGCCGAAAAGTTTGTTTTTTTGTTTGAAGGTATAAAAATCATCTTATGTTTTCA
>HaOG200264 UGT40M1
TAAATTGTGAATGCTAAATAAATGCATTTTGGAAATAAATAATCTTTTCTTATGGAGAGGTGCAGGATCATCTT
TCATTACTTAATTAGATCTAGTGTCAGATCTCTGAAATCTAGCTGACGGTCTCTGATGTGAAACTTTGAGAAG
GATTGCCGGGTATCACTCTTAGATATCTATCAGAAAGCTGGCTTGCTACTTACTTTCAGACTCCGGATTGCGTT
GTGAAAATTTCTGAAAACCCCACTTTGTGGATCAGATTTGGGCCTGATCGGAATCGAACCTGGGACCCTGTG
AAGGGCATTCTCTTCAGCTTTATAATATTAGCATGGGTGATCCCACGTTTATAAGCCATATACATACATGCCCAC
GGAGAACAAAATGACGAGCGGATATGTCATAATATAGAATCTCCTAAGAAAGCAGCGTTGCCAAAATGTGG
GTGTTCGGGGAACGAAGAATGCACCATTCCAACCTCACACGGCTTCTTTGGAACCCGCCCCTTTCATAACAC
CAAAAATCGGAAAATGTATCAAGGAACCCGAACGACTCGTTAGTAACGGATCGTTTTGTGCACACCCACCGT
ACGTATTTGACCTAAAAGAAGGCGCCTGACCTACCTGCATTATAGTATCTCCGCTCATCTTTTCTTACTCCGTG
ATGATAAAGTAATAGCATGGTGACTGAATAACTGGAGCAATTCACTTGCTACCAGTAGAGCATCTACCTATATA

ATATAGCTAACACATAAAGTGGTCAGATTCATTGCAGCGTCAATTTAAAACGATTGAAGGTTATAAACTACAC
ACTTTGTCTGTATATTTGCGTCCGAAAAAATAGGAGGGGGACCACAATTTATTACGAACTGACTGTTATTGTA
AGATGACTTGAAAAAGGCTTGATAGGTGGAGATGATTTTGGTTTTCCGTTCATTCATTTTATGAATCCCTAAA
AAATAATAATCATTTTACTTTTTATCGTCCTACTGCTGGGTACAGGCCTCCTCTTACACTGAGAAGGATTGAGC
GTTAATCACCACGTTCAATGTGGGTTGGTGATTTCAGACTTTGTAGCCCAGGTATCCTCAAGATGTTTTCCTT
CATCTTCAAGAAATAATAATTCAATTATTTACTTTTTTTGACGCTTTCCTGGCAAAAATATTGATTTTGATGTATA
TTGCAATTTGTGTGGATTTTAAACGAACTAAAACAGTTCATTCGAGTATTGATTCAAAATGTATATTCCTCTCA
CAGGTTACAACAAAATAGAGTATGTTTTCTTAGCACGATAAATGAGCATTATTTATGCATTACCATTGTTTGAA
AAACTGTTATTAAAAAAACATCTTGGCTGTCGGGCAAAACAAAAAAACCGTCAAGGTAATTGCTGTATCATG
TATTTTTTGGTGAGTTAGTAATTTCCTCTATCCTAAGATTTAACAGACAACTTAAAACATTTATTTGTAGGTAGG
TATATACAATGAGCATGTGACCATCAAAATCATGTCAATAAGATGACTAGACCAAATCTTTCTTGATAATATTAA
TTTGGAACATTTTACCTCCACTCATTATCACAAAACAAATACCCAAATAACTAAAAATATTCATACCTCTAAACT
CTTAAGCGGAGATTCCACTAACATAGTTTGCACACATTTTAAAAATGTGCTCGCGTAATTTGTTACTACTTGCA
AATGCGCGAAGTAGACCAGACCAGAAGCAACATTAATTTGTGTGCGTCACAGTGCCACACGCCGGTGGAAT
CTCGCCTTTATTTCAACGGTACTCAGTCAGATGACATAATATAACTACCTATACCTACATAGATAACTCTACGTAT
AATAAGCCGCTATGGTTCAATCATCTGCGCTCAAAAGGAATCAGGCGCGGGCGCACTAAACACGCGCTTTCA
ATATTTAAAAACAGAACACATTCGATATTCGATATTTAAAATAAGTAAATGAATTCTTCCGCATTTGTTTTATAA
AGACGTGTTTTAA
>HaOG200271 UGT41B1
TTTTTCAAATGTCAAGTCCATAGTACTAGCCATCTTCGAACAAGCAGAGCTTCTCGACAAAATCTGTACAAAC
AATAAATGCGCAATGACTTATATGGTTAAATTCAGAAATGCAAGGTTAATTGGCGAGAACTAATCTCCCCAGC
TTTTGAGAGGCAGCAGTCCAGCAGTGAACGTTTTAGGATATGACAACGATGATACAAGAATGTGTCCTACAA
TTTAATATAGAAAAGATCTCACCGTTATTCTAGTCTACGCAAAACGCGGCTGCTGTGCATAAGTTCTCTTGTTC
CATTCCCTGGTAGGCCTTTGCGGGTTTTAGAAACTTTCACAAAACACCCCAAGCTTGGAAATTGCTGATTGA
TACACCCGTGCATCGGTGAGCACGTTAATATCGGTCCTGCGCCTGATCTCTCTCCGGTGGTGTCGGATTGCCG
TAGGTACCTACCATCGCGCTATGAGAGTGAAGGAGTAGTGAGTGCACCTGTGTCCAAGCAAATGCTTGTGCA
CTATTATATGTATTGTGCAGCTGGCTGATCTCCTTGAACAGCTGTCGTGGATGCCATTAGGTCCCGAGAAATAC
TCGTGGCCACTCGCTCACGTCTGTCACATGAGTACAAAGTTATTGTCATGTCCCTCTACCCGGTAATTATCTCA
GGGGCAGTCTAAAGCATTAATTCAGTTGTGCAATATTACACAAATATTGCCTGAAAATAAGAAATATGGCGCA
ATATAAATGCATATCATGGGAAAAAAGGCTCGGAGGATGATAAATGCATATCACGCAACAGGTGTCATTGTAG
GCATGATGACAGGGTTATAATACAGTGATTCTGATTAGTTCCTGCTTTTTAGATTAGTAAAATTGCAATTTAAC
ACATCTTTAAAAATTATTAACATATAAGGCGTTAAGGTTAATAATTCAACTGGTACGAAAGCTTAGAAATTCAA
ATATAATAGGTATGCTTTTAAAATTTCAAAATATAACAACATAAATATTCCTATTCATTTGAATTGTAAGAACATT
CGGAGTTAAACAAAAACATCAAAAAATACTTTGAGGTAGGCATATTATTTCCGCAATCAATAAGATCACGTCT
CATATCGAATGTTAATTTAGGCAAAACATCAAAAAACATAATTTTTTTATTTATATTTGAATTGCCCGTTACCAG
TTGAAGCATTTACGTTGAGCAGGAATTAACATCACGTTTATTAAATGTACAAATTGAACTCGAAAGTGACGTA
ACACAAAGTTCAAAAATGTTTTATCCTCGTTGTTATTTGTTTATGAGAGAAAAGTAAAAACACGAGTCCATCA
TTTCAGGGTTATCTCAAAGGTGGACTTACATTGTTTCAAAATACAAAATGTTTATTTCAGTACCAACATTAGTT
TATGATCGGAGCCTCCTTTTAAGCATAATGATGCCTGTGTTAGGAGGCTCCGGTGTTTCATTCGCCGCCTACT
CGTAATATTGAGTTGAGCAATTTATTTGGTCCAAAGGTTTCTTACTTACGTAGCGAGCGGATCAAAGTTCGAC
TCCTATATCTAGGCATAATAACATAAAATTAATGGTTTAAGATTGCTCAAACCGGTTCAGAATAGCATGTATGTA
TTTCTAGGCAGTTTTTTCCGCCACTTCTTAGCAGTAAAACATTGTTAGAAAGTGATGGAGGATTGACGTTTG
AAATTATTAATTTTAAATTGTTGAAGCTCATGGTACAGTCAACTTCAGGTTGGTGGTAACAGTTTTATAGGAA
AGTCGTACTTATTACTATTGAGTTAAGGTGCATGACAGTTACCGTATGTGCATTCTACCTTACTACTCAACTGAT

TTGTAAAATTTTTCACTGATAGTAAGCTACATTATACCTGAGTCACATAGGCTATATTTTATCCAGGTGCGGTAA
GGTAGTTCCTTCAGGACGCGGGTGGTAACTCAAAATAAAGTTAGAACATTTTTTTTCCAGGTTGTTCCTGCA
TGGTGACCCCTCAATC
>HaOG200298 UGT39B2
TTTTCATAATAGTACGAAATGACCATGTGCAATCATACCAAATCTTCAATCAATCATAAAACATGATTTAGCAGT
GGATACTGGATAAGTCTTATATTGGTTGCTGCAACCGAGGTAAATCTCGAATCTTGGGGGAAGCCTGCCTATG
CCCTGCAGTAGTGAATGTCTTTGGGCTGAGACGACAAAACGATTCACAAGGCTAAGTTTTATATATTTTGTAT
AAGTATCCAATAACTGAAACTAAGTACGTAAAGGTAAGTATTGTATAGAAGAACGTTTGGCATATTCTAACCA
CACGCAAGTTTCTGAGCAAACCAGCTTTTCCTTACCAAGTAATGTCTGAGTTCGATCAACTTAATGCAGATGG
CGTTGTTGTAAAATTAAGAGAATCTTAAATAAGTACGGAGATACAATTCAAATAAGGCCAGCAAAAATATATT
TTAATAAACCTTTAAACGTTAATGTTTTCAAGGAAGTTAAAATTATAATTTCTTGCCTGAAAAATCATTTTACTG
AGGTAGCTGGCAGGCTGAGAAGCCCCGGCAAAACAGAATAAGCTAGTACGAAAAAAATAAATGCCTTTATA
TTTTTGATGTTTAGAATAATCTTTATTAACATACACATAGTTACTGAAAACAATGAGCAAATACCGCCATTAGGT
CGACTATAACGGCCAAGGGAATTTAATATACAGATATTATTATGTTAAATACGAATATTTCTTACCCGAACCGTC
CCTAATCTTAATATAATGTTCAGTACAAATTGTTGACTAAATAGACCGACTATTGTTAGGAAAACAATAATACG
GATATGAGAGTGGATAACAATTAAAGGGTATCATAATAGATATTTCGGGAAATTCGGCCATCTTAACCTTTCG
AGGCTTATCTTTTGGAATAAATTAATATTTCACATAAATATATTCCACAAACTGGATCCGTGAGGATGGAAGGC
GACCGAGAGGGAGACCAAGAAAAATCTGGAGAGAAGACTTGGACAGCTTCCTCTCGGACTGGCCACAAAT
AGCGATGGACAGAGAAAAATGGAAGGCTATGGGGGAGGCCTTTGCCCAGCAGTGGGACAGCATAGGCTA
ATAAAAAAAAATAATTAAGTTAATAATATTGGTAGGTACGACAGACTACTATTCATAATGTGTCATACATCAGA
GCCTAGGTTTTAAACTCGGTCACGGATGTCATATGATGGACCACACTTAGGACCCGATGGTAATGTCTCCCAG
TTTGGCAATCGCTCAGTTGGTCTAGATATTTATTTTGACGGGTAAAAAGGTTGTAAATAAGCACATCAAATCG
AAATCAATACAGCGAAACAAATATTTAGAAATATAAGACCATTTTGGTCCAAGTTTTGCTCAGAAATAGAATC
GTTAACAGCGTATTTCCAAGACCCACACACGTCTAAAGAGGTTACAGATGATTAAATCCAGGAACTGGCCTT
TAAATGGATGATAATCTGGAAGAGGGCTGCTAATGAGCACACGACTTTATTCTATAACCATCGCGGAAGCTAA
AGTGGAGCGAGGATATGAGATGCTTGGAAAATATAGGAGAATTCACATCAGAAAATTAAAATTATATTTTGA
AATAAAAATTTAAAAACAATAAATTAAATACAAGGAGAATAAATGAAATAAGCGGGTAGGTACCTAAAAATTA
TCACTATATCGCAAAAATAAAGAAAAAAAGAAGAAAAAATCCCCCGTAAATATAAGCTTTTAAAAATAAAAAT
ATGGTAATTATCGATAAAGTTCTACCTATAAGTAAAGTTGAACTGCGACATAACAGCCGAGTTCACTGTGGAA
ATGTTTTTATCTAATACGCTAGGTTACGTTCAATGAGAGCGTGACAGTGACCGTCATTCTAGCGTAATGTATCC
TAGCCGCGGGTAGTGTCCACTTATATGTACAAACAAACTTCCTTTAAACTTTTAAATATTAGTTTATTTTATTTT
TGTAAAAAAATTATTTAACTGTCAA
>HaOG200259 UGT44A2
AAATTACTCTTATGAAGAAGAATAATCCAGGCCTCCAAAAAAATACTAGTTATGTATTGGTTACTAGATTTATAT
ATTTTAGTATTTCCCAGAAACTTTTCAGCTAATTAGTTAAAATTGTGGTAGCGTTGTGGTAGCCCTGACTGAAT
CCTTACGACGGTGTCTGGCCGATGGCGACATAACAATGTATCGAACAAACAATCCGACTGACATGCATTTGTA
TTTATTTGATGATCAATTTATAGCATTCAATTACTTCAAAAGAGGTTAATTGATTCAGTCCTGGTACATATTACTT
GAATCACTGAACAAGTGACGATCTGCAAAAGTTTGCTAGTAGAATTTCGATGCTGCGTGGTTCCAACATTCA
ATAAGATGCTGGTAATTATTACATCAAATTAATAGTACATGGTTAAGTGTTTAATTATATGGACTTTTTTTATTTA
CTCCTCTTATTAGCGTATATCGTATGTATTAAGACTAAACTCACCCAGGGGTTTCAAACCAAGATTAGTCAGTA
TTGTCTGACTATTATAATCAAATGCGTATTTAAATCTCGCTTTCGGAATGTATGCAAATATGAACGCAAATACCT
ACTATATGTGCACATATTATTGTCGCCTCTCACGGGTATTATTTGTGAGCACTTTGTTAGTAAGACATAACTTAT
GACGAAATATCTTCGTAGGTTTGTTATACATGGTAAAGTAATAAAACCTTTTAGACCAAAGAATATTAATTGTT
CATTGACGAATGATTTGTGCAGAATTTTCTGGAAACTCCTTCATGAGTATGACTTAATTTACCTTTAGCATTTT

AATTTTTTGTGGGACCGTGTCCTAAAGTCTGAACTGACTGAGAAGAAATTGAAAATCGAAGAAATCACTGCT
AAATATTTTTACGTTTAGGTATTGCATTACTGTAGATCAGATTTTCCTACAAGAGGTAAATTATATAATACTGCG
ACGTATTATAATAGTCACTAGTCAGTTGGGTCAGTGACCGCACTATACCGTTCGAAGCCTACTTTCTCCGTTTT
CTTTACGATCCGACAAACTTTGAGAAGACAACTTTTGTGGCAACTATTTACGCTTTAGCAACTCATAGAATTA
TTTTCCGTTTATTGAATTATAAAACATTAGCCAATGATTACTAAAATGAAACAGAGAATAACTATTAATTTTTTG
TACAAAGCAACCAAGCTTTTGTTTTTACCAAGGTCTATTTTTTGTTTAAACTTCTAGAATGAACATTTTGTGTT
GTTTACCTAATCCGAGAATCCTGCCGGTATAATGGCGTTGGCGTCAACCAATCAAACAGACATAACTTCATCC
GTGAGAGAACAACATACGACGAAGCGTTCTAATGTGTGTTCGTTTATTAAGCAGCTCCGAACAATAGCATTG
TTTGTGATGCAACCTCGTAAGGTCGCGCTTGGCCGCCCCACAATAGTTTCCAGTCTACCGACCCCCAAACATA
TAAATAAGCATGACAGCTAACGTACCTACCCTAATAATATAGTTATACTGCTAACAAATATTATTAAGCAAAGCT
ATGTTATAAGAAAATCGATATCATTGCAACTCCAGCTCCGCATTATGTCATAGTTGAACGATCATAACGAGCAC
GATACAATATTATGTATTGATAATGCCCCAACGACAATAACTCTGATAAACTATCACAATCTGTAAACATTATTGT
ACCGCAAGAATGCATACCAATCTGCATTTCATTATGCATTCATATTATCCATACAGCAGTGCACAACACGACAC
AGCTGCAAGCGCACGCGCGCTCAAAACATGTAAATCGAGGTCATGATCAATGACTTGTGTGTAGTCACCGAA
TATTTTTATTTCTTACGAAACTACCGTGCCGTCAGCGGCATGCTTACCTCATAACATCTACCTATTATTAGTCAG
CTACGTAAGCCGGCTTCCATTTACGCCCTAGTAACCAACAAACTGTGCGGCAACACGAACGCGTCCGCATCG
AA
>HaOG200256 UGT43A1
CGTTCCAGATATTCCATTTGTTGTATTGATTACTACTTATGTATAAGAATTTGACATTACTTGTACTATATCAATAT
GGGGGGCTAATAGCAAAATTCTATTTCTAGCCAATTAACGGGTAGAATATTGGACACGTCCGTAAAGTTGTT
GCAGTTAGGTACTTATAGCGTATGTAGAGGGTACGCAAAAATATTATAATAACTGAAATACGAACTGGAATGG
CGATTGTTTATTTAATTAATTAAAAGCTAGTCATATCTTGTAATGTGCGAATGTGTTATTGTGTATAATACCATTT
TTTTCATTCGATATTTAATGTACTTTAGCAGAACGAGTACGTATAAGTAGTCCATAATTTGAGGTGTTTATAGAT
GTAGATGAGATTGTAGATGTTATTAATTCTAAGCAAAAAAATTCTTCACCGTGGTACTACTTATGTACATTTCG
AAAATCCTTTCATATTGTCTGTTTTGCACAGCATGCACACAATACTCCTCTTCGGGCATGGTTTGTAACATTTC
TGTTCAGGCGGAGTGAAGTCCAGTACAAAAGATGATTGAGAACTGGTGTAGTCAAAATGGTAGTCATTAAA
TTTAGCTTCTACACTGTACTTGGGCGCAGGAGAAGGCGGTGCTCCGGGAACAGGTTCTTCATCGTCAATGTT
AAGTACGTATTTGAGAGGGTAGGGTCCTGAGGGTGCATTGCTTATGTTGGAGGGCTTGGTGCAGATGCTATA
GTTGCCAGCATGCCTGATGATCCTTATGAGAGCTTGTCCGCATGGGGGTTGCTTGCAGCACGACGGGGAGG
ACGGGCAGACCGTGACGCACTTGGGAGGCAGGCAGCAGGGCTTCACGCACGGCGGCCGAGAGCATATCG
ATGGGCAAGGAGTGCACACTGACTTTACGCAGTTATGAGCCTCGATCTTTCTGAGAACGGGGCAGGAGATG
TGACCTGGGCGCCAGCCACATCTCAACTGAAAACATCATTTAATTACAAATTAGGTACGACATAGGCTTGAAA
ATTTGATCGGTGACTTTCAGATGTTCTATTCTTACTTGATGTTCAGGGGCGTCTCTGGCGGTGTACAGCCATC
CCATATGCTTGGGTACTGGCCCCGGCTTTATTTCCTCTCTGTCTAAGCATCTGTGTTTAGGTAGATCGCAGCG
GCCCAAGCAACTCGCCGATGACTTATTCGCTACAATAATATTTAACGGTTATTATGAGATTTCATTGTATAGCCC
AGGCTTTCAAGTTAAGTTTTGGCTTATGAAAATGCCGTAATACTTACATCTACCAGCTGTTTTACAACAACAA
GCCATTTTTATAATGTGAGGACCTGTAATAGACAGTTATTTCAGTAGCCGCTATATAATTAGGTACCTATTTTCT
TATAAATGATGCATTTATATATCTAGTGTTACGTCATGCCTAATATACAGGTTTCCTAAACCTGCAGAATTTAGA
AAAGCAGGCATTATTTAGTGTGTATGTAAAAGACTCACTTTTATGGTGGTTAGGTTTCGGAGCTGATCATTTT
TCAATCTTGTTATCTTAGCCATTAGATAGGCAGGTAGTTATTTTTCGTAACTGTTAACATTTCACGTGGTGTAAT
AAATATGTTAGACTATTTCTGGTTACATGATTGTGTTTTAGAACTTCACATGACAGAATGTAATGTCTAAAATTT
TGTTCTTCGAATACTTTGTTTTTTTTTTTTATCGTTTACTTAAACAAAACTTCTTTCTCAACTTACGAAATACCTA
AGTAAACTATAATACGCACTAACTTCCTACAAATACAGCACGCGTACGTTTGTATTTGTCTTAGCATGATAAAT
GTACATATTCATACAGTCTAAAATCGTAAAAGAATGTCATGATGTCAAGGCACTCAGTGTTATTGTAACATCAC

TGTGGACTAGTCGCGTTGTTAACCAGTGCGCGTAACTATTTGCATGATAATTTTCCACGTCTAGCCGGGCGTA
AACCCGCAGAGTAAA
>HaOG200260 UGT40D1
TGCTGTTTAGAGCACAATCGTGAATTGGTTTATAAATCCGCAATTGATATTCATTGTCATCTCATCCCTACCTAC
TAAGCAAATAATGAGCATACTAGCTGCTGTCCACGACTTTGTTTTGCGGAATTTAACCGAAGGGACAAATACT
GGGTCGAGAATCTTTGAAAATAGGTCCTTTTGCTGCGCTTGCGGCAGATGGGAGTGTGAGACTCTTATACCA
ACTAAACTCAACCACGTCCCTATTTAACCCCTTCGTGGTCTTCAGAACAATCTAATTGAAACTAGCTACTAGAT
ATGTATTTGAACCAGTTGGAACCAGTTTTCAGCAGTAGGTATGTGATGTTAGAGACCATCATCACCTTATTATA
AACGTACGTGGTAGCAAAAGTACTTTTGTACCACGTTTAAACCACAAAGTTAGTGGACTAAACCTATTATGAA
AGCGGGTACTTCATAAAACCACGTTTTATAAAGTGGTCAAGGTTTAGCTTTCGCCAGACTTAACTCATGTCCA
GTAGGAACTATTCCGCGCACATGTACATCTATAACTAGGCCACCTACTTAACAGTGAAAGAATTTTTCAAATCC
GTCCAATATTTCCTGAGATGAGCGTGTTCAAGCATACCGACAAACAAGCTCTTCAACTTTACAATATTATTGTA
GATTAAATAATAACCAGTTCGCTGTCCTTGATCTAGCCACTGGCCGCCTAAATTGGACAACACACCAGTAATA
AGTACCTACAGTAGTAGCTAGCCTAATTCACGACACAAGTGGTTGCCTCCATTCGATACGCGAACTTAAAGTA
ATCTTTAAATAAAATCGTTTTGGAAATAGGTACAACTAATTACATCAGTCCGAGTTAGATTCTTATTAAGTAATC
AGTTGATATAGGCTTTATCATGTATTTAATCTAGTGATAAGTCAGGATTTGACGTATAAAATACTCAGATGGCTG
TTCTATTATTCACAATATTTTCAGTGTCTGCAGTACGCTGATTTTCAACTACAGTTAAAAGTGCTAGTTGATAA
GATATTAATGTTGTAATAATTTGAACAGCTGAGAACGTGGTTGTTTATTTTGTGATCAGTTTTATTTTATGATCG
TTGTGTAGCAATTGTATTAGGTGAGTTGTTTTGGTAATTAATTAATTAAATCATTAGTGGCATTCACGTCTTTTC
CGGTTACTTATTAATCAGTAAATGATAAATGTATTTACGTTATTCAATGTTTAATTTATCAATGGAACAAGATTTT
TTATGTGAAATTAATTAATTTAGATTACAGGATTTCTTTAGTTTAATATCCTTGAATAAACATGTACATATGTAAA
TCTGGAACTGGTTATGGCTTCTTATAGGTAACTCTAACCGGCGCAAACGGGTTCAATTGTAAGACGTATACCT
CTATACCTATTAAAAAAATATTACAAATAGGAATACTAAAGTTTAATTAAAACACATGTTTTATGACAACAAATA
AATACTTATACAGAATTTTATACTAGGTACGTACATCAGATCCCAATTAACGTAACTATGTGTTTTATTATAAGGA
TTAAGGAACCAAATCTGGTATTAAAGCTAATCTTGCGGACATATTTTTTACACGTAATCATGACATTGTATTTCT
ATTTTTAATTGTCCGGTAAACACATAAAAATAGCAAAGATAATTATTGATTGAGTCATGTTTTAATAGTGATATA
GGTACTATGTATTTATATTAATCACTATGTGATGTATAGGTAGGTAATAATTTTTTTTTTCATGAAACTGAGAACA
TGTAGATATTTTAATATTTGAGTTGAAAATTGAACGTGAAAATACATACATAGTAGGTAAGCAGTCTTAAGAA
AATTAGCGTTATGTTTTCGATGAACTCGGGCCTAAGGCTAAATATATCAGTTTAATAGGTGGACTTAATTTTGA
GTGATACTAAAGTTTCACACGATACTGGTTATTAAAAAAAAAACATAAACTTTATTTTTTTCCAGACT
>HaOG200264 UGT40M1
TAAATTGTGAATGCTAAATAAATGCATTTTGGAAATAAATAATCTTTTCTTATGGAGAGGTGCAGGATCATCTT
TCATTACTTAATTAGATCTAGTGTCAGATCTCTGAAATCTAGCTGACGGTCTCTGATGTGAAACTTTGAGAAG
GATTGCCGGGTATCACTCTTAGATATCTATCAGAAAGCTGGCTTGCTACTTACTTTCAGACTCCGGATTGCGTT
GTGAAAATTTCTGAAAACCCCACTTTGTGGATCAGATTTGGGCCTGATCGGAATCGAACCTGGGACCCTGTG
AAGGGCATTCTCTTCAGCTTTATAATATTAGCATGGGTGATCCCACGTTTATAAGCCATATACATACATGCCCAC
GGAGAACAAAATGACGAGCGGATATGTCATAATATAGAATCTCCTAAGAAAGCAGCGTTGCCAAAATGTGG
GTGTTCGGGGAACGAAGAATGCACCATTCCAACCTCACACGGCTTCTTTGGAACCCGCCCCTTTCATAACAC
CAAAAATCGGAAAATGTATCAAGGAACCCGAACGACTCGTTAGTAACGGATCGTTTTGTGCACACCCACCGT
ACGTATTTGACCTAAAAGAAGGCGCCTGACCTACCTGCATTATAGTATCTCCGCTCATCTTTTCTTACTCCGTG
ATGATAAAGTAATAGCATGGTGACTGAATAACTGGAGCAATTCACTTGCTACCAGTAGAGCATCTACCTATATA
ATATAGCTAACACATAAAGTGGTCAGATTCATTGCAGCGTCAATTTAAAACGATTGAAGGTTATAAACTACAC
ACTTTGTCTGTATATTTGCGTCCGAAAAAATAGGAGGGGGACCACAATTTATTACGAACTGACTGTTATTGTA
AGATGACTTGAAAAAGGCTTGATAGGTGGAGATGATTTTGGTTTTCCGTTCATTCATTTTATGAATCCCTAAA

AAATAATAATCATTTTACTTTTTATCGTCCTACTGCTGGGTACAGGCCTCCTCTTACACTGAGAAGGATTGAGC
GTTAATCACCACGTTCAATGTGGGTTGGTGATTTCAGACTTTGTAGCCCAGGTATCCTCAAGATGTTTTCCTT
CATCTTCAAGAAATAATAATTCAATTATTTACTTTTTTTGACGCTTTCCTGGCAAAAATATTGATTTTGATGTATA
TTGCAATTTGTGTGGATTTTAAACGAACTAAAACAGTTCATTCGAGTATTGATTCAAAATGTATATTCCTCTCA
CAGGTTACAACAAAATAGAGTATGTTTTCTTAGCACGATAAATGAGCATTATTTATGCATTACCATTGTTTGAA
AAACTGTTATTAAAAAAACATCTTGGCTGTCGGGCAAAACAAAAAAACCGTCAAGGTAATTGCTGTATCATG
TATTTTTTGGTGAGTTAGTAATTTCCTCTATCCTAAGATTTAACAGACAACTTAAAACATTTATTTGTAGGTAGG
TATATACAATGAGCATGTGACCATCAAAATCATGTCAATAAGATGACTAGACCAAATCTTTCTTGATAATATTAA
TTTGGAACATTTTACCTCCACTCATTATCACAAAACAAATACCCAAATAACTAAAAATATTCATACCTCTAAACT
CTTAAGCGGAGATTCCACTAACATAGTTTGCACACATTTTAAAAATGTGCTCGCGTAATTTGTTACTACTTGCA
AATGCGCGAAGTAGACCAGACCAGAAGCAACATTAATTTGTGTGCGTCACAGTGCCACACGCCGGTGGAAT
CTCGCCTTTATTTCAACGGTACTCAGTCAGATGACATAATATAACTACCTATACCTACATAGATAACTCTACGTAT
AATAAGCCGCTATGGTTCAATCATCTGCGCTCAAAAGGAATCAGGCGCGGGCGCACTAAACACGCGCTTTCA
ATATTTAAAAACAGAACACATTCGATATTCGATATTTAAAATAAGTAAATGAATTCTTCCGCATTTGTTTTATAA
AGACGTGTTTTAA
>HaOG200101 CYP6B2
GTGTCAACGTTGTCCCTAGGAAGCTCGTATCTTAAATATTTTGGCTATAATGATGGTACAAGTAATGGATATTAT
CTCTTCGTAATTATTATATCTACAAAAAACATTGTCGTCATCAAATTTAGTTTAAGTAATAAAAGAAAAACATGT
TACTAAAGCGTATGAAATAAGTAATGTAGAATTTCGGTAAAAACTTGACTGATCTAAATCAATTAGTGTGTATT
GTACATAAAGCTAATATAAAGTCGTACAAAATTATGTAAGCCCAAAGAAAATTTTGAGAGTAATTGAAACCCT
AATTGCCCCTGTGTATAATAATCTTAATATTGGCAATAGGAACTAGGCAAACATTTACCAATGTGTATATTTTTA
ACATTACATATATTTAGCGAAATAATTTTGTAAACTAGGAGTTATACTCAAAATAAAAAGAAGCACTGAAGTTT
ATTTTTACTTATTTATTTTCATGCTTTATAACACTCCCTTATTCGTCACAAAAAAATATAATTTGAAGTATCTTCTG
ATTCTGTCACTGATTCTAAACTTCCTCACAGCAGCAGCAGAGCAGAAGGAAGCTGGCACCCAACGTTGATG
TTTTCGTGGTCTCATATACCATCGTTCATAGAAATTATGCTAGGATAAATCAATACTGAGCTGTAGTTTGATTGC
ATAATATCAAGTACTGATTTAATTGTCAATTCTTATCCACTTTTCGTATGGAAGTATCATCGGCCAAAGCCGCCT
TAACACTGATAGTCAGCTGCATCTGATTAGACTGGCAGCCGACCCGAACATATCGGAAAAGGCTCGGCAAAT
GATGACATATATGTTTTCTATAGCATTCCTCACGTTTTATGTTGAATATACAGATATGTTATCTTATAGGTATCTTA
CTGATCTTATATCTTTTACTATCTTATATCATCCTGTTAATACTTTCGATGGTGTAAAGAAAGTTTCGGGTGCAA
GGTTGGTTGGCACAAAACATTTACCTAAAAAAATTATTGTCCAATTTTGATGCGATAGTCGGTGGTGGATTTG
TATTTCATTATTCTATATTTTTATTTGGGTGTTATGGTGTAAAGTTCAACATCATGGGATAATGCACAAAAAAAA
TGGCCGTAAAAAACCTCAACTACTGCTACTATAATGTAATTTTTTTTTACGTTGTTTTTTATCTCCAGTATAATCT
AGGGAAAATTATGATTTTTTAGTGTCGATAATGAAAAAAAGGTTCAGTTACCTGTGAAAAATTCGAGACTTA
AGACGATCCAAAAGGTTGTTATAAATATACCAATTTATAAAAAGGTATTATAACTTTAAGTTACTAAGGGGCAG
GGGCAACACTCATCGTGCAGTTTTTAAATCACGTTTACAAAACATTCAAACACCTTCCACGATACATCATGTTA
TATTGATATCATGTACATACATCATTTTGATACCTATATCTTTTACAAAATTCCCCTTATTGATAGAAAATCTCAAA
ATAATTGTTATTTAATAGTTGAACTGATAAATCTGAAGGAATAGATTTATCTACTAATGTATTTTTATTAAAATGT
TTAGAGTCACTCTATTTAAATAAATAAATCTGTGTATTATAGATATAGCTTATACATATGTATAATGTTTAATGAAA
ATGATGTTAGTGATGGATCTCTATAATAATGAAAAGGTTTGGTTTCTATTATGATATTTTACCTTGCCATTTTGG
TTGTTTTGACGAGTGTGTATAGACAGGTAGCAAGTCTCGGACTGACTCTAGATACAACTGAAGGACACCAAC
TATATCTAAGTACTCATCATCATTAACTGAAAATAAAGGTAACAAACCAATATTTTTCTGCACACCACTTGCAGT
GTATAGTTAAAAAAATGTTGTTTCATGCATGCAGCATTATCAAAAATCAAGTCTCTCGCTAAGTATATAAAACA
GGTCGAGAGATATTGCTATTCATAACACAGCGTTCGGTTACTAAGATCACAGTTCCTCCAAA
>HaOG200103 CYP6B6

TACTTGTAAATTACCTGCCAAAGTTGCTGATTGGTTCGATTTTGAAGTACAATATCTTCCTAATAAGTACCTTAC
TTTAACGATTAGTCTATCAAAAACATTTCTTTTATGGGTTTACTACTTTTATTTATTATGGGTATGCGATTCAAA
GTATAATTTTTGGTATTAATGAGGTGTAATAAAGCAATTAAAAGACATGCAATCATCTTATTTGTTAAAAAAAA
CTAACCGTTTTCCCGAATCCCGGGGCAATTATTGCCCGTAGTAGGACAAAATATAGCCTATGTTTCTTAGGAA
GAGGTAGGTTTTTAACAAACAAACAAGAAAAACATAATTTCCTCTAATAATAGGTATAAATATACAAAGTATAT
CGTACCTAACCACGTTACATTAAATACGTTAATATACTGGTTAATATATGTTGATTAGCACAAAGAAAGAAACA
AAACACGCAAAAATAATAATAATAAATTCAAACAAATTAAATAGAATAAAAATGTGCGAGAATTAGAGCACCA
TATAAGAGTCAGTCATTACAAACAACGGAAATTCTCCCTTGAAAACTGACAGCTGTCAATTGGTTAAAACTTT
CGATACTCCTAACTTTATCTCTCTGTCTACACATGATGTTGTAAATTATGAAAACGAAAAATCACTCCTGTGGC
CAAACCATGAACAAATACCGAATGGATTCGGTTATTAGGATTTTTTACAATTCAATGTGATTGGGAACGACAC
ACCCGATATAGACAACATCAAAAGTTTGACCAATACTATTTTCTTAATTATCAATTAATTTCATGAAGAAAGTGT
GACGCCTTAGATATTTTTCGTACCTAATGATACATTTAACGAGGTTTAGATATGGTCATATAAGAATATTGAGGT
CGAATGGCAGCTAGAATAAATTAGATAAATCTATTAATATTTATTCTTGACATACATATAAGGGAAGATAACCAT
TTTCGTACAGTACTTTTTATCAAAAACGCGTTAGTGTGAGTTGCAGTCAGTTCTGAATATAATGTGGTAATATAT
ACCAAAACTAAAGCTGGCAATCACTTTTCACACTTAGCATGACACGTGCAAACGCCAACACAAGTACGTCTG
CACTAATGCATATCATAGTAACAACTCATGCTTGCAGCTTTTCTAAATTTTAAAAGAAAACTACGATAACACAC
ACAAAAACAATAATTGTTTGTGTAAGATACAACTAGAGATTTTAATTTGTGCTATCGTAATCTTGTTTGATGATC
AGTAGGTATATAAAATACGCCGCCTTAGGTAGCTCTTCATATCATTGTGAACAGCGAGGGAGTCT
>HaoG200104 CYP6B7
TATGTAAAATCTAGACCTGCAAGCTACAAACTCATTACTTTGTTAGTTCAGTAACAAGACAATAAAGTTACATT
CGTGCAATGCAATGTAAATTAACTGCTATAAGTATTAAAACCTAATATAACTGGAACTACAGTTGAACTCATAA
GATATTAAATGTTAGAAGAGATAACAACATCAGTCATTCAAAGATTTTTTTAGCGTCACAATATCCTAACATAA
TGATTGGACCTGTTATTATTCATAACAAGTTGTTATCAAAATGCACGTGGTGTTTTGGAACTTGTTTTCAACTA
TCAATACATAAACATTTGAACGATATATGATCAAGACATTGAACCCATATTATCAAATATATTTAAGCCGTTTCTT
TGTCATCATCATCTAGCCTTTTCTCAATAACTTTAACATCACAGTTACTCGCTGGTTAAATTGACGCGAGTACTA
GGTAACTTGAAACACCTTAGTAAAAATAGTTGTCAGACTATCTCTACTAATGAATGCTGGGCTGTCTGCCAAA
AAAATATGAAGGAGTGCGTTTTAAATATTTTTTGCTTTTAATGTAAATTTTTGCTAAAACCGAGCGAATATTTG
TTTTCCATTCATGGCGAAGATGCTTTCATTATGGTCAGCCTCGATCTCATTCAGCTTTCGCTGGCGGCGACAA
GTCTGCCAGTTTCCTTGTCTAGCCGTCCGCACAATGTCTCCCCTGACAACTCAACATGGCTATAAACGAGATA
GCTCATAAAAGATTTTGAGTATTGTCGCCAAGCTTAAATGATAGATATTTAAATCTTTGGTATCGATAGTTGTTT
CAGGTCCAATCTTATTTTCAATTGGCCATGGGATAAAATGTAATCAACGACATGCATGAGACTCATTATTATTG
AAGAAAACTCGACGATACTATTTGAGTAGGTATAGATATTGCTTTTAATCATTCAAACGAATTCTTCCATTCAA
GATATACCTCTACATGCATCAACAAAGATACTATGGACTTTGATTTTATTTTGGTGTTCATTGTCATCAACACTG
AATCATGAGCAGGGGAATGTCAAAAAGTAATATCCACATGTTCCTTTCGGAGTTTTGGAGCCATTTTTTTCCA
TAGGCATGTATCCTCGCATTGGACGTCGTAGTCTTTTTTATGAAATACCCAACTTGATATAGCCCCGAGACTGT
TTTATCCAGTTTTGTTTTTAAACGATCCAAGAACACATTGCAATATAGGTGGCCGGTGGCTCCTGTCTCTGGG
GCCCTAACTACCAGTCGTTTGGACCCTGTTCTCATAACCGTAACACGTAAAAATGTAACAAATATAAATGTCAT
TTAAATGCTATTGAAGGTTTAATTATTCACAAAATTCAGTATGGTGAATTACAAACTAATCTAAATCTGTTCCTT
GAATTTCCTGTATATGTTTCAGCTAATCATATGCATTGAAATTACAATCCTTTCTAAGCCTGGTACCTCTATAGAC
CATGGGTGAAAAAGTATAGATCCATATTCAAGTTTTGTTAAGTGTTGCCCAAAATAACTTATAGGATATTAGCT
ATTATAACCACCATCAAAAATTGTGAAGAATTAATATGAATTGACATTTGAAATCAAAGTAAAACAAAGTTAG
GTTCTCAAAGATATCTTTTATATTCTTTGGTTTTTTAGAACTCGATTTATGCCGTCGAGGTTTACGGTGGCGAC
GTCGGTCGACTGTCCTATAATTAAAGGCACGTGCAACCACCAAAGCATGTCTGGTTGCACTGACTAGTTTGA
GCTTGATAAGTCATGACAATACTATTTCGTAAATATTATAAGATAACACACACGAACAATGCGTTTTAATAGTA

GATAGATGATAGCAGAGCGGAGATTTATAAGTCACATTATCGATATCCAGTTTTTTCGTGGTATATAAAACCAG
CCGCGAGTTTTACTATTCATAACAAGGTCATCAACGGTTGTGTAAACAGCTCCTCAAA
>HaOG215044 AhR promoter
CAAATTACCCCTTGCTAAGACTAGTTGTCAGACTTACTGGCTTCTGACTACCCATTACGACTGCCAAAGACCT
ATAGTTTATCTCACCCTCTGAAACACGGTCGTTGGTGTCCAAGATATACTTAGAAAGTACATACAAACTTAGAT
AAGTGGCATACTTGCCTTATTTAGAATCGAACCCACACTCATATGTACTTGTGGGGTTCTTTAGCCACTAGGC
CACAGACTCCAACTCTATTTCTCCTAAAAAAGGTGACAAATTACACCATGTCAACTATCCTTATTACGTGATAA
GAAAAGACAATTATATCATTATCATACACAATACAATTGCGAACCACAATAATTGTCAGCAATCATGCGTGTGC
TACAATTACCGATCGATCAACTGTTTTTATTATAATTTGTCTTGACTTGATCAGGGTTTAATATACCAATTATGTG
CGGACAGTAACATATACTTGGGTAAATAAAAACCAGCCAAGTGCCAGTCCGACTCGCGCACGTAGGGTTCTG
TACCATCCAAACTAATATTCTATATACATTTATGGATATCGAGCAAAAAAATCACGTTTGTTGTATGGGAGCTCC
CCGAAATATTTATTTTATTCTAGTTTTCAGTATTTGTTGTTATAGCGGCAACAGAAATACATCATCTGTGAATTT
CAACTCTCTAAATATCACGGTTCATGAAATACAGCCTGGTGACAGACGGACGGCCGGACGGACAGAGGAGC
GAAAACAATAGGGTCCCGTTTTACCCTTTGGGTACGGAACCCTATAAAGAGGTTGGATATTGCTCTGAAGAA
AATAATGGCGTTCATAGTAATTGAGTTGAAATAACTAATAGAACGATACATATTGTAAAGATGAGGGATCGATT
AAAACTGACTTGTATTAATTTTCTGGTTATTAGTTGTAATACTAGAGTAAATAGATGGGTTATATAATAATGGCT
GGATGCTCCGTTCGTTTAGTGGCTGTTAAGCTCTGCTTAATCGATTTCCCTCTTATAATGAACATGGCTTAAAA
TTACACCAAAGAATAACATGAAAATAGACGCACATTTTCATGTTTTTTTAACTTGTATCAACCGTTCTCCTTGT
TTATTAATAAGGGATTTGTGTGTTTTAGAGTCAGTCAATCATTATACCCCTGTTATAAGGCACACATCCCTCGTA
GTTACGGGAAGATGCAGATATAAAAATTACTCGTCAGGACGATTTGAAAAGACTCTGCCACCAAGGCTTAAA
CCTGCATCCTGCTTGATCAGAACTGTACATTGTGCGGTTAATAAATAATTCCAAAAGAAAAATATCAAAGGAG
AACATTCATTTCGTACCCAAAACTGAAAGTGCCGGCCAGAAGAAAAAAATTGTAGATTCTTGTAGAGGATAA
TGCCCTGAAGATTTTTTACTATTACAAGAATTGTCTACAATTAACCCCCAGGCTGCTATTTGACTTTGAAAATA
CCAAAAAATCCCACAATGTTTGGAGTATTACATTACAGCCCCAAACTGGAGAAGCCAGCCGTTGCCTTCAAA
CGACTTCTATTCGACCTGGGAAGTCATCAAATGACCCCCCTCTCGCCATGAGAAAGTCAGACTCTTACTGACT
AAAACCCATCATGGGGAAACCAGGGCCGCGATAATCCTTTCGAAGAATCTCGCAGCCCCGATAGGCCTTGTC
CCCGCAAAAATATCGTACTATTCTTGTCGAGGATGCCCTGAAGATGTTTTATATTGTAAGGAACGTCTCCGGTT
AACCCTCAGACTGCTATTTGAGTTAATCGTGAATAAAAATCTATACTTGAATGTTACGGCAAATAACGTCCACA
GTATTTTTTTTTACTAATCGAACGTCTAGAAACTCTTTCGAAAAATCCCGCAGCCCCGGCAGGTTTTGGTCAC
CTGGGGTTTGCTGACACTCTCTTGTCTCCAAGAGAAAGACGGAGGGACGATGAGCCACTCGAAATTCTATA
GGGGCGAGGGGGAATGGG
