# Supplementary figures and images for: A General Signal Pathway to Regulate Multiple Detoxification Genes Drives the Evolution of Helicoverpa armigera Adaptation to Xenobiotics
Source: Int J Mol Sci. 2022 Dec 17;23(24):16126. doi: 10.3390/ijms232416126 (PMC9788003; doi:10.3390/ijms232416126)

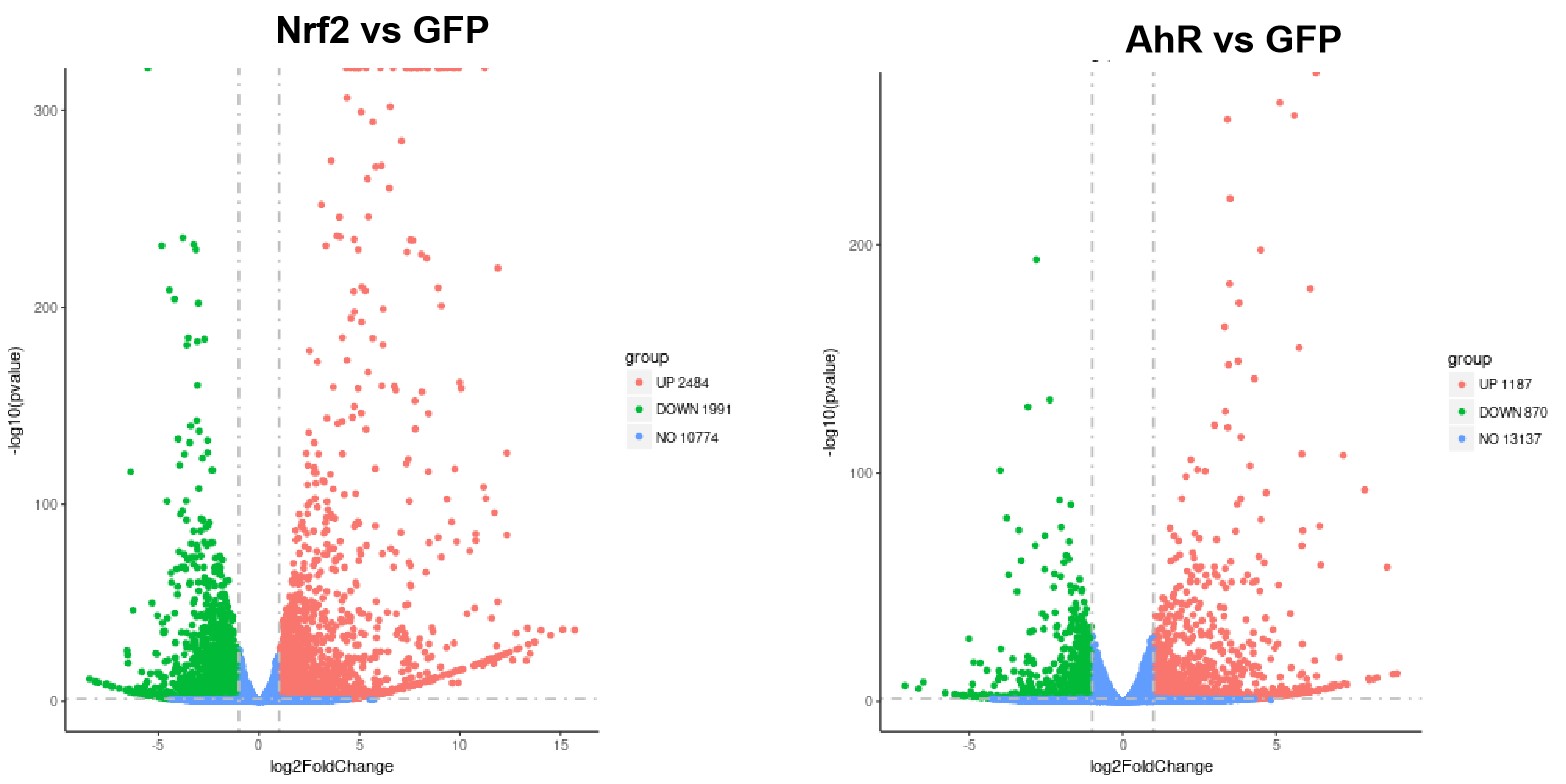

Supplement: Supplementary file 1 [file ijms-23-16126-s001.zip › Figure S1.jpg]

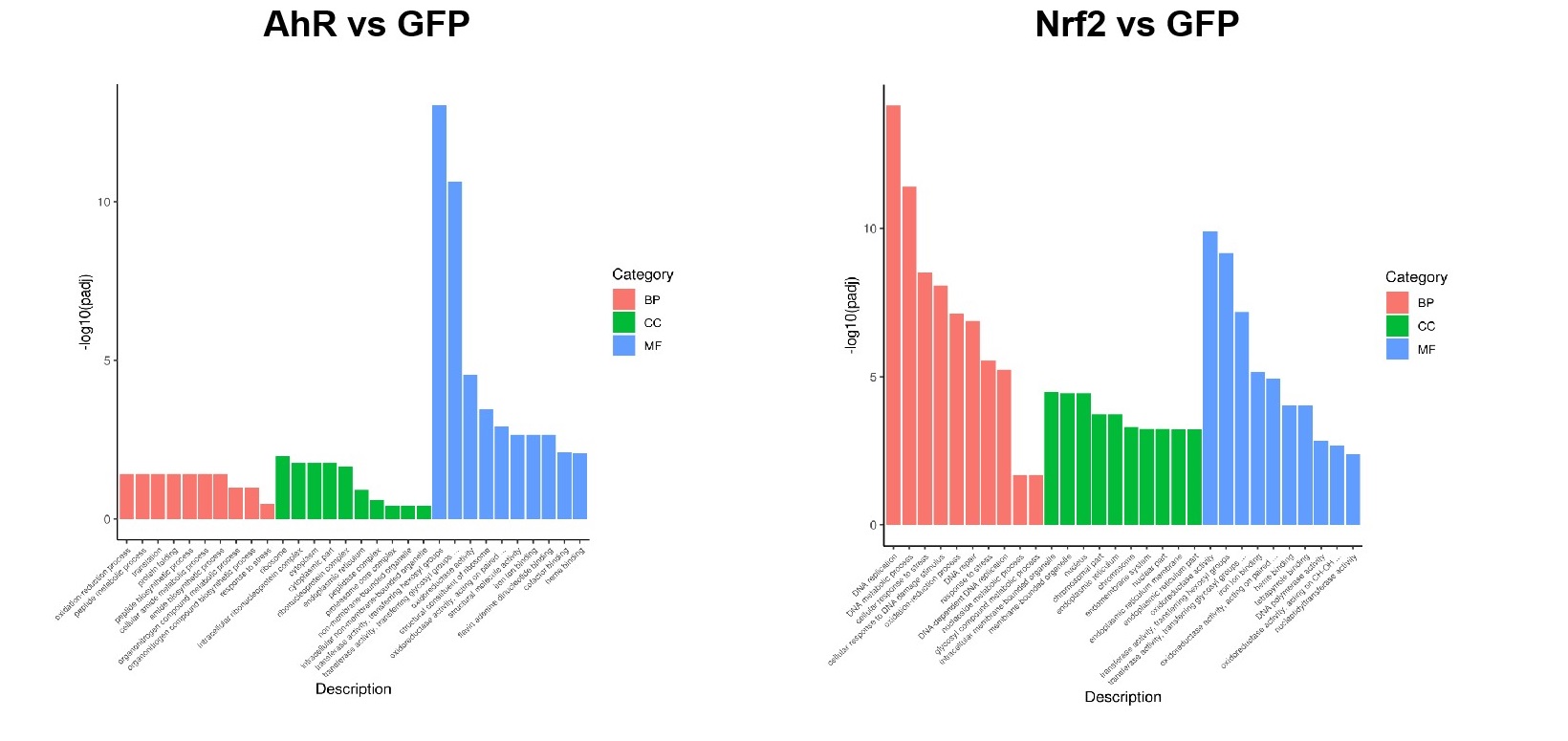

Supplement: Supplementary file 1 [file ijms-23-16126-s001.zip › Figure S2.jpg]
